# Mass spectrometric analysis of accumulated TDP-43 in amyotrophic lateral sclerosis brains

**DOI:** 10.1038/srep23281

**Published:** 2016-03-16

**Authors:** Fuyuki Kametani, Tomokazu Obi, Takeo Shishido, Hiroyasu Akatsu, Shigeo Murayama, Yuko Saito, Mari Yoshida, Masato Hasegawa

**Affiliations:** 1 Department of Dementia and Higher Brain Function, Tokyo Metropolitan Institute of Medical Science, Setagaya-ku, Tokyo 156-8506, Japan; 2 National Epilepsy Center, Shizuoka Institute of Epilepsy and Neurological Disorders, Urushiyama 886, Aoi-ku, Shizuoka 420-8688, Japan; 3 Choju Medical Institute, Fukushimura Hospital, Noyorimachiazayamanaka, Toyohashi 441-8124, Japan; 4 Department of Neuropathology (Brain Bank for Aging Research), Tokyo Metropolitan Geriatric Hospital & Institute of Gerontology, Itabashi-ku, Tokyo 173-0015, Japan; 5 Department of Laboratory Medicine, National Center Hospital, NCNP, 4-1-1 Ogawahigashi, Kodaira, Tokyo 187-8502, Japan; 6 Department of Neuropathology, Institute for Medical Science of Aging, Aichi Medical University, Nagakute, Aichi 480-1195, Japan

## Abstract

TDP-43 is the major disease-associated protein involved in the pathogenesis and progression of amyotrophic lateral sclerosis (ALS) and frontotemporal lobar degeneration with ubiquitin-positive inclusions linked to TDP-43 pathology (FTLD-TDP). Abnormal phosphorylation, truncation and cytoplasmic mis-localization are known to be the characteristics for the aggregated forms of TDP-43, and gain of toxic abnormal TDP-43 or loss of function of physiological TDP-43 have been suggested as the cause of neurodegeneration. However, most of the post-translational modifications or truncation sites in the abnormal TDP-43 in brains of patients remain to be identified by protein chemical analysis. In this study, we carried out a highly sensitive liquid chromatography-mass spectrometry analysis of Sarkosyl-insoluble pathological TDP-43 from brains of ALS patients and identified several novel phosphorylation sites, deamidation sites, and cleavage sites. Almost all modifications were localized in the Gly-rich C-terminal half. Most of the cleavage sites identified in this study are novel and are located in N-terminal half, suggesting that these sites may be more accessible to proteolytic enzymes. The data obtained in this study provide a foundation for the molecular mechanisms of TDP-43 aggregation and ALS pathogenesis.

Transactivation response (TAR) DNA-binding protein 43 (TDP-43), encoded by the *TARDBP* gene, is a highly conserved, ubiquitously expressed nuclear protein. It has conserved RNA recognition motifs (RRM1/RRM2) flanked on either side by N-terminal and glycine-rich C-terminal domains that mediate protein-protein interactions^1^,^2^. Recent studies show that TDP-43 is a nuclear ribonucleoprotein implicated in exon splicing, gene transcription, regulation of mRNA stability, mRNA biosynthesis, and formation of nuclear bodies^3^,^4^,^5^,^6^,^7^,^8^,^9^,^10^,^11^. Furthermore, TDP-43 is thought to be essential for early development, because homozygous disruption of the *TARDBP* gene causes early embryonic lethality^12^,^13^.

TDP-43 is the major disease-associated protein involved in the pathogenesis of amyotrophic lateral sclerosis (ALS), frontotemporal lobar degeneration with ubiquitin-positive inclusions (FTLD) linked to TDP-43 pathology (FTLD-TDP)^14^,^15^, and other neurodegenerative disorders^16^,^17^,^18^,^19^,^20^. Mutations of TDP-43 in familial and sporadic ALS and FTLD cases have been linked to the development of TDP-43 pathology^21^,^22^,^23^,^24^. These mutations are mostly located in the C-terminal glycine-rich region^25^, suggesting that conformational change of this region is closely related to the pathology.

TDP-43 is predominantly localized in the nucleus, but under pathological conditions it is translocated to the cytosol^14^,^15^,^26^,^27^,^28^,^29^,^30^. Thus, loss of normal function of nuclear TDP-43 due to cytoplasmic mislocalization, and toxic gain of function due to TDP-43 aggregation are potential disease mechanisms^14^,^15^.

Immunoblotting of the Sarkosyl-insoluble fractions from FTLD and ALS cases using phosphospecific antibodies clearly demonstrated that hyperphosphorylated full-length TDP-43 of 45 kDa, smearing substances, and fragments at 18–25 and 35kDa are the major species of TDP-43 26,31,32. We identified at least three C-terminal banding patterns that distinguish diseases with TDP-43 proteinopathy and reported that the banding pattern in different brain regions and spinal cord of individual patients is indistinguishable. Corresponding patterns of protease-resistant phosphorylated TDP-43 are also seen among the pathological phenotypes^26^,^27^,^32^. Recently, we showed that insoluble TDP-43 aggregates in brains of ALS and FTLD-TDP patients have prion-like properties^33^.

TDP-43 phosphorylation, cleavage and cytoplasmic mislocalization are all associated with TDP-43 aggregation. However it remains to be clarified whether these modifications are the cause of the disease. Recent reports on post-translational modifications, including fragmentation, were based on studies of cellular or animal models overexpressing TDP-43 or its derivatives^34^,^35^,^36^,^37^. However, TDP-43 aggregate formation in these artificial models may be different from that in the brains of patients. Therefore, it is important and essential for elucidation of the pathogenesis to identify the pathological events in TDP-43 truly occurred in the brain of patients. In this study, we carried out detailed molecular analysis of pathological TDP-43 aggregates in ALS brains.

## Results

**Figure 1:**
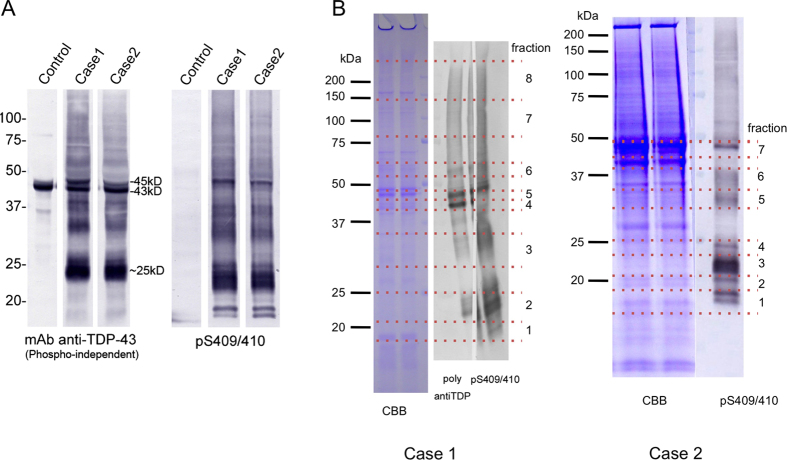
SDS-polyacrylamide gel electrophoresis (PAGE) and immunoblotting of Sarkosyl-insoluble fraction from ALS case 1 brain and ALS case 2 brain. (**A**) A phosphorylation-independent anti-TDP-43 detected normal TDP-43 of 43 kD in the fractions of both ALS and control brains, but also detected abnormal TDP-43 bands, including full-length phosphorylated TDP-43 of 45 kD, ~25 kD fragments and smears in ALS brains. (**B**) TDP-43-positive bands were excised as indicated.

Immunoblot analysis revealed that abundant abnormal TDP-43 was recovered in the Sarkosyl-insoluble fractions of brains from both ALS cases as shown in Fig. 1A. A phosphorylation-independent anti-TDP-43 detected normal TDP-43 of 43 kD in the fractions of both ALS and control brains, but also detected abnormal TDP-43 bands, including full-length phosphorylated TDP-43 of 45 kD, ~25 kD fragments and smears in ALS brains (Fig. 1A). The pS409/410 antibody, which specifically recognizes the abnormal phosphorylation of Ser409 and 410 strongly reacted with these pathological TDP-43 bands and additional fragments of 18 ~ 24 kD (Fig. 1A). No such bands were detected in the same fraction of control brain. Intensities of pS409/410 immunoreactive bands in both ALS cases were 5 ~ 10 times higher compared with those from the other cases previously analyzed in our lab (data not shown). Therefore, we thought these two cases are good for the protein chemical analysis of pathological TDP-43.

**Figure 2:**
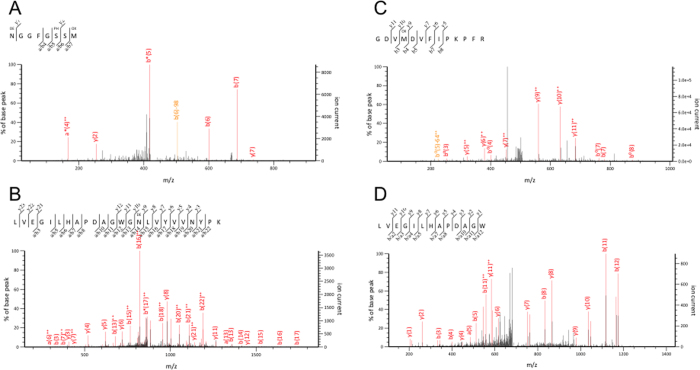
MS/MS identification of phosphorylated peptides and cleavage site peptides. Representative peptides were shown. (**A**) Chymotriptic peptide, 398-NGGFGSSM-405 in case 1. The 6th Ser residue was phosphorylated. (**B**)Triptic peptide, 56-LVEGILHAPDAGWGNLVYVVNYPK-79 in case 1. (**C**) Tryptic peptide, 215- GDVMDVFIPKPFR-227 in case 1. Trypsin can not cleave N-terminal Tyr214-Gly215 site. Therefore this site is intrinsically cleaved site. (**D**) Chymotryptic peptide, 56-LVEGILHAPDAGW-68 in case 2. Chymotrypsin can not cleave N-terminal Arg55-Leu56 site. Therefore this site is intrinsically cleaved site.

**Figure 3:**
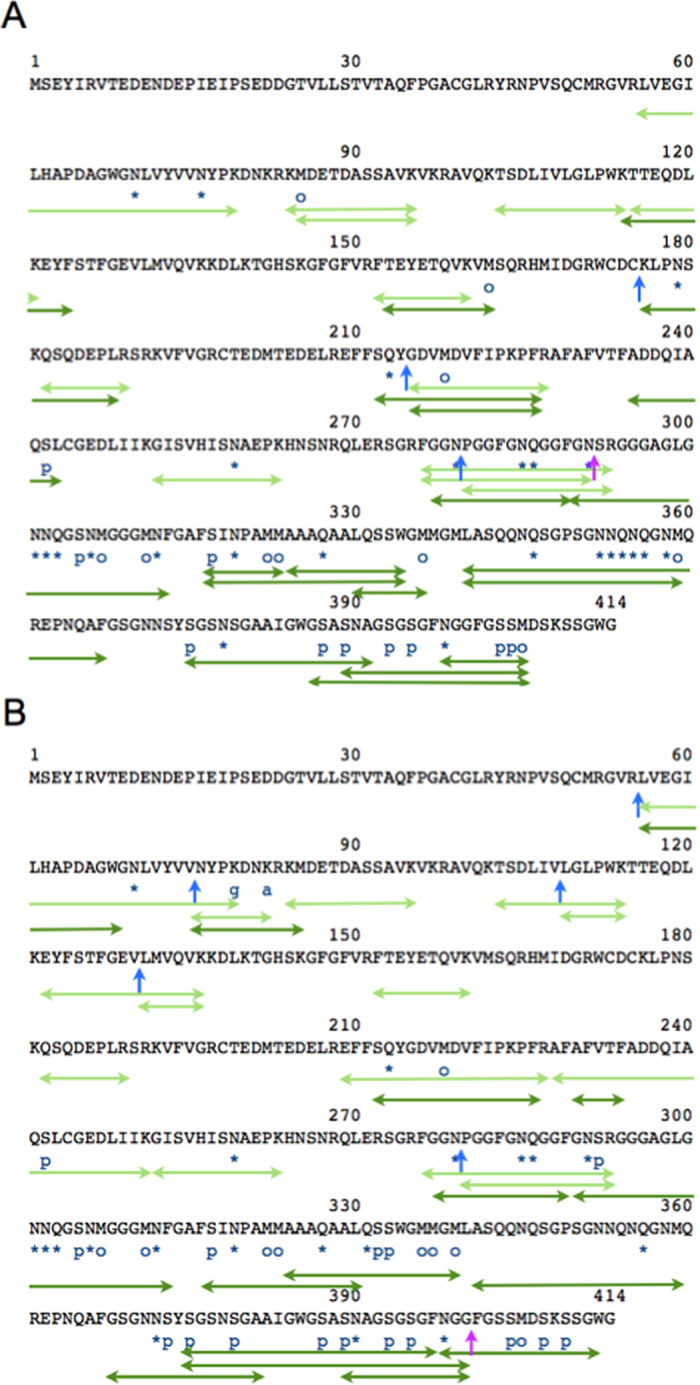
Identification of modification sites in TDP-43 from ALS case 1 (**A**) and case 2 (**B**) by LC-MS/MS analysis. Identified peptides from trypsin digestion (light green) and from chymotrypsin digestion (dark green) are shown. p indicates phosphorylation site. o indicates oxidation site. * indicates deamidation site. Blue and pink arrows indicate N-terminal and C-terminal cleavage sites, respectively.

After SDS-PAGE, the gel portions corresponding to these bands were cut out as shown in Fig. 1B and digested with trypsin and chymotrypsin. Digests were analyzed with nano-flow LC-MS/MS. As shown in Tables 1 and 2, we identified 229 peptides from brain of case 1 and 92 peptides from brain of case 2; this difference may reflect the differences in total amounts of pathological TDP-43 in these brains and the numbers of fractions. These peptides covered about 90% of the intact molecule (Tables 1 and 2). Identified peptides (representative), modifications and cleavage sites are shown and summarized in Figs 2 and 3. Further cleavage site peptides are shown in supplemental Fig. S1. Other proteins contaminating the Sarkosyl-insoluble fractions are listed in supplemental Table S1.

### Analysis of Case 1

We identified at least four intrinsically cleaved TDP-43 peptides in the pathological TDP-43, i.e., three N-terminal peptides (blue arrows, Fig. 3A) and one C-terminal peptide (pink arrow). However, no caspase-cleaved peptides were detected in this study (Table 3). Ten serine residues were phosphorylated, as shown in Fig. 3A and Table 4. These phosphorylation sites were the same sites as previously reported^27^,^38^. Furthermore, deaminations of Asn and Gln residues and oxidation of Met residues were identified. Almost all modification sites were found in the Gly-rich C-terminal half.

### Analysis of Case 2

We identified six intrinsically cleaved TDP-43 peptides in this case, i.e., five N-terminal peptides (blue arrows, Fig. 3B) and one C-terminal peptide (pink arrow). As shown in Fig. 3B and Table 4, 15 serine residues were phosphorylated and 21 Asn/Gln residues were deamidated. Moreover, we found that the 79Lys residue was ubiquitinated and 82Lys was acetylated. However, 145Lys and 192Lys were not detected^39^. Phosphorylation sites were the same sites as previously reported^27^,^38^. Again, almost all of these modification sites were localized in the Gly-rich C-terminal half.

### Common modifications in cases 1 and 2

All modifications are summarized in Table 4. As described above, almost all modifications were localized in the Gly-rich C-terminal half. Furthermore, common modifications focused on 180–330 residues region of these cases, suggesting that the 180–330 region of TDP-43 had the same structure in both case 1 and case 2.

## Discussion

Accumulation of filamentous inclusions composed of abnormally phosphorylated full-length TDP-43 (45 kDa) and its fragments (35 and 17–27 kDa) is a defining feature of TDP-43 proteinopathies^26^. It has been reported that overexpression of full-length TDP-43 with or without mutations in cultured cells and animals leads to fragmentation of the protein, generating 35-kDa (CTF-35) and 25-kDa (CTF-25) C-terminal fragments via caspase-mediated TDP-43 cleavage^30^,^34^,^40^. Furthermore, activation of calpain, a Ca^2+^-dependent cysteine protease, by upregulation of Ca^2+^-permeable AMPA receptors generates C-terminally cleaved TDP-43 fragments (~35 kDa) and causes mislocalization of TDP-43 in motor neurons^36^. Those reports noted that the ~35 kDa fragments were localized in the cytoplasm and formed insoluble aggregates.

**Figure 4:**
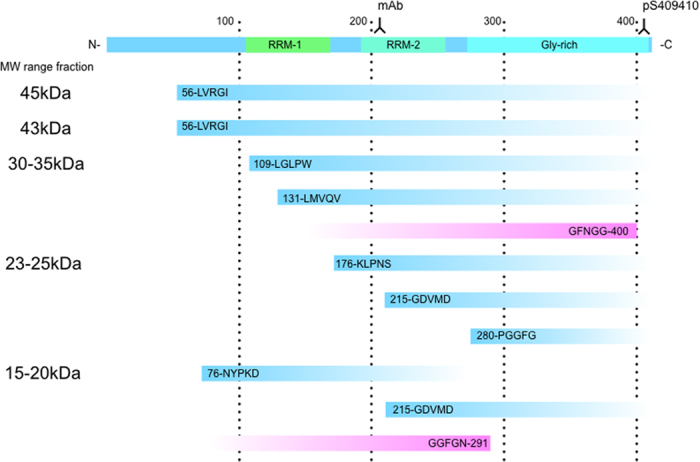
Schematic representation of N-terminal and C-terminal cleavage sites on TDP-43. Cleavage sites (amino acid numbering of TDP-43) in gel fractions and antibodies recognition sites were shown.

However, no such TDP-43 peptides cleaved by caspase or calpain reported were not detected in the pathological TDP-43 in the brains of patients in this study. On the other hand, uncleaved peptides by caspase or calpain were detected as the major molecules in the digests. Furthermore, several novel intrinsically cleaved TDP-43 peptides were identified in the pathological TDP-43, i.e., three and five N-terminal peptides (Cys164-Lys165, Tyr214-Gly215 and Asn279-Pro280 in case 1, and Arg55-Leu56, Val75-Asn76, Val108-Leu109, Val130-Leu131 and Asn279-Pro280 in case 2) (Figs 3A,B and 4 and Table 3) and two C-terminal peptide (Asn291-Ser292 in case 1 and Gly400-Phe401 in case 2) (Figs 3A,B and 4), strongly suggested that these cleaved fragments under pathological conditions was different from that under TDP-43-overexpressing conditions.

Cellular expression of TDP-43 is tightly regulated at the transcriptional and post-translational levels. Under pathological conditions, however, formation of TDP-43 aggregates within the cell nucleus or cytoplasm results in reduced free nuclear TDP-43, and therefore the TDP-43 binding-receptor sensor detects a fall in protein levels and responds with increased TDP-43 production; the produced TDP-43 is then sequestered by TDP-43 aggregates^2^. Hence, TDP-43 production under pathological conditions does not necessarily represent overexpression. It is possible that TDP-43 aggregate formation under pathological conditions is different from that under TDP-43-overexpressing conditions.

It has been recently reported that C9ORF72 repeat expansions in mice cause TDP-43 pathology. In these mice, insoluble phosphorylated TDP-43 was predominantly present in monomeric form and high-molecular-weight or truncated pTDP-43 species were not detected^41^. Further, recombinant full-length human TDP-43 forms structurally stable, spherical oligomers that are neurotoxic *in vitro* and *in vivo*^42^. Indeed, cellular aggregate formation or accumulation of TDP-43 C-terminal fragments (CTFs) is not primarily responsible for development of the observed disease phenotype in mutant or wild-type TDP-43 mice^43^,^44^,^45^. These findings suggest that full-length TDP-43, not the cleavage fragments, participates at an early stage in TDP-43 pathology. Fragmentation may occur after the accumulation of TDP-43.

We identified some intrinsically cleaved TDP-43 peptides at the N-terminus and C-terminus of Sarkosyl-insoluble TDP-43 in this study. These cleavage sites were neither the caspase cleavage sites nor the calpain cleavage sites of TDP-43 as reported previously^46^,^47^. Several cleavage sites deduced from these N-terminal peptides exist in the region of residues 55–280 as shown in Table 3 and Fig. 4. Therefore, full-length TDP-43 may be gradually processed at N-terminal sites after the formation of insoluble aggregates as shown in Fig. 4. Our findings that cleavage site is correlated with the distribution of molecular weight size are consistent with this idea. Further studies using antibodies for these cleavage sites will clarify the gradual processing mechanism.

In our previous studies, we identified phosphorylation sites by immunochemical methods^27^ and also identified casein kinase-1 phosphorylation sites on recombinant TDP-43 38. Almost all Ser and Thr residues in C-terminal Gly-rich half were able to be phosphorylated *in vitro* experiment^38^. In the present work, however, a part of accumulated TDP-43 was phosphorylated, not all. Non-phosphorylated peptides were major products from aggregated TDP-43 digests from these ALS brains. We protein-chemically identified several novel phosphorylation sites by directly analyzing the accumulated TDP-43 from two ALS patients. Phosphorylation sites identified in case 1 and case 2 corresponded to the sites identified in casein kinase-1 phosphorylated TDP-43. Although 9 phosphorylation sites were common to both cases, more phosphorylation sites were identified in case 2, as shown in Fig. 3A,B.

It has known that non-phosphorylated intact TDP-43 forms fibrous aggregate^42^, suggesting abnormal phosphorylation is not first event in TDP-43 pathology. The level of phosphorylation may change according to the time when TDP-43 aggregates exist in cytoplasm. Phosphorylation-triggered degradation is a common strategy for elimination of regulatory proteins in many important signaling processes. Phosphorylation of TDP-43 may occur in elimination process. The impairment of this process due to TDP-43 aggregation will induce abnormal phosphorylation of TDP-43 aggregate. As the time has passed, the level of phosphorylation seems to become more. Therefore, the individual difference may occur in the level of phosphorylation.

Furthermore, it seems reasonable to speculate that heavily phosphorylated peptides may not be detected because of low recovery from the column and low ionization efficiency in mass analysis. S409/S410-phosphorylated TDP-43 was immunochemically included in almost all fractions as shown in Fig. 1. However, we found small amount of peptide including S409 with phosphorylation in case 2, while we found no peptide S409/S410 with phosphorylation in case1. A peptide including S409/S410, which derived from chymotrypsin digestion, has at least 5 phosphorylation sites. We have already reported that TDP-43 C-terminal region including these phosphorylation sites was heavily phosphorylated^26^,^27^,^32^. Therefore, we might not detect C-terminal region peptide including S409/S410 residues. It is possible that there are some differences in phosphorylation states of accumulated TDP-43 and in the detection efficiency in the mass analysis between the cases.

Indeed, there were individual differences in other modifications, as shown in Fig. 3A,B. However, we found that common modifications in case 1 and case 2 were focused in the 180–330 region (Fig. 3A,B, and Table 4). This suggests that the 180–330 region of TDP-43 had the same structure in both case 1 and case 2.

Interestingly, inclusions within the brain of ALS and FTLD-TDP patients are readily labelled with antibodies that recognize the C-terminus of TDP-43, but not with N-terminal TDP-43 antibodies. In contrast, spinal cord inclusions are labelled with both N- and C-terminal TDP-43 antibodies, suggesting that they are composed of full-length TDP-43 48. This regional heterogeneity in terms of C-terminal fragment formation suggests that these fragments may not be necessary for TDP43-mediated neurodegeneration^49^. Furthermore, TDP-43 fragments generated during neurodegeneration were not C-terminal fragments, but rather were derived from a central portion of human TDP-43 45. These results indicate that a common structure region, residues 180–330 of TDP-43, is significant for neurodegeneration

It has already reported that a Gly-rich region (287–322), which contains multiple glycine repeats, may contribute significantly to fiber formation as well as aggregation propensity^50^,^51^. Recently, it has also been reported that the 274–414 GQN-rich region (C-terminal region) has prion-like properties^33^,^35^, and that RRM2 domain of TDP-43 plays a key role in forming proteinaceous aggregates^52^. The common structure region, residues 180–330 of TDP-43, includes RRM2 and a part of the GQN-rich region. Therefore, this common structure region may be the core region of TDP-43 aggregates. The identification of N-terminal and C-terminal peptides in this study suggests that most of the cleavage sites exist in the N-terminal and C-terminal non-core regions. Proteolytic enzymes may readily access these regions and cleavage may occur gradually from distal regions. Our data provide important insight into the mechanism of TDP-43 accumulation, though further studies will be needed to clarify the molecular pathology of TDP-43 in detail.

## Methods

### Fractionation of accumulated TDP-43 in ALS brain

Sarkosyl-insoluble abnormal TDP-43 was prepared from brains of two ALS cases with abundant and widespread TDP-43 pathologies. Brain samples (0.5 g) from patients with ALS (case 1, 77-year-old male, disease duration 2.5 years and case 2, 57-year-old female, disease duration 2 years) and from vascular dementia (disease control, 88-year-old female) were homogenized in 10 ml of homogenization buffer (10 mM Tris–HCl, pH 7.5, containing 0.8 M NaCl, 1 mM EGTA, 1 mM dithiothreitol). Sarkosyl was added to the homogenates (final concentration: 2%), which were then incubated for 30 min at 37 °C and centrifuged at 20,000 g for 10 min at 25 °C. The supernatants were centrifuged at 100,000 g for 20 min at 25 °C. The pellets were further washed with sterile saline and centrifuged at 100,000 g for 20 min. The resulting pellets were used as Sarkosyl-insoluble fraction. This study was approved by the research ethics committee of Tokyo Metropolitan Institute of Medical Science (number: 15–5), and carried out in accordance with the approved guidelines. Informed consent about the brain donation was obtained from all subjects.

### SDS-polyacrylamide gel electrophoresis (PAGE), immunoblotting and in-gel digestion

The Sarkosyl-insoluble, SDS/urea-soluble fractions were separated on 4 ~ 20% polyacrylamide gradient gels by SDS-PAGE and immunochemically detected as described^26^. Briefly, for immunoblotting, mAb anti-TDP-43 (60019-2-Ig, 1:3000, ProteinTech Group), polyclonal anti-TDP (10782-1-AP, 1:3000, ProteinTech Group) and pS409/410 (rabbit polyclonal, 1:1000) were used. Bands of TDP-43 and its derivatives were excised and soaked in 50 mM Tris-HCl, pH 8.0, containing 50% acetonitrile for 30 min. The gel was dried in a Speed-Vac (Savant) and incubated in 50 mM Tris-HCl, pH 8.0 containing 125–250 ng of modified trypsin (Roche Diagnostics, Mannheim, Germany) or chymotrypsin (Roche Diagnostics, Mannheim, Germany) at 37 °C for 6–20 hours. The digests were extracted from the gel twice with 100 μl of 0.1% TFA containing 60% acetonitrile. These two extracts were combined, evaporated in a Speed-Vac, and stored at −80 °C until assayed.

### Nano-flow liquid chromatography-ion trap mass spectrometry (LC-MS/MS)

The sample was resuspended in 0.1% formic acid containing 2% acetonitrile and introduced into a nano-flow HPLC system, DiNa fitted with an automatic sampler (KYA Technology Corporation, Tokyo, Japan). A packed nano-capillary column NTCC-360/75-3*-*123 (0.075 mm I.D. ×125 mm L, particle diameter 3 μm, Nikkyo Technos Co., Ltd., Tokyo, Japan) was used at a flow rate of 300 nl / min with a 2–80% linear gradient of acetonitrile for 60 min. Eluted peptides were directly detected with an ion trap mass spectrometer, Velos Pro (Thermo Fisher Scientific Inc., Waltham, USA) at a spray voltage of 1.9 kV and a collision energy of 35%. The mass acquisition method consisted of one full MS survey scan followed by an MS/MS scan of the most abundant precursor ions from the survey scan. Dynamic exclusion for the MS/MS was set to 30 seconds. An MS scan range of 400–2000 m/z was employed in the positive ion mode, followed by data-dependent MS/MS using the CID or HCD operating mode on the top 10 ions in order of abundance. The data were analyzed with Proteome Discoverer (Thermo Fisher Scientific Inc., Waltham, USA), Mascot software (Matrix Science Inc., Boston, USA) and Scaffold software (Proteome Software, Inc., Oregon, USA). Swiss prot and GenBank databases were used.

## Additional Information

**How to cite this article**: Kametani, F. *et al.* Mass spectrometric analysis of accumulated TDP-43 in amyotrophic lateral sclerosis brains. *Sci. Rep.* **6**, 23281; doi: 10.1038/srep23281 (2016).

## Supplementary Material

Supplementary Information

## Figures and Tables

**Table 1:** List of identifid peptides derived from accumulated TDP-43 in ALS patient case 1.

Query	Start	End	Observed	Mr(expt)	Mr(calc)	Delta	Score	Expect	Peptide
**fraction1**	**chymotrypsin**								
9373	212	226	871.6568	1741.299	1741.8386	−0.5395	91	5.40E-09	F.SQYGDVMDVFIPKPF.R
9389	212	226	872.741	1743.4674	1741.8386	1.6288	41	0.00021	F.SQYGDVMDVFIPKPF.R
9442	212	226	880.2186	1758.4227	1757.8335	0.5892	60	2.70E-05	F.SQYGDVMoDVFIPKPF.R
6387	215	226	683.1282	1364.2418	1363.6846	0.5572	23	0.0077	Y.GDVMDVFIPKPF.R
3616	235	243	520.0455	1038.0765	1039.4223	−1.3458	24	0.025	F.ADDQIAQSpL.C
4502	277	289	583.8335	1165.6524	1164.4949	1.1576	38	0.00028	F.GGNPGGFGNQGGF.G
12543	290	316	829.3413	2485.0021	2486.0452	−1.0431	45	0.0016	F.GNSRGGGAGLGN*NQGSNMGGGMNFGAF.S
	**trypsin**								
7855	84	95	641.5232	1281.0318	1280.5918	0.44	48	3.10E-05	R.KMDETDASSAVK.V
8748	103	114	671.5735	1341.1324	1340.7704	0.362	21	0.052	K.TSDLIVLGLPWK.T
3193	115	121	417.6076	833.2006	833.4131	−0.2124	26	0.0036	K.TTEQDLK.E
3198	115	121	417.6429	833.2713	833.4131	−0.1417	23	0.0071	K.TTEQDLK.E
6245	152	160	572.4623	1142.9101	1143.5448	−0.6347	20	0.014	R.FTEYETQVK.V
6249	152	160	572.5684	1143.1223	1143.5448	−0.4225	24	0.0057	R.FTEYETQVK.V
10549	215	227	513.2426	1536.7058	1535.7807	0.9252	26	0.025	Y.GDVMDVFIPKPFR
7515	252	263	626.6138	1251.2131	1250.6619	0.5512	36	0.0004	K.GISVHISNAEPK.H
7516	252	263	626.6459	1251.2772	1251.6459	−0.3687	33	0.0037	K.GISVHISN*AEPK.H
10016	276	291	742.3951	1482.7757	1482.6277	0.148	42	0.00011	R.FGGNPGGFGNQGGFGN.S
12266	276	293	863.7729	1725.5312	1725.7608	−0.2296	105	2.30E-10	R.FGGNPGGFGNQGGFGNSR.G
12269	276	293	864.0314	1726.0482	1725.7608	0.2874	103	2.30E-10	R.FGGNPGGFGNQGGFGNSR.G
12274	276	293	864.0852	1726.1559	1725.7608	0.3951	98	2.10E-09	R.FGGNPGGFGNQGGFGNSR.G
12276	276	293	864.1173	1726.2201	1725.7608	0.4592	97	2.30E-09	R.FGGNPGGFGNQGGFGNSR.G
12278	276	293	864.1233	1726.232	1725.7608	0.4712	101	3.40E-10	R.FGGNPGGFGNQGGFGNSR.G
12279	276	293	864.1337	1726.2528	1725.7608	0.492	111	8.90E-11	R.FGGNPGGFGNQGGFGNSR.G
12280	276	293	576.432	1726.274	1725.7608	0.5132	26	0.0082	R.FGGNPGGFGNQGGFGNSR.G
12290	276	293	576.6025	1726.7856	1725.7608	1.0248	36	0.0006	R.FGGNPGGFGNQGGFGNSR.G
12268	276	293	576.265	1725.7732	1726.7448	−0.9716	37	0.0007	R.FGGNPGGFGNQGGFGN*SR.G
12271	276	293	864.0555	1726.0964	1726.7448	−0.6484	90	1.30E-08	R.FGGNPGGFGN*QGGFGNSR.G
**fraction2**	**chymotrypsin**								
5089	176	188	742.6311	1483.2476	1483.7518	−0.5042	43	0.00092	C.KLPN*SKQSQDEPL.R
5096	176	188	743.321	1484.6275	1483.7518	0.8757	46	0.00041	C.KLPN*SKQSQDEPL.R
4168	211	221	654.5313	1307.0479	1306.554	0.4939	39	0.00027	F.FSQYGDVMDVF.I
6859	211	226	945.4351	1888.8556	1888.907	−0.0514	48	3.70E-05	F.FSQYGDVMDVFIPKPF.R
6861	211	226	946.2177	1890.4207	1889.891	0.5298	30	0.0016	F.FSQ*YGDVMDVFIPKPF.R
6880	211	226	953.7546	1905.4947	1904.9019	0.5928	52	4.10E-05	F.FSQYGDVMoDVFIPKPF.R
3531	212	221	580.9187	1159.8228	1159.4856	0.3373	35	5.70E-04	F.SQYGDVMDVF.I
6412	212	226	872.3367	1742.6589	1741.8386	0.8203	73	1.50E-07	F.SQYGDVMDVFIPKPF.R
6461	212	226	880.2487	1758.4828	1757.8335	0.6493	52	9.50E-05	F.SQYGDVMoDVFIPKPF.R
4522	215	226	682.6916	1363.3687	1363.6846	−0.3159	38	3.10E-04	Y.GDVMDVFIPKPF.R
4524	215	226	683.6007	1365.1869	1363.6846	1.5022	21	2.20E-02	Y.GDVMDVFIPKPF.R
6869	271	289	633.079	1896.2151	1897.8456	−1.6305	42	0.00049	L.ERSGRFGGNPGGFGN*QGGF.G
3544	277	289	583.3915	1164.7685	1164.4949	0.2737	28	0.0025	F.GGNPGGFGNQGGF.G
3546	277	289	583.4573	1164.9001	1164.4949	0.4053	27	0.0051	F.GGNPGGFGNQGGF.G
3548	277	289	583.528	1165.0413	1164.4949	0.5465	30	0.0014	F.GGNPGGFGNQGGF.G
7741	290	313	1106.3008	2210.587	2210.9182	−0.3312	95	1.50E-08	F.GNSRGGGAGLGNNQGSN*MGGGMNF.G
7743	290	313	737.9193	2210.7361	2210.9182	−0.1821	50	0.00044	F.GNSRGGGAGLGNNQGSN*MGGGMNF.G
7770	290	313	743.0682	2226.1827	2226.9131	−0.7304	61	2.20E-05	F.GNSRGGGAGLGN*NQGSNMoGGGMNF.G
7772	290	313	743.2908	2226.8505	2226.9131	−0.0626	38	0.0067	F.GNSRGGGAGLGN*NQGSNMGGGMoNF.G
7775	290	313	743.557	2227.6492	2226.9131	0.7361	48	0.00017	F.GNSRGGGAGLGNNQGSN*MGGGMoNF.G
7791	290	313	1121.8962	2241.7779	2241.924	−0.1461	65	7.30E-07	F.GNSRGGGAGLGNNQGSNMoGGGMoNF.G
7792	290	313	748.4241	2242.2504	2242.908	−0.6576	66	1.00E-05	F.GNSRGGGAGLGN*NQGSNMoGGGMoNF.G
7795	290	313	748.7297	2243.1672	2243.892	−0.7248	36	0.01	F.GN*SRGGGAGLGNNQ*GSNMoGGGMoNF.G
7796	290	313	748.7909	2243.3508	2243.892	−0.5412	47	0.00068	F.GNSRGGGAGLGN*NQGSN*MoGGGMoNF.G
7799	290	313	1123.2252	2244.4359	2243.892	0.5439	69	3.10E-07	F.GNSRGGGAGLGN*NQGSN*MoGGGMoNF.G
8563	290	316	829.6099	2485.8078	2486.0452	−0.2374	58	8.60E-05	F.GN*SRGGGAGLGNNQGSNMGGGMNFGAF.S
8567	290	316	830.2059	2487.5958	2487.0292	0.5666	67	1.20E-05	F.GNSRGGGAGLGN*NQGSN*MGGGMNFGAF.S
8603	290	316	834.7759	2501.3058	2503.0241	−1.7183	56	0.00012	F.GN*SRGGGAGLGNNQGSNMGGGMoN*FGAF.S
8606	290	316	835.1727	2502.4962	2503.0241	−0.5279	73	2.50E-06	F.GN*SRGGGAGLGNNQGSNMoGGGMN*FGAF.S
8611	290	316	835.5923	2503.755	2503.0241	0.7309	59	6.60E-05	F.GN*SRGGGAGLGNNQGSNMGGGMoN*FGAF.S
8667	290	316	840.1269	2517.3588	2518.035	−0.6762	61	4.90E-05	F.GNSRGGGAGLGNNQ*GSNMoGGGMoNFGAF.S
8671	290	316	840.6654	2518.9744	2519.019	−0.0446	64	2.20E-05	F.GN*SRGGGAGLGNNQGSNMoGGGMoN*FGAF.S
1915	317	323	779.2965	778.2892	778.3353	−0.0462	37	0.00039	F.SINPAMoM.A
1916	317	323	779.4086	778.4013	778.3353	0.066	37	0.00038	F.SINPAMoM.A
4592	317	330	688.1877	1374.3609	1374.6635	−0.3026	28	0.0041	F.SINPAMMoAAAQAAL.Q
4704	317	330	696.6204	1391.2262	1391.6424	−0.4163	48	0.00028	F.SINPAMoMoAAAQ*AAL.Q
5282	317	331	760.6963	1519.378	1518.717	0.661	62	1.30E-05	F.SINPAMoMoAAAQAALQ.S
6767	317	334	616.6206	1846.84	1846.8705	−0.0305	21	0.019	F.SINPAMMAAAQAALQSSW.G
6768	317	334	924.6777	1847.3409	1846.8705	0.4704	43	9.10E-05	F.SINPAMMAAAQAALQSSW.G
6835	317	334	941.1243	1880.234	1879.8444	0.3896	33	0.014	F.SINPAMoMoAAAQ*AALQSSW.G
3280	324	334	552.4872	1102.9598	1102.5407	0.4191	68	7.20E-07	M.AAAQAALQSSW.G
7465	340	359	1037.6927	2073.3709	2072.893	0.478	38	0.00028	M.LASQQNQSGPSGNNQNQGNM.Q
10098	340	367	1015.7645	3044.2718	3044.3391	−0.0673	68	1.60E-05	M.LASQQNQSGPSGNNQNQGN*MQREPNQAF.G
10096	340	367	1015.7145	3044.1216	3045.3231	−1.2014	60	8.90E-05	M.LASQQNQSGPSGNNQN*Q*GNMQREPNQAF.G
10136	340	367	1020.859	3059.5552	3060.334	−0.7788	75	9.60E-07	M.LASQQNQSGPSGNNQNQ*GNMoQREPNQAF.G
10145	340	367	1021.6365	3061.8876	3061.318	0.5696	57	0.00017	M.LASQQNQSGPSGNNQ*N*QGNMoQREPNQAF.G
10150	340	367	1021.8416	3062.5028	3062.302	0.2008	45	0.0027	M.LASQQNQSGPSGN*N*Q*NQGNMoQREPNQAF.G
9721	341	367	978.223	2931.6472	2931.255	0.3922	50	0.00076	L.ASQQNQSGPSGNNQNQGN*MQREPNQAF.G
9722	341	367	978.352	2932.0343	2931.255	0.7793	48	0.0014	L.ASQQNQSGPSGNNQN*QGNMQREPNQAF.G
9763	341	367	983.592	2947.7543	2947.2499	0.5044	34	0.012	L.ASQQNQSGPSGNNQNQGNMoQREPNQ*AF.G
9764	341	367	983.6382	2947.8927	2947.2499	0.6428	40	0.0091	L.ASQQNQSGPSGNNQNQGNMoQREPNQ*AF.G
9765	341	367	983.6984	2948.0733	2947.2499	0.8233	50	9.70E-05	L.ASQQNQSGPSGNNQNQGNMoQREPN*QAF.G
9772	341	367	983.9515	2948.8328	2948.2339	0.5989	37	0.018	L.ASQQNQ*SGPSGN*NQNQGNMoQREPNQAF.G
6163	389	405	561.7853	1682.334	1680.5164	1.8176	20	0.034	A.SpNAGSpGSGFN*GGFGSSM.D
2063	398	405	426.5261	851.0376	851.2521	−0.2145	13	0.063	F.NGGFGSpSMo.D
2064	398	405	426.5549	851.0953	851.2521	−0.1568	13	0.057	F.NGGFGSpSMo.D
	**trypsin**								
4541	252	263	626.1067	1250.1988	1250.6619	−0.4631	47	3.70E-05	K.GISVHISNAEPK.H
4554	252	263	626.5655	1251.1164	1251.6459	−0.5295	30	0.0069	K.GISVHISN*AEPK.H
10298	273	293	676.4501	2026.3284	2026.8994	−0.571	49	3.20E-05	R.SGRFGGN*PGGFGNQGGFGNSR.G
8412	276	293	863.5454	1725.0763	1725.7608	−0.6845	68	4.20E-07	R.FGGNPGGFGNQGGFGNSR.G
8413	276	293	863.6377	1725.2608	1725.7608	−0.5	74	1.00E-07	R.FGGNPGGFGNQGGFGNSR.G
8414	276	293	863.6544	1725.2943	1725.7608	−0.4665	81	2.80E-08	R.FGGNPGGFGNQGGFGNSR.G
8415	276	293	863.7958	1725.577	1725.7608	−0.1838	96	4.00E-09	R.FGGNPGGFGNQGGFGNSR.G
8421	276	293	864.0331	1726.0516	1725.7608	0.2908	90	1.30E-08	R.FGGNPGGFGNQGGFGNSR.G
8424	276	293	864.0687	1726.1228	1725.7608	0.3619	82	2.10E-08	R.FGGNPGGFGNQGGFGNSR.G
8425	276	293	864.0773	1726.14	1725.7608	0.3792	102	8.60E-10	R.FGGNPGGFGNQGGFGNSR.G
8426	276	293	864.0829	1726.1512	1725.7608	0.3904	112	8.80E-11	R.FGGNPGGFGNQGGFGNSR.G
8427	276	293	864.0865	1726.1585	1725.7608	0.3977	83	1.60E-08	R.FGGNPGGFGNQGGFGNSR.G
8428	276	293	864.1036	1726.1926	1725.7608	0.4318	74	1.10E-07	R.FGGNPGGFGNQGGFGNSR.G
8429	276	293	864.1146	1726.2146	1725.7608	0.4537	103	5.60E-10	R.FGGNPGGFGNQGGFGNSR.G
8416	276	293	863.8204	1725.6263	1726.7448	−1.1185	87	2.10E-08	R.FGGNPGGFGN*QGGFGNSR.G
8417	276	293	863.9819	1725.9493	1726.7448	−0.7955	88	2.50E-08	R.FGGN*PGGFGNQGGFGNSR.G
8419	276	293	863.9979	1725.9813	1726.7448	−0.7635	90	1.30E-08	R.FGGNPGGFGN*QGGFGNSR.G
8420	276	293	864.0292	1726.0438	1726.7448	−0.701	81	2.40E-08	R.FGGN*PGGFGNQGGFGNSR.G
8423	276	293	864.066	1726.1175	1726.7448	−0.6273	69	1.70E-06	R.FGGN*PGGFGNQGGFGNSR.G
8430	276	293	864.1177	1726.2209	1726.7448	−0.5239	90	1.20E-08	R.FGGN*PGGFGNQGGFGNSR.G
8434	276	293	864.2111	1726.4077	1726.7448	−0.3371	96	3.30E-09	R.FGGNPGGFGN*QGGFGNSR.G
8435	276	293	864.2555	1726.4964	1726.7448	−0.2484	95	4.30E-09	R.FGGNPGGFGN*QGGFGNSR.G
8436	276	293	576.5455	1726.6148	1726.7448	−0.1301	31	0.012	R.FGGNPGGFGNQGGFGN*SR.G
8438	276	293	864.4259	1726.8372	1726.7448	0.0924	82	1.00E-07	R.FGGN*PGGFGNQGGFGNSR.G
8439	276	293	864.4338	1726.853	1726.7448	0.1082	82	1.00E-07	R.FGGNPGGFGN*QGGFGNSR.G
8440	276	293	864.4481	1726.8817	1726.7448	0.1369	77	3.30E-07	R.FGGNPGGFGN*QGGFGNSR.G
8441	276	293	864.4512	1726.8878	1726.7448	0.143	72	9.10E-07	R.FGGNPGGFGNQ*GGFGNSR.G
8443	276	293	864.5175	1727.0205	1726.7448	0.2757	90	3.90E-09	R.FGGNPGGFGN*QGGFGNSR.G
8444	276	293	576.6903	1727.0491	1726.7448	0.3043	40	0.00049	R.FGGNPGGFGNQGGFGN*SR.G
8445	276	293	864.5552	1727.0959	1726.7448	0.3511	78	5.70E-08	R.FGGNPGGFGN*QGGFGNSR.G
8447	276	293	864.6735	1727.3324	1726.7448	0.5875	78	1.50E-07	R.FGGN*PGGFGNQGGFGNSR.G
8448	276	293	864.687	1727.3595	1726.7448	0.6146	81	2.60E-08	R.FGGN*PGGFGNQGGFGNSR.G
8446	276	293	864.6155	1727.2164	1727.7288	−0.5124	79	1.50E-07	R.FGGN*PGGFGNQ*GGFGNSR.G
8458	276	293	865.0176	1728.0206	1727.7288	0.2918	53	1.20E-05	R.FGGN*PGGFGNQGGFGN*SR.G
8463	276	293	865.0424	1728.0703	1727.7288	0.3415	39	0.00023	R.FGGN*PGGFGNQ*GGFGNSR.G
8470	276	293	865.0927	1728.1707	1727.7288	0.4419	50	2.10E-05	R.FGGN*PGGFGNQGGFGN*SR.G
8508	276	293	865.5012	1728.9879	1728.7128	0.275	32	0.0016	R.FGGN*PGGFGN*Q*GGFGNSR.G
5555	280	293	676.4501	1350.8856	1350.6065	0.2791	75	1.00E-07	N.PGGFGNQGGFGNSR.G
**fraction3**	**chymotrypsin**								
9282	211	226	945.6774	1889.3402	1888.907	0.4332	32	0.001	F.FSQYGDVMDVFIPKPF.R
8489	212	226	872.2216	1742.4286	1741.8386	0.59	68	4.10E-07	F.SQYGDVMDVFIPKPF.R
8572	212	226	879.9073	1757.8001	1757.8335	−0.0333	33	0.0012	F.SQYGDVMoDVFIPKPF.R
4043	277	289	583.3818	1164.7491	1164.4949	0.2543	35	0.00048	F.GGNPGGFGNQGGF.G
4044	277	289	583.4044	1164.7942	1164.4949	0.2993	27	0.0032	F.GGNPGGFGNQGGF.G
4048	277	289	583.4793	1164.9441	1164.4949	0.4492	37	0.00059	F.GGNPGGFGNQGGF.G
4057	277	289	583.5714	1165.1283	1164.4949	0.6334	20	0.014	F.GGNPGGFGNQGGF.G
10527	290	313	743.5016	2227.4829	2225.9291	1.5539	32	0.0086	F.GNSRGGGAGLGNNQGSNMoGGGMNF.G
10566	290	313	1122.9318	2243.849	2243.892	−0.043	27	0.021	F.GNSRGGGAGLGN*NQ*GSNMoGGGMoNF.G
11297	290	316	840.3088	2517.9047	2518.035	−0.1303	38	0.0081	F.GNSRGGGAGLGN*NQGSNMoGGGMoNFGAF.S
11299	290	316	840.5651	2518.6734	2518.035	0.6383	30	0.0058	F.GN*SRGGGAGLGNNQGSNMoGGGMoNFGAF.S
7796	298	313	546.0648	1635.1726	1636.5535	−1.3808	24	0.082	A.GLGN*NQ*GSpN*MGGGMNF.G
5837	317	330	688.5671	1375.1196	1374.6635	0.4561	25	0.0073	F.SINPAMMAAAQAAL.Q
3589	324	334	552.6029	1103.1912	1102.5407	0.6506	36	0.0017	M.AAAQAALQSSW.G
1956	398	405	426.5643	851.1141	852.2361	−1.122	23	0.038	F.N*GGFGSpSMo.D
	**trypsin**								
6817	103	114	671.6575	1341.3004	1340.7704	0.53	61	2.90E-06	K.TSDLIVLGLPWK
4663	152	160	572.6335	1143.2524	1143.5448	−0.2924	41	0.00021	R.FTEYETQVK.V
4677	152	160	573.5289	1145.0432	1143.5448	1.4984	33	0.00079	R.FTEYETQVK.V
5751	252	263	626.5578	1251.101	1251.6459	−0.5448	33	0.0017	K.GISVHISN*AEPK.H
11145	276	293	864.0863	1726.158	1725.7608	0.3972	104	9.80E-10	R.FGGNPGGFGNQGGFGNSR.G
11147	276	293	576.6158	1726.8257	1725.7608	1.0649	43	0.00028	R.FGGNPGGFGNQGGFGNSR.G
11138	276	293	864.0644	1726.1142	1726.7448	−0.6306	99	3.00E-09	R.FGGN*PGGFGNQGGFGNSR.G
11140	276	293	864.1228	1726.231	1726.7448	−0.5138	92	1.60E-08	R.FGGNPGGFGNQGGFGN*SR.G
11142	276	293	864.1336	1726.2527	1726.7448	−0.4922	85	1.60E-08	R.FGGNPGGFGN*QGGFGNSR.G
11144	276	293	864.2585	1726.5024	1726.7448	−0.2424	25	0.052	R.FGGN*PGGFGNQGGFGNSR.G
11157	276	293	576.9172	1727.7297	1726.7448	0.9849	59	3.00E-05	R.FGGNPGGFGNQGGFGN*SR.G
**fraction4**	**chymotrypsin**								
4202	153	162	622.3894	1242.7642	1242.5802	0.184	40	0.00024	F.TEYETQVKVMo.S
9557	290	313	748.4747	2242.4024	2242.908	−0.5056	65	1.20E-05	F.GN*SRGGGAGLGNNQGSNMoGGGMoNF.G
	**trypsin**								
15817	56	79	875.6911	2624.0515	2623.3799	0.6716	30	0.0048	R.LVEGILHAPDAGWGNLVYVVNYPK.D
15818	56	79	875.9263	2624.757	2623.3799	1.3771	48	0.00018	R.LVEGILHAPDAGWGNLVYVVNYPK.D
15822	56	79	876.7198	2627.1377	2625.3479	1.7899	40	0.003	R.LVEGILHAPDAGWGN*LVYVVN*YPK.D
5505	84	95	641.2124	1280.4102	1280.5918	−0.1816	35	0.0015	R.KMDETDASSAVK.V
5513	84	95	641.6223	1281.2301	1280.5918	0.6383	64	1.20E-06	R.KMDETDASSAVK.V
5516	84	95	428.1922	1281.5549	1280.5918	0.963	30	0.0056	R.KMDETDASSAVK.V
5728	84	95	649.5043	1296.9941	1296.5867	0.4074	51	1.60E-05	R.KMoDETDASSAVK.V
4136	85	95	576.8164	1151.6183	1152.4969	−0.8786	32	0.0011	K.MDETDASSAVK.V
4140	85	95	576.885	1151.7555	1152.4969	−0.7414	20	0.013	K.MDETDASSAVK.V
4159	85	95	577.0442	1152.0738	1152.4969	−0.423	22	0.008	K.MDETDASSAVK.V
4161	85	95	577.0544	1152.0943	1152.4969	−0.4025	24	0.014	K.MDETDASSAVK.V
4173	85	95	577.1104	1152.2063	1152.4969	−0.2906	50	2.10E-05	K.MDETDASSAVK.V
4196	85	95	577.2958	1152.577	1152.4969	0.0802	20	0.053	K.MDETDASSAVK.V
4204	85	95	577.8925	1153.7704	1152.4969	1.2735	26	0.0035	K.MDETDASSAVK.V
6390	103	114	671.2714	1340.5282	1340.7704	−0.2422	54	1.80E-05	K.TSDLIVLGLPWK.T
4096	152	160	572.927	1143.8394	1143.5448	0.2946	39	0.00021	R.FTEYETQVK.V
4097	152	160	572.9576	1143.9006	1143.5448	0.3558	28	0.0032	R.FTEYETQVK.V
4100	152	160	573.1072	1144.1998	1143.5448	0.655	31	0.0023	R.FTEYETQVK.V
5209	252	263	626.5698	1251.1251	1250.6619	0.4632	34	0.00061	K.GISVHISNAEPK.H
10703	276	293	864.0738	1726.1331	1725.7608	0.3723	108	2.10E-10	R.FGGNPGGFGNQGGFGNSR.G
10689	276	293	576.141	1725.4011	1726.7448	−1.3437	45	0.00089	R.FGGNPGGFG*NQGGFGNSR.G
10714	276	293	864.5613	1727.1081	1727.7288	−0.6207	103	1.20E-09	R.FGGN*PGGFGNQGGFGN*SR.G
**fraction5**	**chymotrypsin**								
3922	114	123	418.5765	1252.7075	1253.6139	−0.9064	38	0.0024	W.KTTEQDLKEY.F
5597	300	313	489.2062	1464.5969	1466.4479	−1.851	24	0.061	L.GN*N*QGS*N*MGGGMNF.G
1088	330	336	413.0149	824.0152	823.3534	0.6618	22	0.018	A.LQSSWGMo.M
8438	386	405	658.5479	1972.6219	1974.5894	−1.9675	32	0.014	W.GSASNAGSGSpGFNGGFGSpSpM.D
	**trypsin**								
15330	56	79	875.8154	2624.4245	2623.3799	1.0446	40	0.00088	R.LVEGILHAPDAGWGNLVYVVNYPK.D
15331	56	79	875.8346	2624.4819	2623.3799	1.1021	34	0.0077	R.LVEGILHAPDAGWGNLVYVVNYPK.D
5230	84	95	641.4845	1280.9544	1280.5918	0.3626	56	2.70E-05	R.KMDETDASSAVK.V
5232	84	95	641.5112	1281.0078	1280.5918	0.416	51	4.20E-05	R.KMDETDASSAVK.V
3945	85	95	576.9318	1151.8491	1152.4969	−0.6478	44	0.00018	K.MDETDASSAVK.V
3952	85	95	577.0027	1151.9908	1152.4969	−0.506	26	0.0054	K.MDETDASSAVK.V
3958	85	95	577.0422	1152.0699	1152.4969	−0.4269	50	1.90E-05	K.MDETDASSAVK.V
3980	85	95	577.1116	1152.2086	1152.4969	−0.2883	31	0.004	K.MDETDASSAVK.V
3983	85	95	577.1158	1152.217	1152.4969	−0.2798	18	0.021	K.MDETDASSAVK.V
3990	85	95	577.1333	1152.252	1152.4969	−0.2448	23	0.057	K.MDETDASSAVK.V
4006	85	95	577.1882	1152.3619	1152.4969	−0.1349	25	0.0043	K.MDETDASSAVK.V
4007	85	95	577.1957	1152.3768	1152.4969	−0.12	30	0.0024	K.MDETDASSAVK.V
4013	85	95	577.2638	1152.513	1152.4969	0.0162	44	7.80E-05	K.MDETDASSAVK.V
4018	85	95	577.4311	1152.8476	1152.4969	0.3508	35	0.0031	K.MDETDASSAVK.V
4021	85	95	577.4545	1152.8944	1152.4969	0.3975	52	2.40E-05	K.MDETDASSAVK.V
4030	85	95	578.0698	1154.125	1152.4969	1.6281	33	0.0017	K.MDETDASSAVK.V
4093	85	95	585.0585	1168.1024	1168.4918	−0.3894	27	0.0031	K.MoDETDASSAVK.V
4097	85	95	585.7054	1169.3962	1168.4918	0.9044	24	0.0053	K.MoDETDASSAVK.V
6018	103	114	671.1033	1340.192	1340.7704	−0.5784	37	0.00067	K.TSDLIVLGLPWK.T
3868	152	160	573.0732	1144.1318	1143.5448	0.587	30	0.0028	R.FTEYETQVK.V
2331	182	189	486.4414	970.8682	971.4672	−0.599	25	0.031	K.QSQDEPLR.S
2334	182	189	486.5248	971.035	971.4672	−0.4322	25	0.017	K.QSQDEPLR.S
2345	182	189	487.3027	972.5909	971.4672	1.1237	26	0.013	K.QSQDEPLR.S
10280	276	293	864.1884	1726.3621	1725.7608	0.6013	106	3.40E-10	R.FGGNPGGFGNQGGFGNSR.G
10284	276	293	576.4902	1726.4489	1726.7448	−0.296	29	0.028	R.FGGNPGGFGNQ*GGFGNSR.G
10291	276	293	864.5842	1727.1539	1726.7448	0.4091	90	2.20E-08	R.FGGNPGGFGNQGGFGN*SR.G
**fravtion6**	**chymotrypsin**								
3105	277	289	583.4806	1164.9467	1164.4949	0.4519	25	0.0045	F.GGNPGGFGNQGGF.G
8141	298	315	594.347	1780.0191	1778.6389	1.3802	32	0.015	A.GLGNNQGSpN*MoGGGMNFGA.F
7563	300	316	565.4505	1693.3297	1693.5985	−0.2688	29	0.031	L.GN*N*QGSNMoGGGMoN*FGAF.S
5625	317	330	486.4602	1456.3588	1455.6138	0.7449	18	0.041	F.SpIN*PAMoMAAAQAAL.Q
	**trypsin**								
4254	103	114	671.3554	1340.6963	1340.7704	−0.0741	36	0.00083	K.TSDLIVLGLPWK.T
7335	276	293	864.0093	1726.004	1725.7608	0.2432	65	2.40E-06	R.FGGNPGGFGNQGGFGNSR.G
7340	276	293	864.1556	1726.2966	1726.7448	−0.4482	87	4.90E-08	R.FGGNPGGFGN*QGGFGNSR.G
**fraction7**	**chymotrypsin**								
4017	277	289	583.4226	1164.8307	1164.4949	0.3358	32	0.001	F.GGNPGGFGNQGGF.G
8195	375	391	579.1056	1734.2949	1733.5372	0.7577	20	0.095	Y.SpGSN*SGAAIGWGSpASpNA.G
6844	389	404	516.8932	1547.6579	1548.492	−0.834	23	0.065	A.SpNAGSGSGFNGGFGSSp.M
9620	389	409	673.531	2017.5712	2017.7361	−0.1649	20	0.078	A.SN*AGSGSGFNGGFGSSpMDSKS.S
	**trypsin**								
7417	103	114	671.6024	1341.1903	1340.7704	0.4199	39	0.00022	K.TSDLIVLGLPWK.T
10947	276	293	864.018	1726.0215	1725.7608	0.2606	108	3.50E-10	R.FGGNPGGFGNQGGFGNSR.G
**fraction8**	**chymotrypsin**								
			not detected						
	**trypsin**								
4254	103	114	671.3554	1340.6963	1340.7704	−0.0741	36	0.00083	K.TSDLIVLGLPWK.T
7335	276	293	864.0093	1726.004	1725.7608	0.2432	65	2.40E-06	R.FGGNPGGFGNQGGFGNSR.G
7340	276	293	864.1556	1726.2966	1726.7448	−0.4482	87	4.90E-08	R.FGGNPGGFGN*QGGFGNSR.G

p indicates phosphorylation site; o indicates oxidation site; * indicates deamidation site.

**Table 2:** List of identifid peptides derived from accumulated TDP-43 in ALS patient case 2.

Query	Start	End	Observed	Mr(expt)	Mr(calc)	Delta	Score	Expect	Peptide
**fraction1**	**chymotrypsin**								
4855	215	226	683.3186	1364.6226	1363.6846	0.938	17	0.066	Y.GDVMDVFIPKPF.R
20958	290	313	738.3618	2212.0636	2210.9182	1.1455	28	0.0071	F.GNSRGGGAGLGNNQ*GSNMGGGMNF.G
21027	300	321	742.2366	2223.6879	2222.8398	0.8481	27	0.048	L.GNNQGSpN*MGGGMN*FGAFSINPA.M
21271	316	336	771.2789	2310.815	2311.92	−1.1051	29	0.064	A.FSIN*PAMoMoAAAQAALQSSpWGMo.M
17965	322	336	556.6434	1666.9085	1666.6078	0.3007	26	0.056	A.MMoAAAQ*AALQ*SSpWGMo.M
10760	389	398	489.2573	976.5	978.2968	−1.7968	23	0.057	A.SpN*AGSGSGFN*.G
10762	389	398	489.3521	976.6897	978.2968	−1.6071	22	0.064	A.SpN*AGSGSGFN*.G
	**trypsin**								
24329	228	251	898.5277	2692.5613	2694.2652	−1.7039	38	0.0016	R.AFAFVTFADDQIAQS þ LCGEDLIIK.G
12874	252	263	627.0836	1252.1527	1250.6619	1.4908	46	7.90E-05	K.GISVHISNAEPK.H
19990	276	293	863.7753	1725.5361	1725.7608	−0.2247	103	1.50E-09	R.FGGNPGGFGNQGGFGNSR.G
20016	276	293	864.1752	1726.3359	1725.7608	0.5751	121	5.20E-12	R.FGGNPGGFGNQGGFGNSR.G
20030	276	293	864.5692	1727.1239	1725.7608	1.3631	100	1.30E-09	R.FGGNPGGFGNQGGFGNSR.G
**fraction2**	**chymotrypsin**								
14560	76	85	725.5255	1449.0365	1448.7194	0.3171	23	0.0065	V.NYPKgDNKaRKM.D
18417	290	311	684.6705	2050.9898	2049.7041	1.2857	22	0.096	F.GN*SpRGGGAGLGN*N*Q*GSN*MoGGGM.N
15143	382	397	502.4551	1504.3433	1504.5984	−0.2551	18	0.065	A.AIGWGSASNAGSpGSGF.N
17655	389	405	615.114	1842.3202	1840.4491	1.8711	30	0.024	A.SpN*AGSpGSpGFNGGFGSSpM.D
12347	398	408	421.6983	1262.0732	1261.3723	0.7008	23	0.078	F.NGGFGSSpMoDSpK.S
14828	398	411	738.5961	1475.1776	1476.463	−1.2854	17	0.045	F.NGGFGS.pSMDSKSpSG.W
	**trypsin**								
9063	76	82	439.4402	876.8658	877.4294	−0.5635	21	0.065	V.NYPKDNK.R
12273	152	160	572.6329	1143.2513	1143.5448	−0.2935	24	0.0066	R.FTEYETQVK.V
13442	252	263	626.5903	1251.166	1250.6619	0.5041	60	2.20E-06	K.GISVHISNAEPK.H
18848	276	293	864.1686	1726.3226	1726.7448	−0.4222	100	2.80E-09	R.FGGNPGGFGNQGGFGN*SR.G
18879	276	293	865.5833	1729.1519	1727.7288	1.4231	92	2.30E-09	R.FGGNPGGFGN*QGGFGN*SR.G
14839	280	293	676.5123	1351.0101	1350.6065	0.4036	75	9.00E-08	N.PGGFGNQGGFGNSR.G
14842	280	293	676.5812	1351.1478	1350.6065	0.5413	46	0.00033	N.PGGFGNQGGFGNSR.G
14844	280	293	676.6381	1351.2617	1350.6065	0.6552	64	2.80E-06	N.PGGFGNQGGFGNSR.G
14837	280	293	676.4991	1350.9837	1351.5905	−0.6068	33	0.0068	N.PGGFGN*QGGFGNSR.G
**fraction3**	**chymotrypsin**								
19885	212	226	880.3099	1758.6053	1757.8335	0.7718	22	0.0093	F.SQYGDVMoDVFIPKPF.R
15096	215	226	683.6068	1365.1989	1363.6846	1.5143	34	0.00061	Y.GDVMDVFIPKPF.R
4235	230	234	584.3881	583.3808	583.3006	0.0802	26	0.0086	F.AFVTF.A
4246	230	234	584.5333	583.5261	583.3006	0.2254	26	0.0084	F.AFVTF.A
18976	290	307	831.7477	1661.4809	1662.7128	−1.2319	40	0.00047	F.GNSRGGGAGLGNNQGSNMo.G
21767	290	313	748.6056	2242.7949	2242.908	−0.1131	36	0.0087	F.GNSRGGGAGLGNNQGSNMoGGGMoNF.G
20036	298	315	594.1538	1779.4396	1780.607	−1.1674	26	0.065	A.GLGNN*Q*GSpN*MoGGGMNFGA.F
19286	300	316	564.2299	1689.6679	1691.6304	−1.9625	23	0.057	L.GNNQGSNMoGGGMoN*FGAF.S
19330	300	316	565.4157	1693.2251	1691.6304	1.5947	22	0.032	L.GNNQGSNMoGGGMoN*FGAF.S
21702	300	321	741.259	2220.7553	2220.8718	−0.1165	20	0.068	L.GNNQGSpNMGGGMNFGAFSINPA.M
21046	341	359	654.4437	1960.3094	1960.7929	−0.4836	58	3.10E-05	L.ASQQNQSGPSGNNQNQ*GNM.Q
20839	386	405	633.3611	1897.0616	1895.6071	1.4546	27	0.062	W.GSASpN*AGSpGSGFNGGFGSSM.D
21630	389	411	727.5361	2179.5866	2177.7845	1.8021	32	0.027	A.SNAGSpGSGFN*GGFGSSMoDSKSSG.W
	**trypsin**								
21995	209	227	774.8737	2321.5992	2321.1191	0.4801	39	0.0013	R.EFFSQYGDVMDVFIPKPFR
22343	228	251	898.5321	2692.5745	2694.2652	−1.6907	45	0.0003	R.AFAFVTFADDQIAQSpLCGEDLIIK.G
13384	252	263	625.472	1248.9294	1250.6619	−1.7325	27	0.0032	K.GISVHISNAEPK.H
13416	252	263	626.5458	1251.077	1250.6619	0.4151	28	0.0078	K.GISVHISNAEPK.H
13418	252	263	626.6263	1251.238	1250.6619	0.5761	48	6.10E-05	K.GISVHISNAEPK.H
19580	276	293	864.1287	1726.2429	1725.7608	0.4821	102	5.50E-10	R.FGGNPGGFGNQGGFGNSR.G
19582	276	293	864.1488	1726.283	1725.7608	0.5222	122	6.00E-12	R.FGGNPGGFGNQGGFGNSR.G
19600	276	293	865.1992	1728.3839	1728.7128	−0.329	28	0.036	R.FGGN*PGGFGNQ*GGFGN*SR.G
19637	276	293	866.3511	1730.6877	1728.7128	1.9749	75	9.20E-08	R.FGGN*PGGFGN*QGGFGN*SR.G
**fraction4**	**chymotrypsin**								
15701	215	226	683.5077	1365.0008	1363.6846	1.3162	34	0.0018	Y.GDVMDVFIPKPF.R
15928	317	330	689.0359	1376.0573	1375.6475	0.4098	27	0.0096	F.SIN*PAMMoAAAQAAL.Q
21325	324	339	581.5296	1741.567	1739.6242	1.9428	19	0.085	M.AAAQAALQ*SSpWGMoMoGMo.L
	**trypsin**								
14177	252	263	627.0461	1252.0777	1251.6459	0.4318	35	0.0051	K.GISVHISN*AEPK.H
23138	273	293	676.4238	2026.2497	2025.9154	0.3342	47	0.00016	R.SGRFGGNPGGFGNQGGFGNSR.G
20999	276	293	863.697	1725.3794	1725.7608	−0.3815	105	8.20E-10	R.FGGNPGGFGNQGGFGNSR.G
21025	276	293	864.4716	1726.9286	1725.7608	1.1678	104	2.90E-10	R.FGGNPGGFGNQGGFGNSR.G
**fraction5**	**chymotrypsin**								
7104	109	113	585.5835	584.5762	584.3322	0.244	26	0.0077	V.LGLPW.K
11793	131	139	537.3479	1072.6812	1072.6314	0.0498	31	0.011	V.LMVQVKKDL.K
7071	230	234	583.6779	582.6706	583.3006	−0.63	26	0.0074	F.AFVTF.A
7086	230	234	584.4906	583.4833	583.3006	0.1827	26	0.0072	F.AFVTF.A
12944	277	289	583.3265	1164.6384	1164.4949	0.1435	27	0.0028	F.GGNPGGFGNQGGF.G
15760	298	311	696.9571	1391.8996	1391.437	0.4626	25	0.055	A.GLGNN*Q*GSpN*MGGGM.N
20561	298	316	649.7761	1946.3065	1944.6543	1.6522	25	0.051	A.GLGN*N*Q*GSpN*MoGGGMoNFGAF.S
22061	314	337	863.6423	2587.905	2586.03	1.875	33	0.0056	F.GAFSpINPAMoMoAAAQ*AALQSSWGMoMo.G
12943	375	385	583.3155	1164.6164	1165.3842	−0.7678	19	0.078	Y.SpGSNSpGAAIGW.G
22006	375	400	845.8151	2534.4234	2532.8023	1.6211	27	0.061	Y.SGSNSpGAAIGWGSpASpNAGSpGSGFNGG.F
11997	389	400	546.7824	1091.5503	1092.3397	−0.7894	22	0.088	A.SN*AGSpGSGFN*GG.F
	**trypsin**								
12956	152	160	572.6786	1143.3427	1143.5448	−0.2021	36	0.0034	R.FTEYETQVK.V
21351	209	227	775.3962	2323.1669	2322.1031	1.0638	17	0.069	R.EFFSQ*YGDVMDVFIPKPFR.A
14102	252	263	626.5144	1251.0142	1250.6619	0.3524	39	0.00022	K.GISVHISNAEPK.H
18594	276	293	863.6197	1725.2249	1725.7608	−0.5359	107	1.10E-10	R.FGGNPGGFGNQGGFGNSR.G
**fraction6**	**chymotrypsin**								
23141	290	321	1054.8215	3161.4428	3162.1846	−0.7418	22	8.20E-02	F.GNSpRGGGAGLGN*N*Q*GSNMoGGGMoNFGAFSpINPA.M
22697	297	322	897.8206	2690.4399	2689.0162	1.4237	23	5.50E-02	G.AGLGNNQGSpNMGGGMNFGAFSpINPAMo.M
19240	300	318	659.1209	1974.3407	1972.6969	1.6439	28	2.10E-02	L.GNNQ*GSpN*MoGGGMoNFGAFSI.N
22742	316	340	905.2916	2712.8529	2712.1167	0.7362	29	7.80E-02	A.FSINPAMMAAAQ*AALQ*SpSWGMoMGML.A
13426	317	330	481.3466	1441.0179	1439.6189	1.3989	21	4.30E-02	F.SpINPAMMAAAQ*AAL.Q
10747	330	340	425.4213	1273.2422	1272.5189	0.7233	26	3.30E-02	A.LQ*SSWGMMoGMoL.A
16711	388	404	567.5427	1699.6063	1700.4794	−0.8731	18	9.80E-02	S.ASpN*AGSGSGFNGGFGSpSp.M
	**trypsin**								
8983	182	189	486.6083	971.2021	971.4672	−0.2651	36	2.70E-03	K.QSQDEPLR.S
12216	252	263	626.5559	1251.0973	1250.6619	0.4354	57	4.20E-06	K.GISVHISNAEPK.H
17819	276	293	863.6684	1725.3222	1725.7608	−0.4386	123	7.00E-12	R.FGGNPGGFGNQGGFGNSR.G
**fraction7**	**chymotrypsin**								
14499	56	68	689.5842	1377.1539	1376.7088	0.4451	56	9.80E-06	R.LVEGILHAPDAGW.G
18452	56	71	831.8374	1661.6602	1660.8573	0.8029	40	0.0006	R.LVEGILHAPDAGWGNL.V
3963	230	234	584.48	583.4727	583.3006	0.1721	26	0.0072	F.AFVTF.A
15174	368	381	474.0898	1419.2475	1418.4024	0.8451	33	0.0091	F.GSpGNN*SpYSGSNSGA.A
	**trypsin**								
21694	56	79	876.0681	2625.1825	2624.3639	0.8186	61	7.10E-06	R.LVEGILHAPDAGWGN*LVYVVNYPK.D
10852	84	95	641.5242	1281.0338	1280.5918	0.442	70	1.10E-06	R.KMDETDASSAVK.V
11727	103	114	671.4896	1340.9647	1340.7704	0.1943	38	0.00025	K.TSDLIVLGLPWK.T
17298	122	136	889.2975	1776.5804	1775.8804	0.7	69	1.90E-06	K.EYFSTFGEVLMVQVK.K
8866	152	160	573.5646	1145.1147	1143.5448	1.5699	26	0.0039	R.FTEYETQVK.V
10398	252	263	626.6704	1251.3263	1250.6619	0.6644	37	0.0007	K.GISVHISNAEPK.H

p indicates phosphorylation site; o indicates oxidation site; * indicates deamidation site; a indicates acetylation site; g indicates ubiqutination site.

**Table 3:** N-terminal sequences of abnormally accumulated TDP-43 fragments in human brain.

Disease	Molecular weight	Sequence	Reference
ALS	45kDa	56-LVEGILHA--	this study
	43kDa	56-LVEGILHA--	
	30–35kDa	109-LGLPWK	
		131-LMVQVKK	
	23–25 kDa	176-KLPNSKQ--	
		215-GDVMDVF--	
		280-PGGFGNQ--	
	15–20 kDa	76-NYPKDNK--	
		215-GDVMDVF--	
FTLD-U	23–25 kDa	219-DVFIPKPF--	Nonaka *et al.*^47^
		247-DLIIKGI--	
FTLD-U	22 kDa	208-REFFSQY--	Igaz *et al.*^44^

**Table 4:** List of modifications in cases 1 and 2.

Residue	Amino acid	Modification	Case1	Case2
70	Asparagine	deamidation	O	O
76	Asparagine	deamidation	O	
79	Lysine	ubiquitination		O
82	Lysine	acetylation		O
85	Methionine	oxidation	O	
162	Methionine	oxidation	O	
179	Asparagine	deamidation	O	
213	Glutamine	deamidation	O	O
218	Methionine	oxidation	O	O
242	Serine	phosphorylation	O	O
259	Asparagine	deamidation	O	O
279	Asparagine	deamidation	O	O
285	Asparagine	deamidation	O	O
286	Glutamine	deamidation	O	O
291	Asparagine	deamidation	O	O
292	Serine	phosphorylation		O
301	Asparagine	deamidation	O	O
302	Asparagine	deamidation	O	O
303	Glutamine	deamidation	O	O
305	Serine	phosphorylation	O	O
306	Asparagine	deamidation	O	O
307	Methionine	oxidation	O	O
311	Methionine	oxidation	O	O
312	Asparagine	deamidation	O	O
317	Serine	phosphorylation	O	O
319	Asparagine	deamidation	O	O
322	Methionine	oxidation	O	O
323	Methionine	oxidation	O	O
327	Glutamine	deamidation	O	O
331	Glutamine	deamidation		O
332	Serine	phosphorylation		O
333	Serine	phosphorylation		O
336	Methionine	oxidation	O	O
337	Methionine	oxidation		O
339	Methionine	oxidation		O
346	Glutamine	deamidation	O	
352	Asparagine	deamidation	O	
353	Asparagine	deamidation	O	
354	Glutamine	deamidation	O	
355	Asparagine	deamidation	O	
356	Glutamine	deamidation	O	O
358	Asparagine	deamidation	O	
359	Methionine	oxidation	O	
366	Glutamine	deamidation	O	
372	Asparagine	deamidation		O
373	Serine	phosphorylation		O
375	Serine	phosphorylation	O	O
378	Asparagine	deamidation	O	
379	Serine	phosphorylation		O
387	Serine	phosphorylation	O	O
389	Serine	phosphorylation	O	O
390	Asparagine	deamidation		O
393	Serine	phosphorylation	O	O
395	Serine	phosphorylation	O	O
398	Asparagine	deamidation	O	O
403	Serine	phosphorylation	O	
404	Serine	phosphorylation	O	O
405	Methionine	oxidation	O	O
407	Serine	phosphorylation		O
409	Serine	phosphorylation		O

## References

[b1] Buratti E. *et al.* TDP-43 Binds Heterogeneous Nuclear Ribonucleoprotein A/B through Its C-terminal Tail: An important region for the inhibition of cystic fibrosis transmembrane conductance regulatior exon 9 splicing. J. Biol. Chem. 280, 37572–37584, 10.1074/jbc.M505557200 (2005). 16157593

[b2] Baralle M., Buratti E. & Baralle F. E. The role of TDP-43 in the pathogenesis of ALS and FTLD. Biochem Soc Trans 41, 1536–1540, 10.1042/bst20130186 (2013). 24256250

[b3] Ayala Y. M., Misteli T. & Baralle F. E. TDP-43 regulates retinoblastoma protein phosphorylation through the repression of cyclin-dependent kinase 6 expression. Proc Natl Acad Sci USA 105, 3785–3789, 10.1073/pnas.0800546105 (2008). 18305152 PMC2268791

[b4] Ayala Y. M., Pagani F. & Baralle F. E. TDP43 depletion rescues aberrant CFTR exon 9 skipping. FEBS Lett 580, 1339–1344, 10.1016/j.febslet.2006.01.052 (2006). 16458894

[b5] Corrado L. *et al.* High frequency of *TARDBP* gene mutations in Italian patients with amyotrophic lateral sclerosis. Human Mutation 30, 688–694 (2009). 19224587 10.1002/humu.20950

[b6] Buratti E. & Baralle F. E. Multiple roles of TDP-43 in gene expression, splicing regulation, and human disease. Front Biosci 13, 867–878 (2008). 17981595 10.2741/2727

[b7] Buratti E. *et al.* Nuclear factor TDP-43 and SR proteins promote *in vitro* and *in vivo* CFTR exon 9 skipping. EMBO J 20, 1774–1784, 10.1093/emboj/20.7.1774 (2001). 11285240 PMC145463

[b8] Bose J. K., Wang I. F., Hung L., Tarn W.-Y. & Shen C. K. J. TDP-43 overexpression enhances exon-7 inclusion during SMN Pre-mRNA splicing. J. Biol. Chem. 283, 28852–28859, 10.1074/jbc.M805376200 (2008). 18703504 PMC2661999

[b9] Ou S. H., Wu F., Harrich D., Garcia-Martinez L. F. & Gaynor R. B. Cloning and characterization of a novel cellular protein, TDP-43, that binds to human immunodeficiency virus type 1 TAR DNA sequence motifs. J. Virol. 69, 3584–3596 (1995). 7745706 10.1128/jvi.69.6.3584-3596.1995 PMC189073

[b10] Wang H.-Y., Wang I. F., Bose J. & Shen C. K. J. Structural diversity and functional implications of the eukaryotic TDP gene family. Genomics 83, 130–139 (2004). 14667816 10.1016/s0888-7543(03)00214-3

[b11] Wang I. F., Reddy N. M. & Shen C. K. Higher order arrangement of the eukaryotic nuclear bodies. Proc Natl Acad Sci USA 99, 13583–13588, 10.1073/pnas.212483099 (2002). 12361981 PMC129717

[b12] Sephton C. F. *et al.* TDP-43 Is a Developmentally Regulated Protein Essential for Early Embryonic Development. Journal of Biological Chemistry 285, 6826–6834, 10.1074/jbc.M109.061846 (2010). 20040602 PMC2825476

[b13] Wu L.-S. *et al.* TDP-43, a neuro-pathosignature factor, is essential for early mouse embryogenesis. genesis 48, 56–62, 10.1002/dvg.20584 (2010). 20014337

[b14] Neumann M. *et al.* Ubiquitinated TDP-43 in Frontotemporal Lobar Degeneration and Amyotrophic Lateral Sclerosis. Science 314, 130–133, 10.1126/science.1134108 (2006). 17023659

[b15] Arai T. *et al.* TDP-43 is a component of ubiquitin-positive tau-negative inclusions in frontotemporal lobar degeneration and amyotrophic lateral sclerosis. Biochemical and Biophysical Research Communications 351, 602–611 (2006). 17084815 10.1016/j.bbrc.2006.10.093

[b16] Yokota O. *et al.* Phosphorylated TDP-43 pathology and hippocampal sclerosis in progressive supranuclear palsy. Acta Neuropathol 120, 55–66, 10.1007/s00401-010-0702-1 (2010). 20512649 PMC2901929

[b17] Arai T. *et al.* Phosphorylated TDP-43 in Alzheimer’s disease and dementia with Lewy bodies. Acta Neuropathol 117, 125–136, 10.1007/s00401-008-0480-1 (2009). 19139911

[b18] Hasegawa M. *et al.* TDP-43 is deposited in the Guam parkinsonism-dementia complex brains. Brain 130, 1386–1394, 10.1093/brain/awm065 (2007). 17439983

[b19] Tan C. F. *et al.* Selective occurrence of TDP-43-immunoreactive inclusions in the lower motor neurons in Machado-Joseph disease. Acta Neuropathol 118, 553–560, 10.1007/s00401-009-0552-x (2009). 19526244

[b20] Toyoshima Y. *et al.* Spinocerebellar ataxia type 2 (SCA2) is associated with TDP-43 pathology. Acta Neuropathol 122, 375–378, 10.1007/s00401-011-0862-7 (2011). 21830155

[b21] Kabashi E. *et al.* TARDBP mutations in individuals with sporadic and familial amyotrophic lateral sclerosis. Nat Genet 40, 572–574 (2008). 18372902 10.1038/ng.132

[b22] Sreedharan J. *et al.* TDP-43 Mutations in Familial and Sporadic Amyotrophic Lateral Sclerosis. Science 319, 1668–1672, 10.1126/science.1154584 (2008). 18309045 PMC7116650

[b23] Mackenzie I. R., Rademakers R. & Neumann M. TDP-43 and FUS in amyotrophic lateral sclerosis and frontotemporal dementia. Lancet Neurol 9, 995–1007, 10.1016/S1474-4422(10)70195-2 (2010). 20864052

[b24] Tamaoka A. *et al.* TDP-43 M337V mutation in familial amyotrophic lateral sclerosis in Japan. Intern Med 49, 331–334 (2010). 20154440 10.2169/internalmedicine.49.2915

[b25] Pesiridis G. S., Lee V. M. & Trojanowski J. Q. Mutations in TDP-43 link glycine-rich domain functions to amyotrophic lateral sclerosis. Hum Mol Genet 18, R156–162, 10.1093/hmg/ddp303 (2009). 19808791 PMC2758707

[b26] Tsuji H. *et al.* Molecular analysis and biochemical classification of TDP-43 proteinopathy. Brain 135, 3380–3391, 10.1093/brain/aws230 (2012). 23035040

[b27] Hasegawa M. *et al.* Phosphorylated TDP-43 in frontotemporal lobar degeneration and amyotrophic lateral sclerosis. Annals of Neurology 64, 60–70 (2008). 18546284 10.1002/ana.21425 PMC2674108

[b28] Neumann M., Kwong L. K., Sampathu D. M., Trojanowski J. Q. & Lee V. M. TDP-43 proteinopathy in frontotemporal lobar degeneration and amyotrophic lateral sclerosis: protein misfolding diseases without amyloidosis. Arch Neurol 64, 1388–1394, 10.1001/archneur.64.10.1388 (2007). 17923623

[b29] Neumann M. *et al.* TDP-43 in the ubiquitin pathology of frontotemporal dementia with VCP gene mutations. J Neuropathol Exp Neurol 66, 152–157, 10.1097/nen.0b013e31803020b9 (2007). 17279000

[b30] Zhang Y. J. *et al.* Aberrant cleavage of TDP-43 enhances aggregation and cellular toxicity. Proc Natl Acad Sci USA 106, 7607–7612, 10.1073/pnas.0900688106 (2009). 19383787 PMC2671323

[b31] Hasegawa M. *et al.* Molecular analysis and biochemical classification of TDP-43 proteinopathy. Dementia and Geriatric Cognitive Disorders 33, 103–104 (2012).

[b32] Inukai Y. *et al.* Abnormal phosphorylation of Ser409/410 of TDP-43 in FTLD-U and ALS. FEBS Letters 582, 2899–2904 (2008). 18656473 10.1016/j.febslet.2008.07.027

[b33] Nonaka T. *et al.* Prion-like Properties of Pathological TDP-43 Aggregates from Diseased Brains. Cell Reports 4, 124–134, 10.1016/j.celrep.2013.06.007 (2013). 23831027

[b34] Li Q., Yokoshi M., Okada H. & Kawahara Y. The cleavage pattern of TDP-43 determines its rate of clearance and cytotoxicity. Nat Commun 6, 10.1038/ncomms7183 (2015). 25630387

[b35] Wang I. F. *et al.* The self-interaction of native TDP-43 C terminus inhibits its degradation and contributes to early proteinopathies. Nat Commun 3, 766, 10.1038/ncomms1766 (2012). 22473010

[b36] Yamashita T. *et al.* A role for calpain-dependent cleavage of TDP-43 in amyotrophic lateral sclerosis pathology. Nat Commun 3, 1307, 10.1038/ncomms2303 (2012). 23250437 10.1038/ncomms2303

[b37] Aggad D., Vérièpe J., Tauffenberger A. & Parker J. A. TDP-43 toxicity proceeds via calcium dysregulation and necrosis in aging Caenorhabditis elegans motor neurons. J Neurosci 34, 12093–12103, 10.1523/JNEUROSCI.2495-13.2014 (2014). 25186754 PMC4262699

[b38] Kametani F. *et al.* Identification of casein kinase-1 phosphorylation sites on TDP-43. Biochemical and Biophysical Research Communications 382, 405–409, 10.1016/j.bbrc.2009.03.038 (2009). 19285963

[b39] Cohen T. J. *et al.* An acetylation switch controls TDP-43 function and aggregation propensity. Nat Commun 6, 10.1038/ncomms6845 (2015). PMC4407365 25556531

[b40] Suzuki H., Lee K. & Matsuoka M. TDP-43-induced death is associated with altered regulation of BIM and Bcl-xL and attenuated by caspase-mediated TDP-43 cleavage. J Biol Chem 286, 13171–13183, 10.1074/jbc.M110.197483 (2011). 21339291 PMC3075664

[b41] Chew J. *et al.* C9ORF72 repeat expansions in mice cause TDP-43 pathology, neuronal loss, and behavioral deficits. Science 348, 1151–1154, 10.1126/science.aaa9344 (2015). 25977373 PMC4692360

[b42] Fang Y. S. *et al.* Full-length TDP-43 forms toxic amyloid oligomers that are present in frontotemporal lobar dementia-TDP patients. Nat Commun 5, 4824, 10.1038/ncomms5824 (2014). 25215604

[b43] Janssens J. *et al.* Overexpression of ALS-associated p.M337V human TDP-43 in mice worsens disease features compared to wild-type human TDP-43 mice. Mol Neurobiol 48, 22–35, 10.1007/s12035-013-8427-5 (2013). 23475610 PMC3718993

[b44] Igaz L. M. *et al.* Dysregulation of the ALS-associated gene TDP-43 leads to neuronal death and degeneration in mice. J Clin Invest 121, 726–738, 10.1172/JCI44867 (2011). 21206091 PMC3026736

[b45] D’Alton S. *et al.* Divergent Phenotypes in Mutant TDP-43 Transgenic Mice Highlight Potential Confounds in TDP-43 Transgenic Modeling. PLoS ONE 9, e86513, 10.1371/journal.pone.0086513 (2014). 24466128 PMC3899264

[b46] Igaz L. M. *et al.* Expression Of TDP-43 C-terminal fragments *in vitro* recapitulates pathological features of TDP-43 proteinopathies. J. Biol. Chem. 284, 8516-8524, M809462200, 10.1074/jbc.M809462200 (2009). PMC2659210 19164285

[b47] Nonaka T., Kametani F., Arai T., Akiyama H. & Hasegawa M. Truncation and pathogenic mutations facilitate the formation of intracellular aggregates of TDP-43. Hum. Mol. Genet. 18, 3353–3364, 10.1093/hmg/ddp275 (2009). 19515851

[b48] Igaz L. M. *et al.* Enrichment of C-Terminal Fragments in TAR DNA-Binding Protein-43 Cytoplasmic Inclusions in Brain but not in Spinal Cord of Frontotemporal Lobar Degeneration and Amyotrophic Lateral Sclerosis. Am J Pathol 173, 182–194, 10.2353/ajpath.2008.080003 (2008). 18535185 PMC2438296

[b49] Lee E. B., Lee V. M. Y. & Trojanowski J. Q. Gains or losses: molecular mechanisms of TDP43-mediated neurodegeneration. Nat Rev Neurosci 13, 38–50 (2012). 22127299 10.1038/nrn3121 PMC3285250

[b50] Guo W. *et al.* An ALS-associated mutation affecting TDP-43 enhances protein aggregation, fibril formation and neurotoxicity. Nat Struct Mol Biol 18, 822–830, 10.1038/nsmb.2053 (2011). 21666678 PMC3357956

[b51] Chen A. K. *et al.* Induction of amyloid fibrils by the C-terminal fragments of TDP-43 in amyotrophic lateral sclerosis. J Am Chem Soc 132, 1186–1187, 10.1021/ja9066207 (2010). 20055380

[b52] Wang Y.-T. *et al.* The Truncated C-terminal RNA Recognition Motif of TDP-43 Protein Plays a Key Role in Forming Proteinaceous Aggregates. Journal of Biological Chemistry 288, 9049–9057, 10.1074/jbc.M112.438564 (2013). 23372158 PMC3610977

