# Supplementary material for: Mass spectrometric analysis of accumulated TDP-43 in amyotrophic lateral sclerosis brains
Source: Sci Rep. 2016 Mar 16;6:23281. doi: 10.1038/srep23281 (PMC4793195; doi:10.1038/srep23281)
Supplement: Supplementary Information [file srep23281-s1.pdf]

Mass spectrometric analysis of accumulated TDP-43 in amyotrophic lateral sclerosis brains

Fuyuki Kametani[1*], Tomokazu Obi[2], Takeo Shishido[2], Hiroyasu Akatsu[3], Shigeo Murayama[4], Yuko Saito[5], Mari Yoshida[6], Masato Hasegawa[1*]

1) Department of Dementia and Higher Brain Function, Tokyo Metropolitan Institute of Medical Science, Setagaya-ku, Tokyo 156-8506, Japan
2) National Epilepsy Center, Shizuoka Institute of Epilepsy and Neurological Disorders, Urushiyama 886, Aoi-ku, Shizuoka 420-8688, Japan
3) Choju Medical Institute, Fukushimura Hospital, Noyorimachiazayamanaka, Toyohashi 441-8124, Japan
4) Department of Neuropathology (Brain Bank for Aging Research), Tokyo Metropolitan Geriatric Hospital & Institute of Gerontology, Itabashi-ku, Tokyo 173-0015, Japan
5) Department of Laboratory Medicine, National Center Hospital, NCNP, 4-1-1 Ogawahigashi, Kodaira, Tokyo 187-8502, Japan
6) Department of Neuropathology, Institute for Medical Science of Aging, Aichi Medical University, Nagakute, Aichi 480-1195, Japan

Keywords

TDP-43, inclusion body, phosphorylation, fragmentation, frontotemporal lobar degeneration with ubiquitin-positive inclusions, amyotrophic lateral sclerosis, proteomics, mass spectrometry.

***Corresponding author**
Fuyuki Kametani
Department of Dementia and Higher Brain Function Research
Tokyo Metropolitan Institute of Medical Science, Tokyo 156-8506, Japan
TEL: +81-3-6834-2349, E-mail: kametani-fy@igakuken.or.jp
Masato Hasegawa
Department of Dementia and Higher Brain Function Research
Tokyo Metropolitan Institute of Medical Science, Tokyo 156-8506, Japan
TEL: +81-3-6834-2349, E-mail: hasegawa-ms@igakuken.or.jp

Figure legends

Supplemental Figure S1

Identification of cleavage site peptides by LC-MS/MS analysis. Product ion spectrum of a mass signal of tryptic or chymotrypic peptides detected in gel bands from case 1 (A-E) and from case 2 (G-L), showing the b/a and y ion series. These peptides were listed in Table1 and 2. Cleavage sites were indicated by arrows in Figure 3.

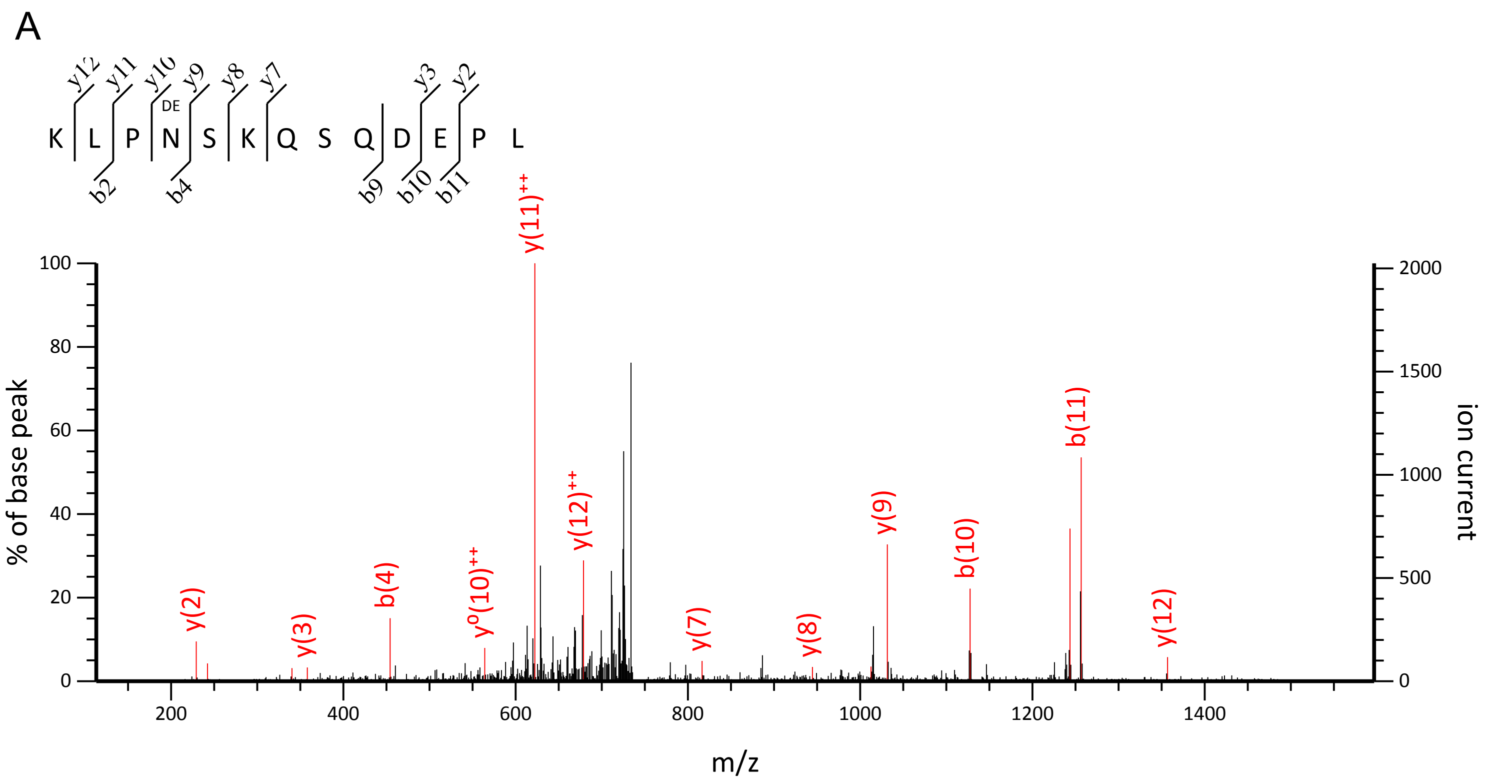
A
K L P N S K Q S Q D E P L
DE
y12 y11 y10 y9 y8 y7 y3 y2
b2 b4 b9 b10 b11
y(2)
y(3)
b(4)
y°(10)++
y(11)++
y(12)++
y(7)
y(8)
y(9)
b(10)
b(11)
y(12)
% of base peak
ion current
m/z

Figure S1

B

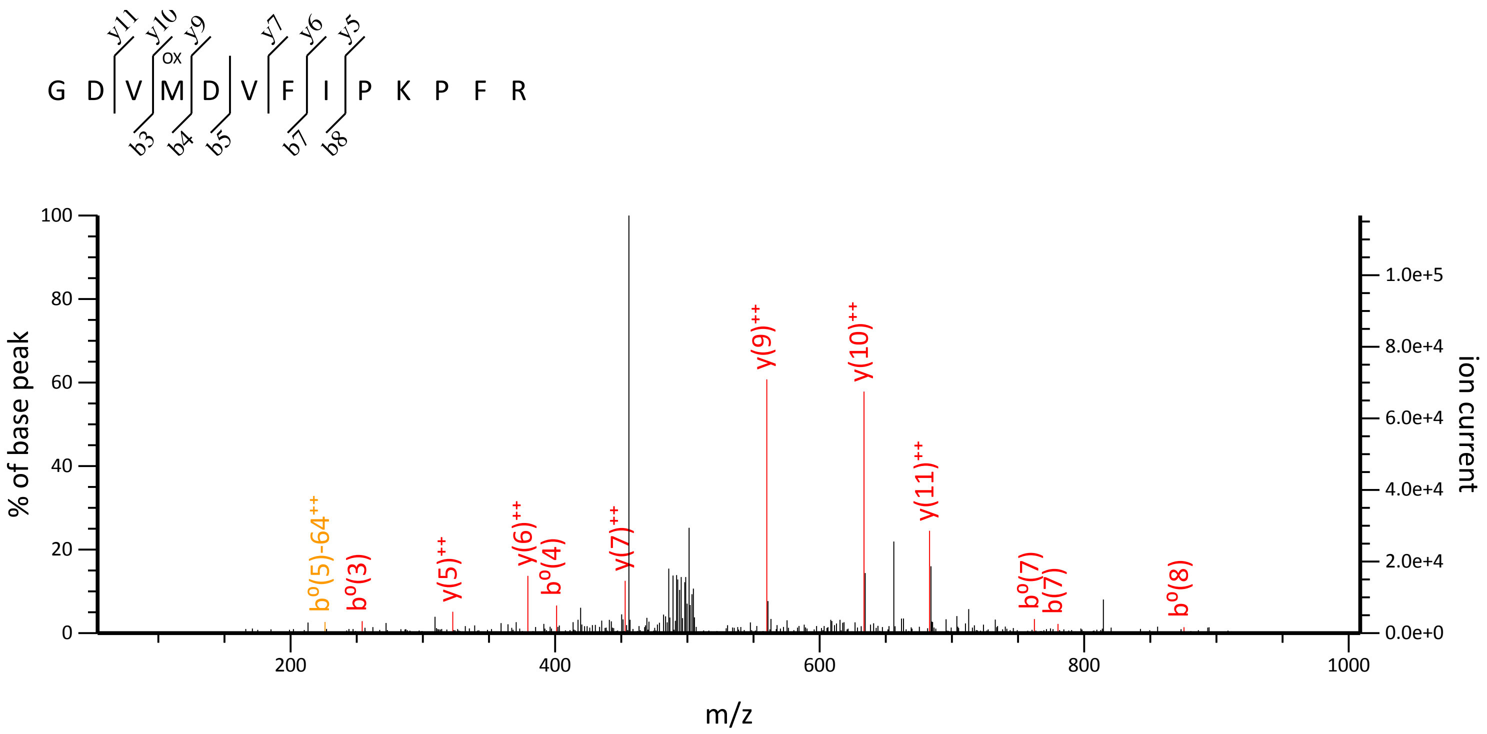

y11 y10 y9 y7 y6 y5
ox
G D V M D V F I P K P F R
b3 b4 b5 b7 b8
100
80
60
40
20
0
% of base peak
200
400
600
800
1000
m/z
1.0e+5
8.0e+4
6.0e+4
4.0e+4
2.0e+4
0.0e+0
ion current
b°(5)-64++
b°(3)
y(5)++
y(6)++
b°(4)
y(7)++
y(9)++
y(10)++
y(11)++
b°(7)
b(7)
b°(8)

Figure S1

C

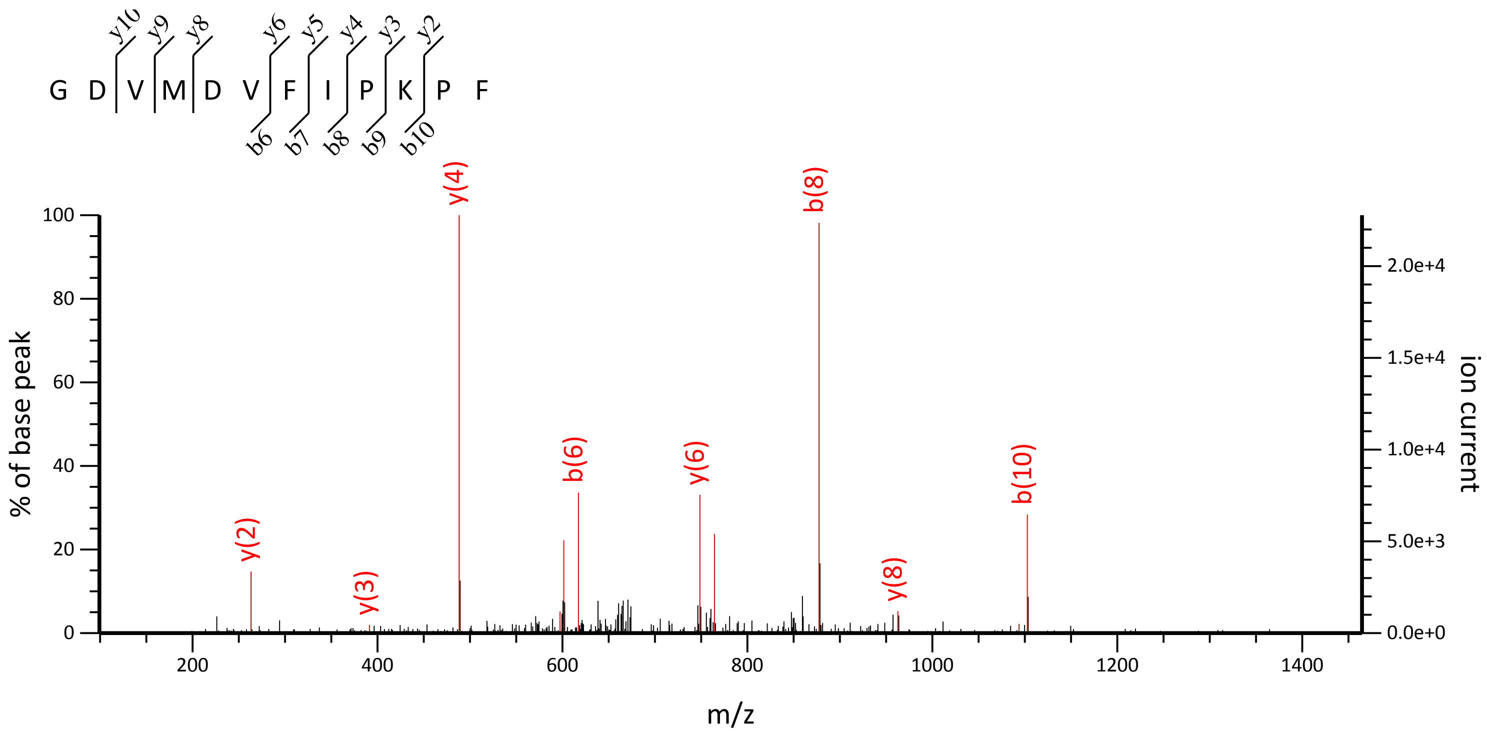

G D V M D V F I P K P F
y10 y9 y8 y6 y5 y4 y3 y2
b6 b7 b8 b9 b10
y(4)
b(8)
b(6)
y(6)
b(10)
y(2)
y(3)
y(8)
% of base peak
ion current
m/z

Figure S1

D

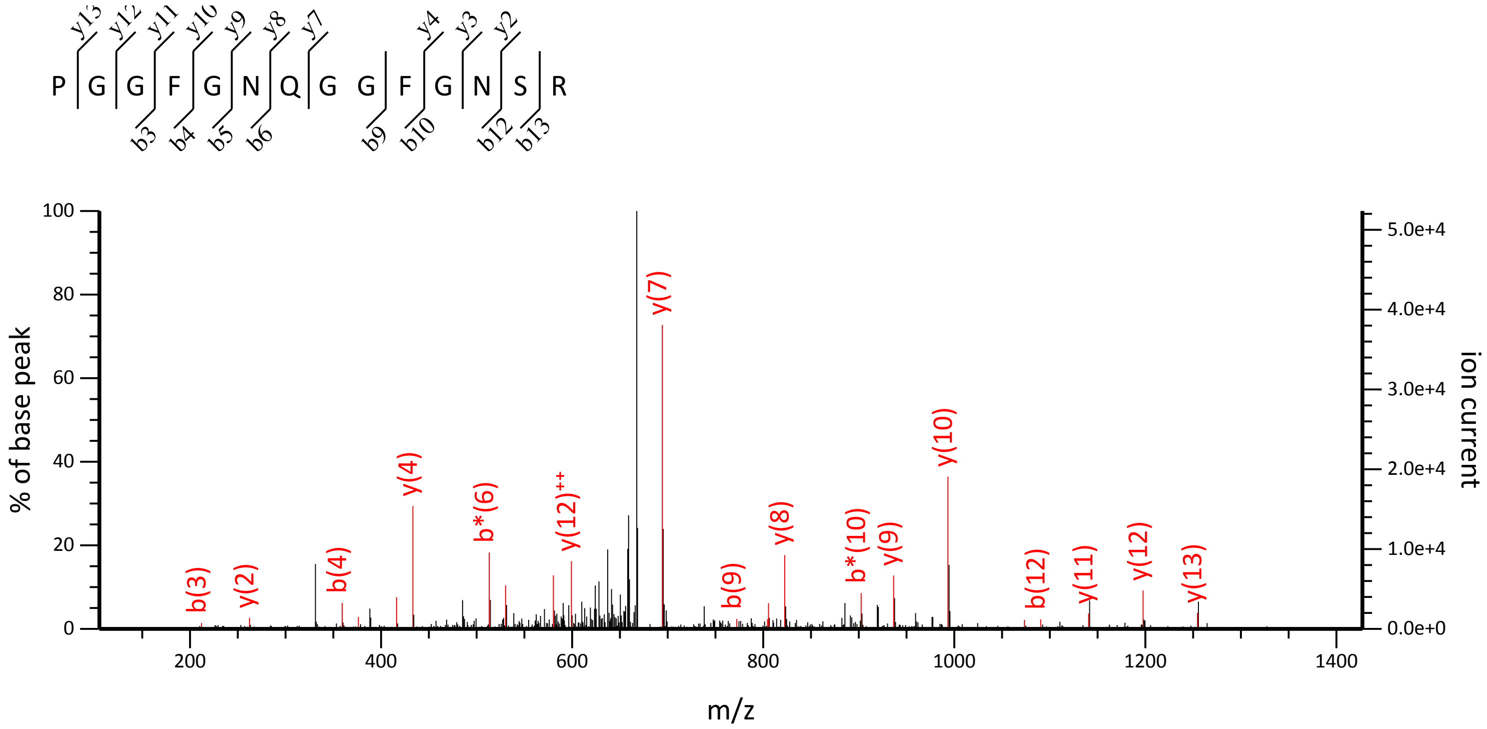
P G G F G N Q G G F G N S R
y13 y12 y11 y10 y9 y8 y7 y4 y3 y2
b3 b4 b5 b6 b9 b10 b12 b13
% of base peak
ion current
m/z
b(3)
y(2)
b(4)
y(4)
b*(6)
y(12)++
y(7)
b(9)
y(8)
b*(10)
y(9)
y(10)
b(12)
y(11)
y(12)
y(13)

Figure S1

E

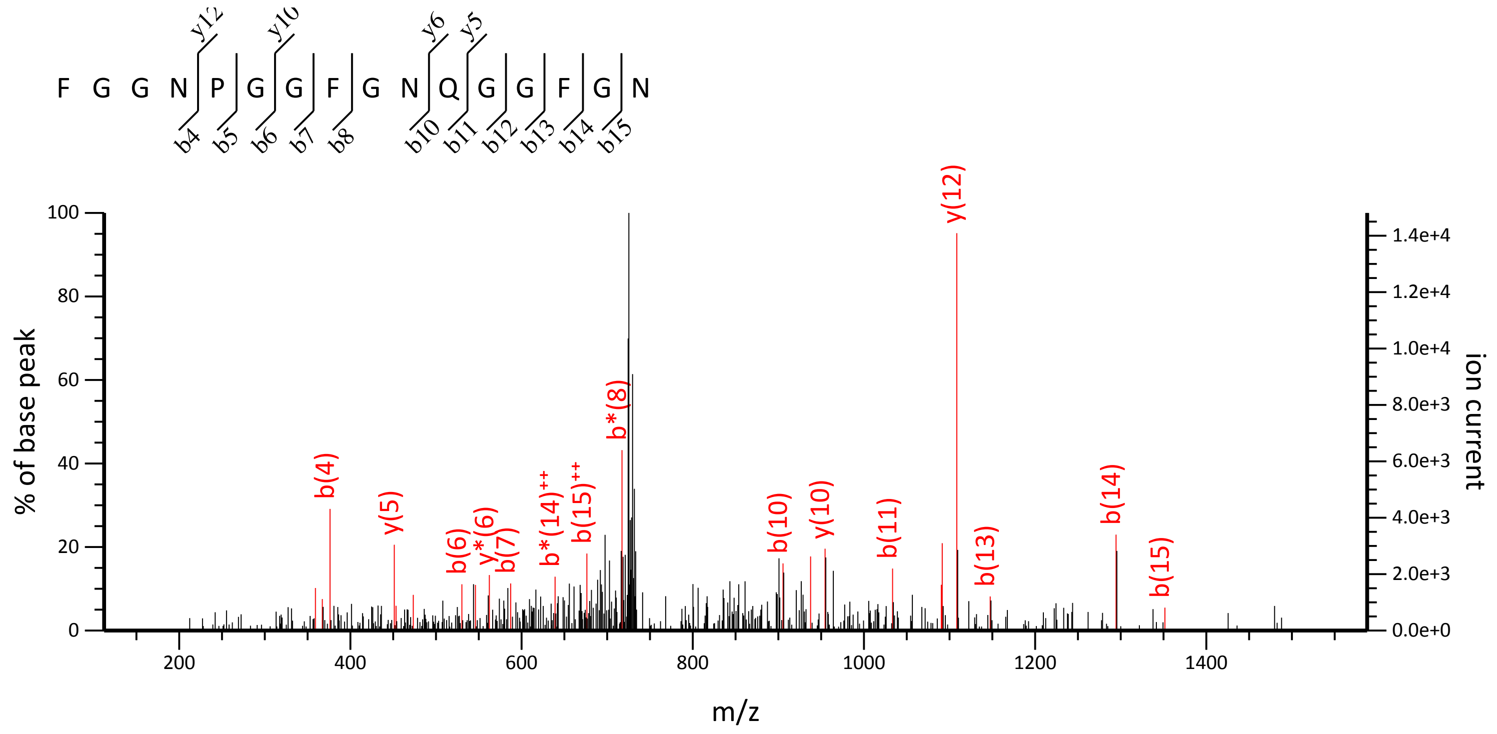

F G G N P G G F G N Q G G F G N
y12
y10
y6
y5
b4
b5
b6
b7
b8
b10
b11
b12
b13
b14
b15
% of base peak
ion current
m/z
b(4)
y(5)
b(6)
y*(6)
b(7)
b*(14)++
b(15)++
b*(8)
b(10)
y(10)
b(11)
y(12)
b(13)
b(14)
b(15)

Figure S1

F

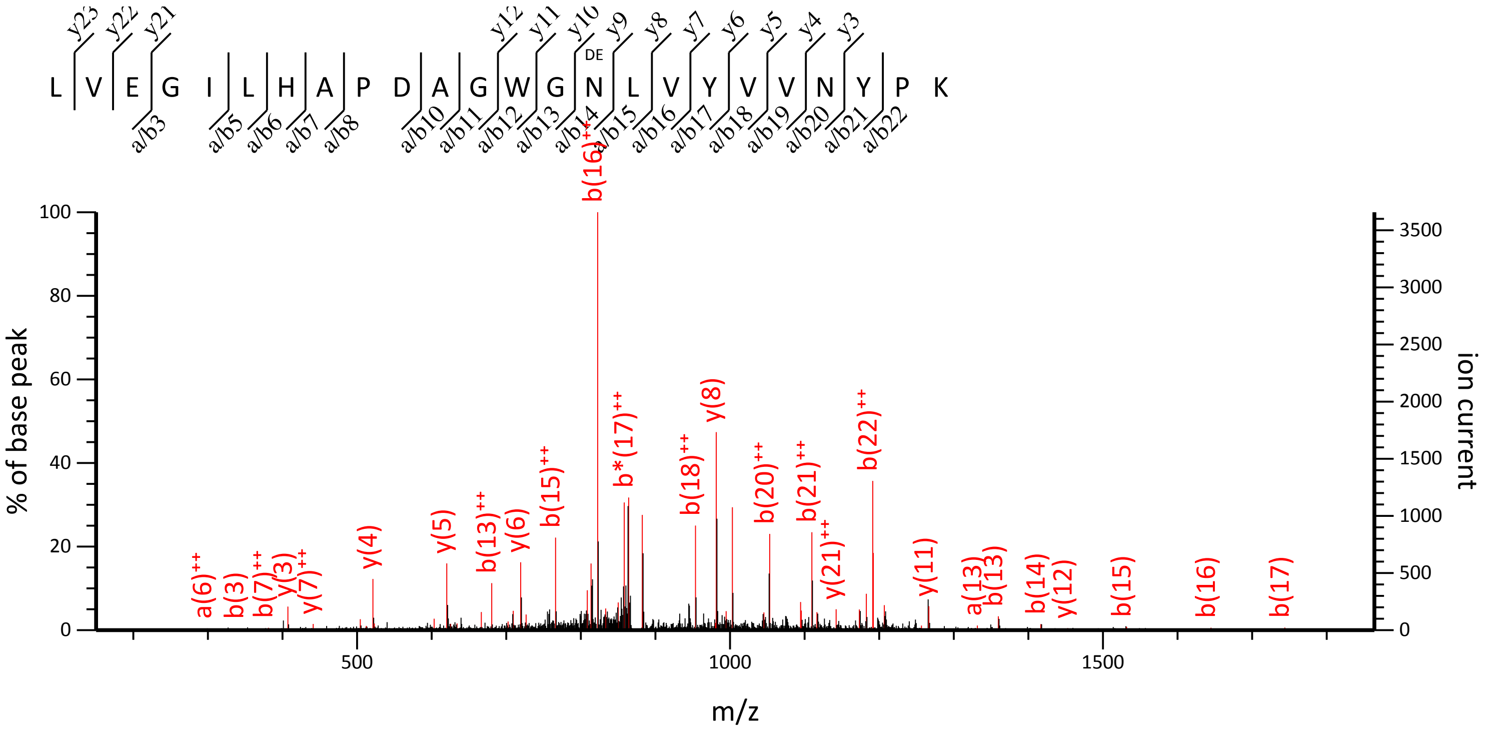

L V E G I L H A P D A G W G N L V Y V V N Y P K
DE
% of base peak
ion current
m/z
b(16)++
b*(17)++
y(8)
b(22)++
b(18)++
b(20)++
b(21)++
b(15)++
y(21)++
y(11)
y(6)
b(13)++
y(5)
y(4)
a(6)++
b(3)
b(7)++
y(3)
y(7)++
a(13)
b(13)
b(14)
y(12)
b(15)
b(16)
b(17)

Figure S1

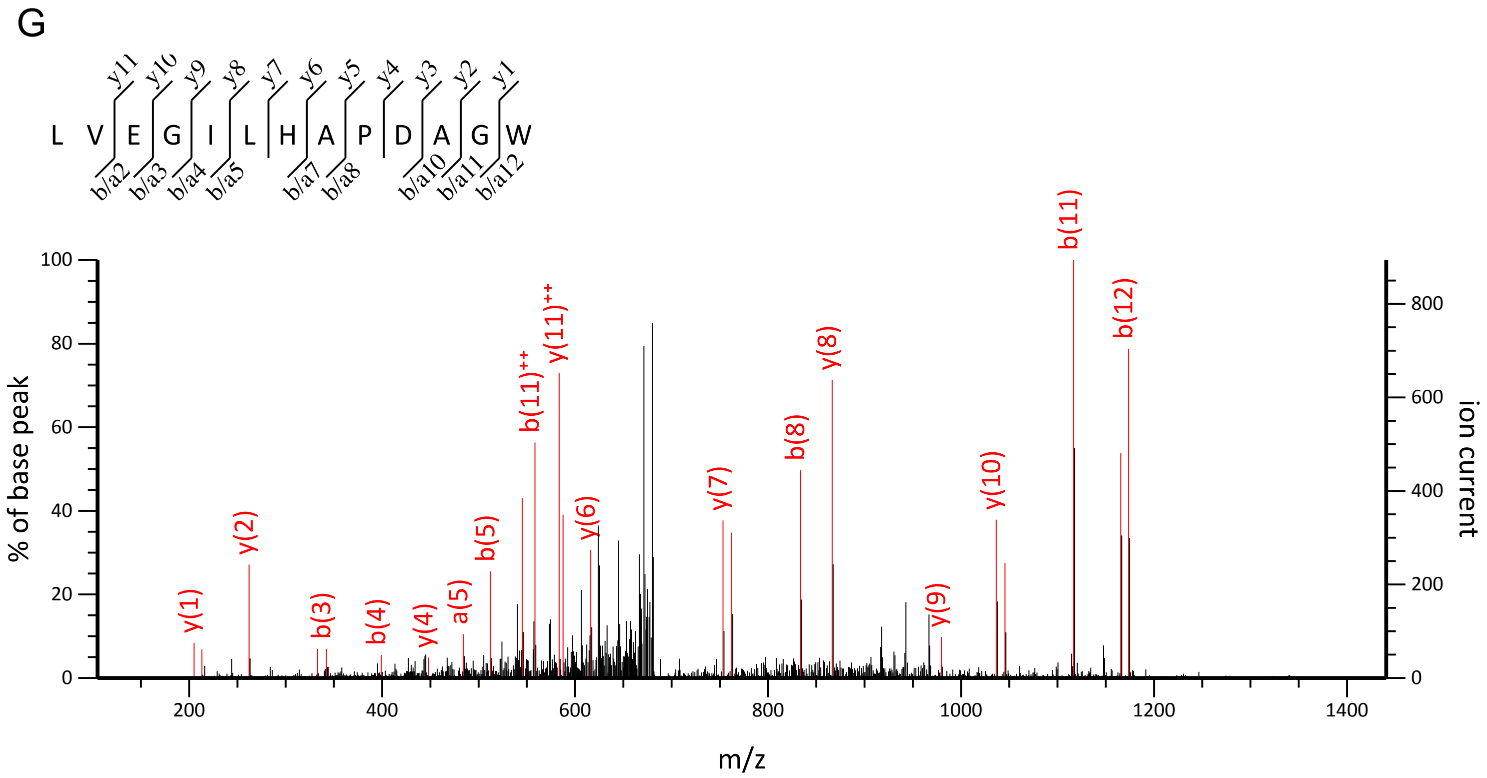
G
L V E G I L H A P D A G W
y11 y10 y9 y8 y7 y6 y5 y4 y3 y2 y1
b/a2 b/a3 b/a4 b/a5 b/a7 b/a8 b/a10 b/a11 b/a12
y(1) y(2) b(3) b(4) y(4) a(5) b(5) b(11)++ y(11)++ y(6) y(7) b(8) y(8) y(9) y(10) b(11) b(12)
% of base peak
ion current
m/z

Figure S1

H

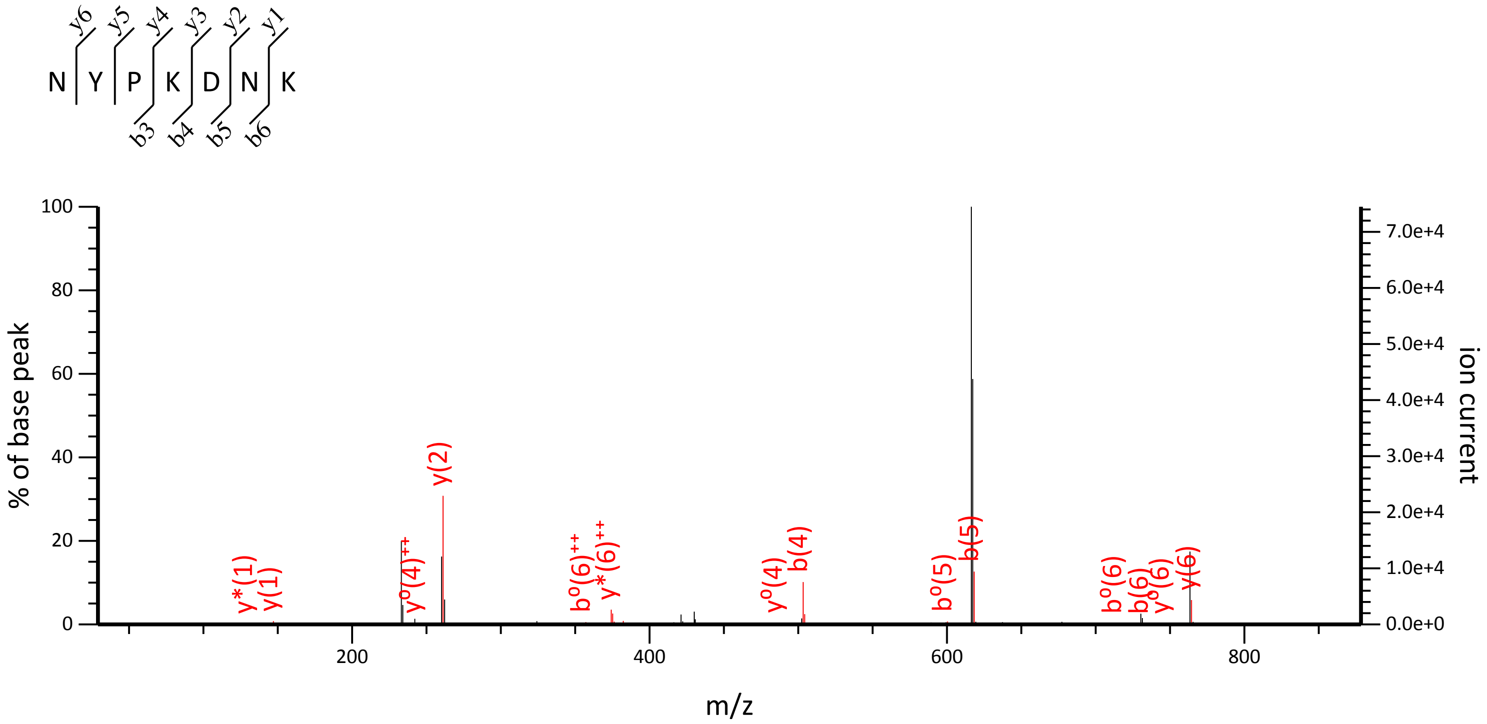

N Y P K D N K
y6 y5 y4 y3 y2 y1
b3 b4 b5 b6
% of base peak
ion current
m/z
y*(1)
y(1)
y(2)
b(4)
b(5)
y(6)

Figure S1

I

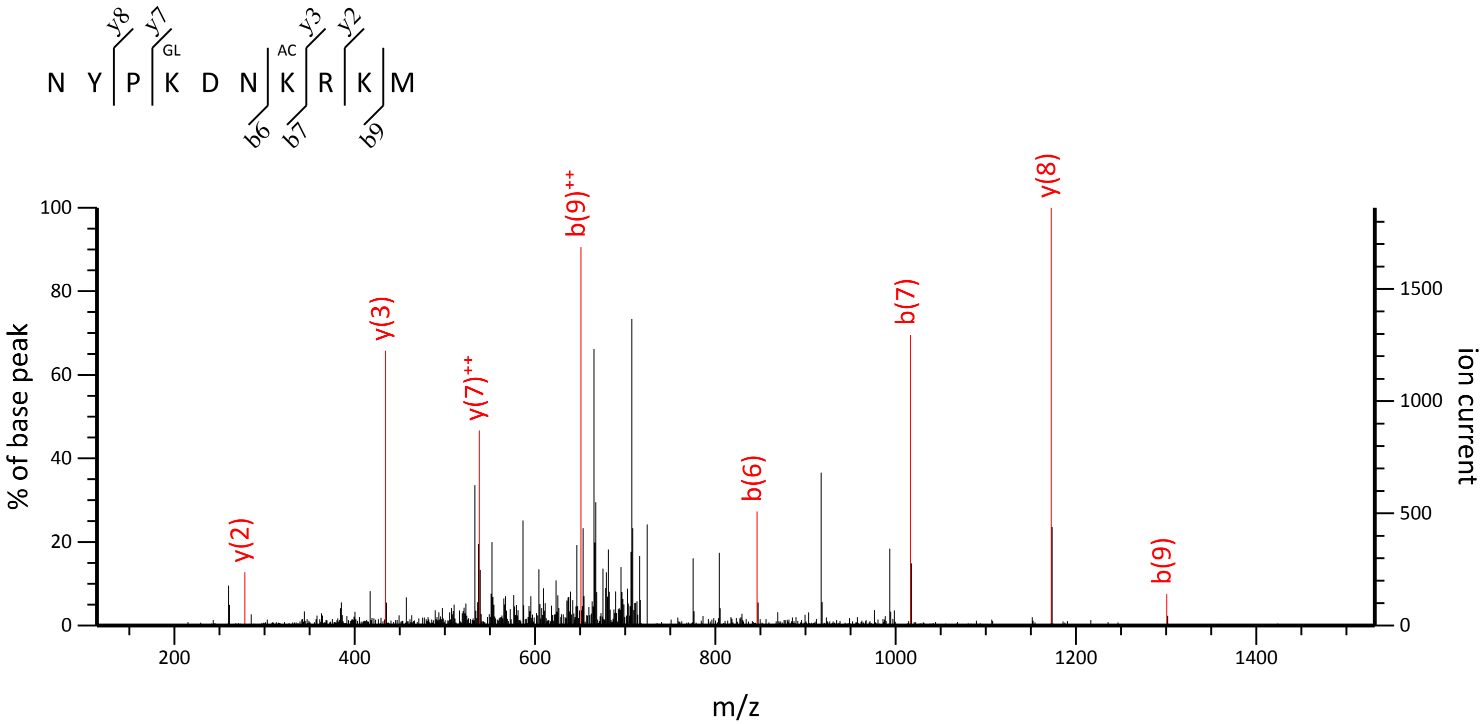

N Y P K D N K R K M
y8
y7
y3
y2
GL
AC
b6
b7
b9
% of base peak
ion current
m/z
y(2)
y(3)
y(7)++
b(9)++
b(6)
b(7)
y(8)
b(9)
0
20
40
60
80
100
0
500
1000
1500
200
400
600
800
1000
1200
1400

Figure S1

J

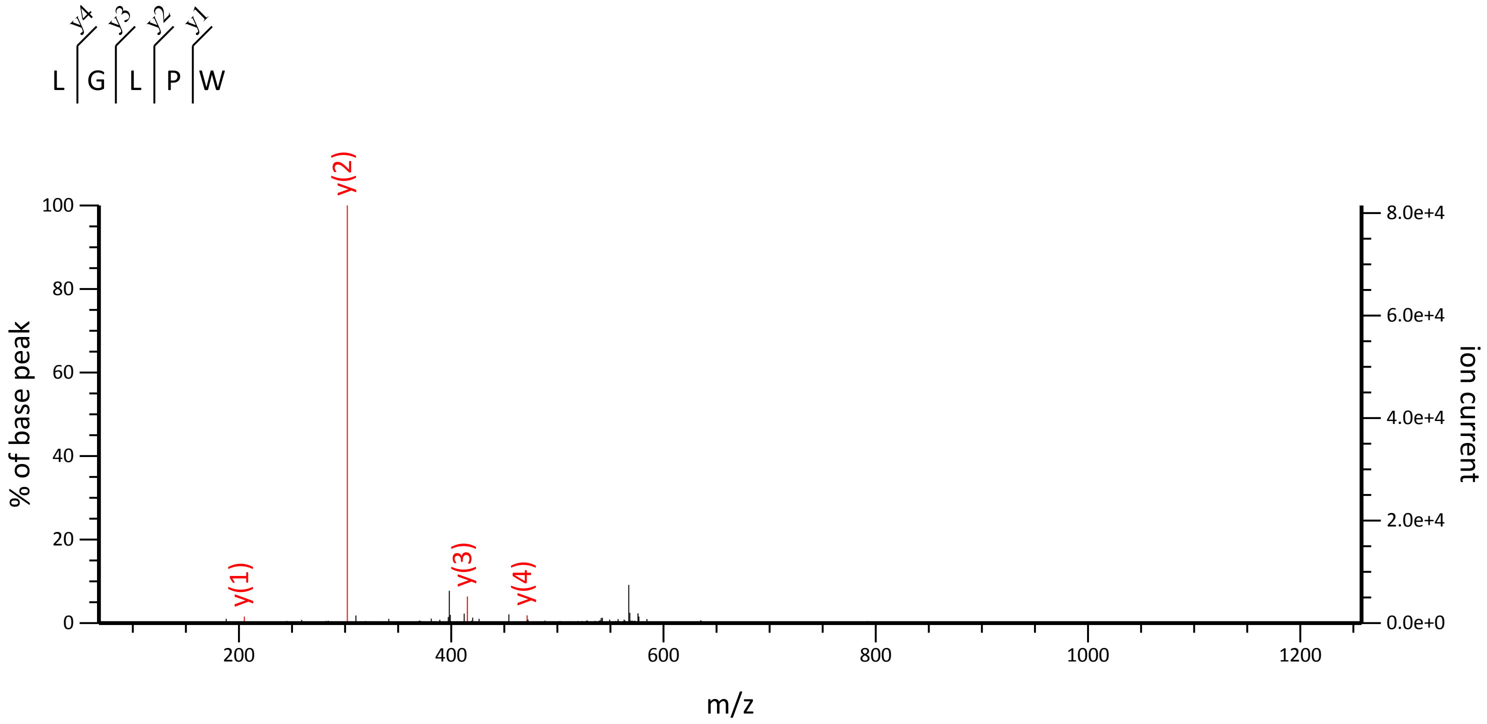

y4
y3
y2
y1
L G L P W
y(2)
y(1)
y(3)
y(4)
100
80
60
40
20
0
% of base peak
8.0e+4
6.0e+4
4.0e+4
2.0e+4
0.0e+0
ion current
200
400
600
800
1000
1200
m/z

Figure S1

K

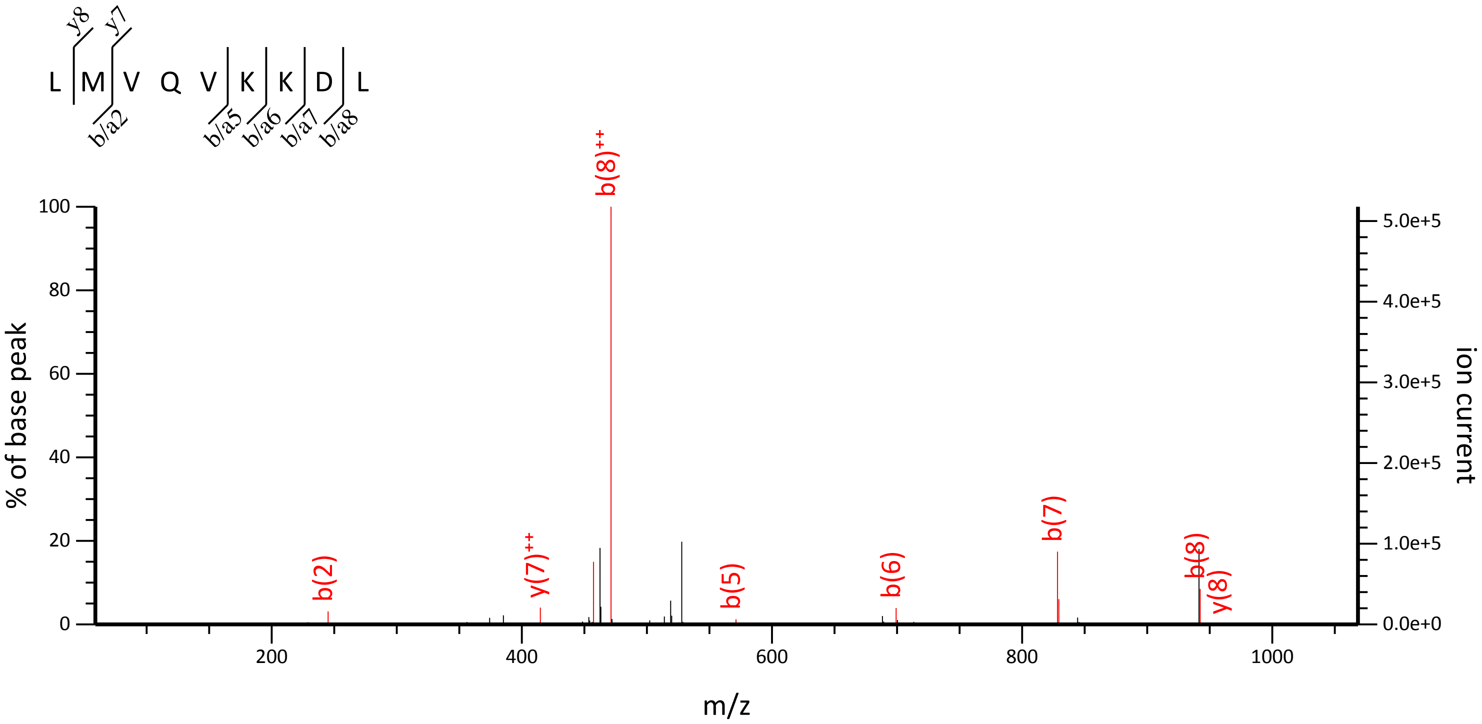

y8
y7
L M V Q V K K D L
b/a2
b/a5
b/a6
b/a7
b/a8
b(8)++
b(2)
y(7)++
b(5)
b(6)
b(7)
b(8)
y(8)
100
80
60
40
20
0
% of base peak
5.0e+5
4.0e+5
3.0e+5
2.0e+5
1.0e+5
0.0e+0
ion current
200
400
600
800
1000
m/z

Figure S1

L

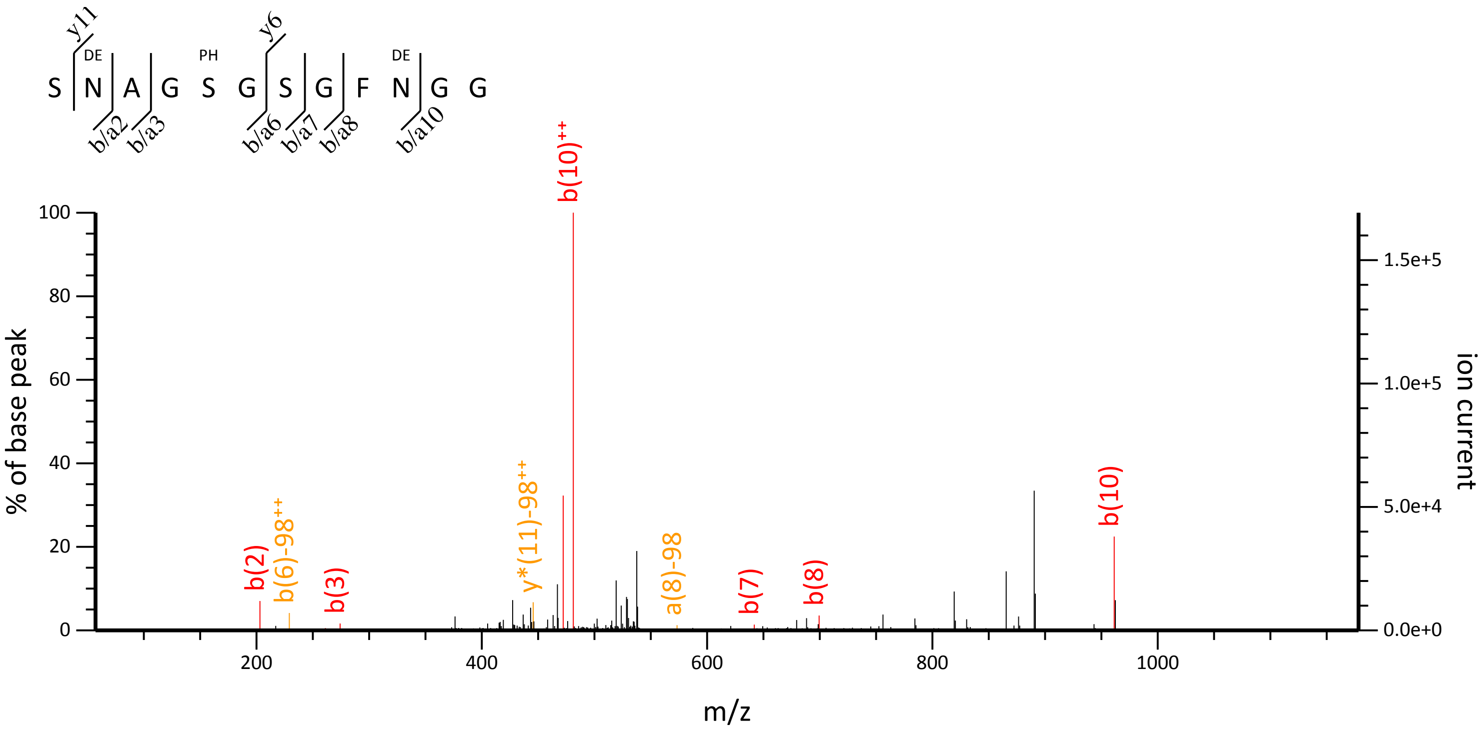

y11
y6
S N A G S G S G F N G G
DE
PH
DE
b/a2
b/a3
b/a6
b/a7
b/a8
b/a10
b(10)++
b(2)
b(6)-98++
b(3)
y*(11)-98++
a(8)-98
b(7)
b(8)
b(10)
% of base peak
ion current
m/z

Figure S1

Supplemental Table S3. List of identified proteins in Sarkosyl-insoluble fraction.

Biological sample category	Biological sample name	MS/MS sample name	Protein name	Protein accession numbers	Database sources	Protein molecular weight (Da)	Protein identification probability	Number of unique peptides	Number of unique spectra	Number of total spectra	Percentage of total spectra	Percentage sequence coverage
Uncategorized Sample	BioSample 1	(F001785)	Myelin proteolipid protein OS=Homo sapiens GN=PLP1 PE=1 SV=2	MYPR_HUMAN	SwissProt_2014_08.fasta	30,077.70	100.00%	3	3	15	0.09%	11.60%
Uncategorized Sample	BioSample 1	(F001785)	Glial fibrillary acidic protein OS=Homo sapiens GN=GFAP PE=1 SV=1	GFAP_HUMAN	SwissProt_2014_08.fasta	49,881.40	100.00%	4	4	7	0.04%	11.60%
Uncategorized Sample	BioSample 1	(F001785)	Proteasome subunit beta type-3 OS=Homo sapiens GN=PSMB3 PE=1 SV=2	PSB3_HUMAN	SwissProt_2014_08.fasta	22,949.60	99.80%	2	2	2	0.01%	16.60%
Uncategorized Sample	BioSample 1	(F001785)	Ferritin light chain OS=Homo sapiens GN=FTL PE=1 SV=2	FRIL_HUMAN	SwissProt_2014_08.fasta	20,020.60	100.00%	12	19	324	1.96%	48.00%
Uncategorized Sample	BioSample 1	(F001785)	Prostaglandin-H2 D-isomerase OS=Homo sapiens GN=PTGDS PE=1 SV=1	PTGDS_HUMAN	SwissProt_2014_08.fasta	21,029.10	100.00%	3	3	4	0.02%	21.60%
Uncategorized Sample	BioSample 1	(F001785)	Tubulin alpha-1A chain OS=Homo sapiens GN=TUBA1A PE=1 SV=1	TBA1A_HUMAN	SwissProt_2014_08.fasta	50,135.70	100.00%	8	8	12	0.07%	27.30%
Uncategorized Sample	BioSample 1	(F001785)	Ras-related protein Rab-18 OS=Homo sapiens GN=RAB18 PE=1 SV=1	RAB18_HUMAN	SwissProt_2014_08.fasta	22,977.50	99.80%	2	2	3	0.02%	11.70%
Uncategorized Sample	BioSample 1	(F001785)	Apolipoprotein E OS=Homo sapiens GN=APOE PE=1 SV=1	APOE_HUMAN	SwissProt_2014_08.fasta	36,153.50	100.00%	3	3	3	0.02%	11.40%
Uncategorized Sample	BioSample 1	(F001785)	Myelin basic protein OS=Homo sapiens GN=MBP PE=1 SV=3	MBP_HUMAN	SwissProt_2014_08.fasta	33,117.70	100.00%	3	3	4	0.02%	11.20%
Uncategorized Sample	BioSample 1	(F001785)	Ferritin heavy chain OS=Homo sapiens GN=FTH1 PE=1 SV=2	FRIH_HUMAN	SwissProt_2014_08.fasta	21,226.20	100.00%	10	11	52	0.32%	38.30%
Uncategorized Sample	BioSample 1	(F001785)	Ras-related protein Rab-2A OS=Homo sapiens GN=RAB2A PE=1 SV=1	RAB2A_HUMAN	SwissProt_2014_08.fasta	23,546.20	100.00%	3	3	5	0.03%	20.30%
Uncategorized Sample	BioSample 1	(F001785)	Phosphomevalonate kinase OS=Homo sapiens GN=PMVK PE=1 SV=3	PMVK_HUMAN	SwissProt_2014_08.fasta	21,995.00	99.80%	2	2	2	0.01%	15.60%
Uncategorized Sample	BioSample 1	(F001785)	Ras-related protein Rap-1b OS=Homo sapiens GN=RAP1B PE=1 SV=1	RAP1B_HUMAN	SwissProt_2014_08.fasta	20,824.90	100.00%	4	4	4	0.02%	29.90%
Uncategorized Sample	BioSample 1	(F001785)	Fibrinogen alpha chain OS=Homo sapiens GN=FGA PE=1 SV=2	FIBA_HUMAN	SwissProt_2014_08.fasta	94,973.40	99.80%	2	2	2	0.01%	3.12%
Uncategorized Sample	BioSample 1	(F001785)	Thy-1 membrane glycoprotein OS=Homo sapiens GN=THY1 PE=1 SV=2	THY1_HUMAN	SwissProt_2014_08.fasta	17,935.20	99.80%	2	2	3	0.02%	15.50%
Uncategorized Sample	BioSample 1	(F001785)	Tubulin beta-2A chain OS=Homo sapiens GN=TUBB2A PE=1 SV=1	TBB2A_HUMAN	SwissProt_2014_08.fasta	49,907.10	99.80%	2	2	3	0.02%	16.60%
Uncategorized Sample	BioSample 1	(F001785)	Transforming protein RhoA OS=Homo sapiens GN=RHOA PE=1 SV=1	RHOA_HUMAN,RHOC_HUMAN	SwissProt_2014_08.fasta	21,768.40	99.80%	2	2	2	0.01%	13.00%
Uncategorized Sample	BioSample 1	(F001785)	Alpha-synuclein OS=Homo sapiens GN=SNCA PE=1 SV=1	SYUA_HUMAN	SwissProt_2014_08.fasta	14,459.30	99.80%	2	2	2	0.01%	27.10%
Uncategorized Sample	BioSample 1	(F001785)	Keratin, type II cytoskeletal 1 OS=Homo sapiens GN=KRT1 PE=1 SV=6	K2C1_HUMAN	SwissProt_2014_08.fasta	66,040.30	100.00%	4	4	5	0.03%	7.14%
Uncategorized Sample	BioSample 1	(F001785)	Peroxiredoxin-1 OS=Homo sapiens GN=PRDX1 PE=1 SV=1	PRDX1_HUMAN	SwissProt_2014_08.fasta	22,110.90	99.80%	2	2	2	0.01%	15.60%
Uncategorized Sample	BioSample 1	(F001785)	Alpha-crystallin B chain OS=Homo sapiens GN=CRYAB PE=1 SV=2	CRYAB_HUMAN	SwissProt_2014_08.fasta	20,159.50	100.00%	9	9	43	0.26%	58.90%
Uncategorized Sample	BioSample 1	(F001785)	Plasmolipin OS=Homo sapiens GN=PLLP PE=1 SV=1	PLLP_HUMAN	SwissProt_2014_08.fasta	19,987.20	99.80%	2	2	2	0.01%	21.40%
Uncategorized Sample	BioSample 1	(F001785)	Protein kinase C gamma type OS=Homo sapiens GN=PRKCG PE=1 SV=3	KPCG_HUMAN	SwissProt_2014_08.fasta	78,448.70	99.80%	2	2	2	0.01%	3.30%
Uncategorized Sample	BioSample 1	(F001785)	Tubulin beta-4A chain OS=Homo sapiens GN=TUBB4A PE=1 SV=2	TBB4A_HUMAN	SwissProt_2014_08.fasta	49,585.50	100.00%	6	6	7	0.04%	17.30%
Uncategorized Sample	BioSample 1	(F001785)	Calcium/calmodulin-dependent protein kinase type II subunit alpha OS=Homo sapiens GN=CAMK2A PE=1 SV=2	KCC2A_HUMAN	SwissProt_2014_08.fasta	54,088.70	100.00%	6	7	12	0.07%	15.10%
Uncategorized Sample	BioSample 1	(F001785)	TAR DNA-binding protein 43 OS=Homo sapiens GN=TARDBP PE=1 SV=1	TADBP_HUMAN	SwissProt_2014_08.fasta	44,739.70	100.00%	3	4	18	0.11%	10.10%
Uncategorized Sample	BioSample 1	(F001785)	Microtubule-associated protein tau OS=Homo sapiens GN=MAPT PE=1 SV=5	TAU_HUMAN	SwissProt_2014_08.fasta	78,927.70	100.00%	2	3	15	0.09%	3.69%
Uncategorized Sample	BioSample 1	(F001785)	Ras-related C3 botulinum toxin substrate 1 OS=Homo sapiens GN=RAC1 PE=1 SV=1	RAC1_HUMAN	SwissProt_2014_08.fasta	21,450.60	99.90%	2	2	3	0.02%	7.81%
Uncategorized Sample	BioSample 1	(F001785)	Pleckstrin homology domain-containing family B member 1 OS=Homo sapiens GN=PLEKHB1 PE=1 SV=1	PKHB1_HUMAN	SwissProt_2014_08.fasta	27,185.70	100.00%	4	5	5	0.03%	21.40%
Uncategorized Sample	BioSample 1	(F001785)	Proteasome subunit beta type-1 OS=Homo sapiens GN=PSMB1 PE=1 SV=2	PSB1_HUMAN	SwissProt_2014_08.fasta	26,490.50	99.80%	2	2	2	0.01%	14.10%
Uncategorized Sample	BioSample 1	(F001785)	Phosphatidylethanolamine-binding protein 1 OS=Homo sapiens GN=PEBP1 PE=1 SV=3	PEBP1_HUMAN	SwissProt_2014_08.fasta	21,056.90	100.00%	5	5	6	0.04%	31.00%
Uncategorized Sample	BioSample 1	(F001786)	Proteasome subunit beta type-1 OS=Homo sapiens GN=PSMB1 PE=1 SV=2	PSB1_HUMAN	SwissProt_2014_08.fasta	26,490.50	100.00%	5	6	7	0.04%	26.10%
Uncategorized Sample	BioSample 1	(F001786)	TAR DNA-binding protein 43 OS=Homo sapiens GN=TARDBP PE=1 SV=1	TADBP_HUMAN	SwissProt_2014_08.fasta	44,739.70	100.00%	3	3	25	0.16%	7.97%
Uncategorized Sample	BioSample 1	(F001786)	Myelin-oligodendrocyte glycoprotein OS=Homo sapiens GN=MOG PE=1 SV=2	MOG_HUMAN	SwissProt_2014_08.fasta	28,193.80	100.00%	4	4	4	0.03%	19.80%
Uncategorized Sample	BioSample 1	(F001786)	Prostaglandin-H2 D-isomerase OS=Homo sapiens GN=PTGDS PE=1 SV=1	PTGDS_HUMAN	SwissProt_2014_08.fasta	21,029.10	99.80%	2	2	3	0.02%	12.10%
Uncategorized Sample	BioSample 1	(F001786)	Tubulin beta-4A chain OS=Homo sapiens GN=TUBB4A PE=1 SV=2	TBB4A_HUMAN	SwissProt_2014_08.fasta	49,585.50	99.80%	2	2	2	0.01%	4.50%
Uncategorized Sample	BioSample 1	(F001786)	Ubiquitin carboxyl-terminal hydrolase isozyme L1 OS=Homo sapiens GN=UCHL1 PE=1 SV=2	UCHL1_HUMAN	SwissProt_2014_08.fasta	24,824.30	100.00%	3	3	4	0.03%	18.40%
Uncategorized Sample	BioSample 1	(F001786)	Proteasome subunit beta type-6 OS=Homo sapiens GN=PSMB6 PE=1 SV=4	PSB6_HUMAN	SwissProt_2014_08.fasta	25,357.90	100.00%	3	3	8	0.05%	12.60%
Uncategorized Sample	BioSample 1	(F001786)	Synaptosomal-associated protein 25 OS=Homo sapiens GN=SNAP25 PE=1 SV=1	SNP25_HUMAN	SwissProt_2014_08.fasta	23,315.40	100.00%	9	9	13	0.08%	55.80%
Uncategorized Sample	BioSample 1	(F001786)	Hyaluronan and proteoglycan link protein 2 OS=Homo sapiens GN=HAPLN2 PE=1 SV=1	HPLN2_HUMAN	SwissProt_2014_08.fasta	37,775.60	99.70%	2	2	2	0.01%	5.29%
Uncategorized Sample	BioSample 1	(F001786)	Ras-related protein Rab-5C OS=Homo sapiens GN=RAB5C PE=1 SV=2	RAB5C_HUMAN	SwissProt_2014_08.fasta	23,482.70	100.00%	2	2	2	0.01%	11.10%
Uncategorized Sample	BioSample 1	(F001786)	Keratin, type I cytoskeletal 9 OS=Homo sapiens GN=KRT9 PE=1 SV=3	K1C9_HUMAN	SwissProt_2014_08.fasta	62,065.90	99.90%	2	2	2	0.01%	4.01%
Uncategorized Sample	BioSample 1	(F001786)	Ferritin light chain OS=Homo sapiens GN=FTL PE=1 SV=2	FRIL_HUMAN	SwissProt_2014_08.fasta	20,020.60	100.00%	4	5	43	0.27%	26.30%
Uncategorized Sample	BioSample 1	(F001786)	Peroxiredoxin-1 OS=Homo sapiens GN=PRDX1 PE=1 SV=1	PRDX1_HUMAN	SwissProt_2014_08.fasta	22,110.90	100.00%	5	5	7	0.04%	29.10%
Uncategorized Sample	BioSample 1	(F001786)	Glial fibrillary acidic protein OS=Homo sapiens GN=GFAP PE=1 SV=1	GFAP_HUMAN	SwissProt_2014_08.fasta	49,881.40	100.00%	14	14	18	0.11%	28.00%
Uncategorized Sample	BioSample 1	(F001786)	Microtubule-associated protein tau OS=Homo sapiens GN=MAPT PE=1 SV=5	TAU_HUMAN	SwissProt_2014_08.fasta	78,927.70	100.00%	4	5	6	0.04%	5.01%
Uncategorized Sample	BioSample 1	(F001786)	Glutathione S-transferase P OS=Homo sapiens GN=GSTP1 PE=1 SV=2	GSTP1_HUMAN	SwissProt_2014_08.fasta	23,356.70	99.80%	2	2	2	0.01%	12.90%
Uncategorized Sample	BioSample 1	(F001786)	Rho GDP-dissociation inhibitor 1 OS=Homo sapiens GN=ARHGDIA PE=1 SV=3	GDIR1_HUMAN	SwissProt_2014_08.fasta	23,207.50	100.00%	3	3	4	0.03%	22.50%
Uncategorized Sample	BioSample 1	(F001786)	Flavin reductase (NADPH) OS=Homo sapiens GN=BLVRB PE=1 SV=3	BLVRB_HUMAN	SwissProt_2014_08.fasta	22,118.70	99.80%	2	2	3	0.02%	11.70%
Uncategorized Sample	BioSample 1	(F001786)	Transgelin-3 OS=Homo sapiens GN=TAGLN3 PE=1 SV=2	TAGL3_HUMAN	SwissProt_2014_08.fasta	22,472.90	100.00%	2	2	2	0.01%	11.10%
Uncategorized Sample	BioSample 1	(F001786)	Ras-related protein Rab-14 OS=Homo sapiens GN=RAB14 PE=1 SV=4	RAB14_HUMAN	SwissProt_2014_08.fasta	23,897.60	100.00%	5	5	7	0.04%	25.60%
Uncategorized Sample	BioSample 1	(F001786)	Vesicle-trafficking protein SEC22b OS=Homo sapiens GN=SEC22B PE=1 SV=4	SC22B_HUMAN	SwissProt_2014_08.fasta	24,594.00	99.90%	2	2	2	0.01%	10.20%
Uncategorized Sample	BioSample 1	(F001786)	Neurofilament light polypeptide OS=Homo sapiens GN=NEFL PE=1 SV=3	NFL_HUMAN	SwissProt_2014_08.fasta	61,517.80	100.00%	3	3	3	0.02%	8.84%
Uncategorized Sample	BioSample 1	(F001786)	Ras-related protein Rab-10 OS=Homo sapiens GN=RAB10 PE=1 SV=1	RAB10_HUMAN	SwissProt_2014_08.fasta	22,542.10	99.90%	2	2	3	0.02%	17.00%

Uncategorized Sample	BioSample 1	(F001786)	Triosephosphate isomerase OS=Homo sapiens GN=TPI1 PE=1 SV=3	TPIS_HUMAN	SwissProt_2014_08.fasta	30,790.80	99.90%	2	2	2	0.01%	8.74%
Uncategorized Sample	BioSample 1	(F001786)	Tubulin alpha-1A chain OS=Homo sapiens GN=TUBA1A PE=1 SV=1	TBA1A_HUMAN	SwissProt_2014_08.fasta	50,135.70	100.00%	4	4	4	0.03%	10.40%
Uncategorized Sample	BioSample 1	(F001786)	Peroxiredoxin-2 OS=Homo sapiens GN=PRDX2 PE=1 SV=5	PRDX2_HUMAN	SwissProt_2014_08.fasta	21,892.40	100.00%	7	7	7	0.04%	28.30%
Uncategorized Sample	BioSample 1	(F001786)	Heme-binding protein 1 OS=Homo sapiens GN=HEBP1 PE=1 SV=1	HEBP1_HUMAN	SwissProt_2014_08.fasta	21,097.40	99.90%	2	2	2	0.01%	13.20%
Uncategorized Sample	BioSample 1	(F001786)	Adenylate kinase isoenzyme 1 OS=Homo sapiens GN=AK1 PE=1 SV=3	KAD1_HUMAN	SwissProt_2014_08.fasta	21,635.20	100.00%	3	3	3	0.02%	17.50%
Uncategorized Sample	BioSample 1	(F001786)	Keratin, type I cytoskeletal 10 OS=Homo sapiens GN=KRT10 PE=1 SV=6	K1C10_HUMAN	SwissProt_2014_08.fasta	58,828.80	100.00%	3	3	4	0.03%	5.99%
Uncategorized Sample	BioSample 1	(F001786)	Actin, cytoplasmic 1 OS=Homo sapiens GN=ACTB PE=1 SV=1	ACTB_HUMAN,ACTG_HUMAN	SwissProt_2014_08.fasta	41,737.80	100.00%	3	3	4	0.03%	9.87%
Uncategorized Sample	BioSample 1	(F001786)	Thy-1 membrane glycoprotein OS=Homo sapiens GN=THY1 PE=1 SV=2	THY1_HUMAN	SwissProt_2014_08.fasta	17,935.20	99.80%	2	2	2	0.01%	15.50%
Uncategorized Sample	BioSample 1	(F001786)	Fibrinogen alpha chain OS=Homo sapiens GN=FGA PE=1 SV=2	FIBA_HUMAN	SwissProt_2014_08.fasta	94,973.40	100.00%	4	4	4	0.03%	4.27%
Uncategorized Sample	BioSample 1	(F001786)	Proteasome subunit beta type-4 OS=Homo sapiens GN=PSMB4 PE=1 SV=4	PSB4_HUMAN	SwissProt_2014_08.fasta	29,205.00	100.00%	4	4	5	0.03%	18.90%
Uncategorized Sample	BioSample 1	(F001786)	Proteasome subunit beta type-2 OS=Homo sapiens GN=PSMB2 PE=1 SV=1	PSB2_HUMAN	SwissProt_2014_08.fasta	22,837.50	100.00%	3	3	5	0.03%	20.90%
Uncategorized Sample	BioSample 1	(F001786)	14-3-3 protein gamma OS=Homo sapiens GN=YWHAG PE=1 SV=2	1433G_HUMAN	SwissProt_2014_08.fasta	28,303.10	100.00%	4	4	4	0.03%	13.80%
Uncategorized Sample	BioSample 1	(F001786)	Ras-related protein Rab-6A OS=Homo sapiens GN=RAB6A PE=1 SV=3	RAB6A_HUMAN	SwissProt_2014_08.fasta	23,593.30	100.00%	3	3	5	0.03%	23.10%
Uncategorized Sample	BioSample 1	(F001786)	Myelin basic protein OS=Homo sapiens GN=MBP PE=1 SV=3	MBP_HUMAN	SwissProt_2014_08.fasta	33,117.70	100.00%	4	4	4	0.03%	14.50%
Uncategorized Sample	BioSample 1	(F001786)	Proteasome subunit alpha type-6 OS=Homo sapiens GN=PSMA6 PE=1 SV=1	PSA6_HUMAN	SwissProt_2014_08.fasta	27,399.50	99.80%	2	2	2	0.01%	8.94%
Uncategorized Sample	BioSample 1	(F001786)	Proteasome subunit alpha type-2 OS=Homo sapiens GN=PSMA2 PE=1 SV=2	PSA2_HUMAN	SwissProt_2014_08.fasta	25,898.90	100.00%	5	5	5	0.03%	36.80%
Uncategorized Sample	BioSample 1	(F001786)	Superoxide dismutase [Mn], mitochondrial OS=Homo sapiens GN=SOD2 PE=1 SV=2	SODM_HUMAN	SwissProt_2014_08.fasta	24,722.60	100.00%	3	3	3	0.02%	17.10%
Uncategorized Sample	BioSample 1	(F001786)	ATP synthase subunit O, mitochondrial OS=Homo sapiens GN=ATP5O PE=1 SV=1	ATPO_HUMAN	SwissProt_2014_08.fasta	23,277.40	100.00%	3	3	3	0.02%	16.90%
Uncategorized Sample	BioSample 1	(F001786)	Cell cycle exit and neuronal differentiation protein 1 OS=Homo sapiens GN=CEND1 PE=2 SV=1	CEND_HUMAN	SwissProt_2014_08.fasta	14,954.10	99.90%	2	2	2	0.01%	22.10%
Uncategorized Sample	BioSample 1	(F001786)	Proteasome subunit beta type-5 OS=Homo sapiens GN=PSMB5 PE=1 SV=3	PSB5_HUMAN	SwissProt_2014_08.fasta	28,481.00	100.00%	8	9	12	0.08%	37.30%
Uncategorized Sample	BioSample 1	(F001786)	Proteasome subunit beta type-3 OS=Homo sapiens GN=PSMB3 PE=1 SV=2	PSB3_HUMAN	SwissProt_2014_08.fasta	22,949.60	100.00%	3	4	5	0.03%	23.40%
Uncategorized Sample	BioSample 1	(F001786)	Myelin proteolipid protein OS=Homo sapiens GN=PLP1 PE=1 SV=2	MYPR_HUMAN	SwissProt_2014_08.fasta	30,077.70	100.00%	3	3	16	0.10%	11.60%
Uncategorized Sample	BioSample 1	(F001786)	Apolipoprotein E OS=Homo sapiens GN=APOE PE=1 SV=1	APOE_HUMAN	SwissProt_2014_08.fasta	36,153.50	100.00%	4	4	4	0.03%	13.90%
Uncategorized Sample	BioSample 1	(F001786)	Ubiquitin-60S ribosomal protein L40 OS=Homo sapiens GN=UBA52 PE=1 SV=2	RL40_HUMAN,RS27A_HUMAN,U	SwissProt_2014_08.fasta	14,728.90	99.90%	2	2	2	0.01%	22.70%
Uncategorized Sample	BioSample 1	(F001786)	Neurofilament medium polypeptide OS=Homo sapiens GN=NEFM PE=1 SV=3	NFM_HUMAN	SwissProt_2014_08.fasta	102,471.30	100.00%	3	3	3	0.02%	3.93%
Uncategorized Sample	BioSample 1	(F001786)	Calcium/calmodulin-dependent protein kinase type II subunit alpha OS=Homo sapiens GN=CAMK2A PE=1 SV=2	KCC2A_HUMAN	SwissProt_2014_08.fasta	54,088.70	100.00%	11	11	24	0.15%	25.90%
Uncategorized Sample	BioSample 1	(F001786)	Keratin, type II cytoskeletal 1 OS=Homo sapiens GN=KRT1 PE=1 SV=6	K2C1_HUMAN	SwissProt_2014_08.fasta	66,040.30	100.00%	5	5	5	0.03%	11.30%
Uncategorized Sample	BioSample 1	(F001786)	Ferritin heavy chain OS=Homo sapiens GN=FTH1 PE=1 SV=2	FRIH_HUMAN	SwissProt_2014_08.fasta	21,226.20	100.00%	5	5	5	0.03%	25.10%
Uncategorized Sample	BioSample 1	(F001788)	Ubiquitin thioesterase OTUB1 OS=Homo sapiens GN=OTUB1 PE=1 SV=2	OTUB1_HUMAN	SwissProt_2014_08.fasta	31,284.60	100.00%	2	2	2	0.01%	9.96%
Uncategorized Sample	BioSample 1	(F001788)	TAR DNA-binding protein 43 OS=Homo sapiens GN=TARDBP PE=1 SV=1	TADBP_HUMAN	SwissProt_2014_08.fasta	44,739.70	100.00%	3	4	8	0.05%	9.42%
Uncategorized Sample	BioSample 1	(F001788)	Alpha-internexin OS=Homo sapiens GN=INA PE=1 SV=2	AINX_HUMAN	SwissProt_2014_08.fasta	55,392.30	100.00%	5	5	6	0.04%	13.20%
Uncategorized Sample	BioSample 1	(F001788)	Glyoxylate reductase/hydroxypyruvate reductase OS=Homo sapiens GN=GRHPR PE=1 SV=1	GRHPR_HUMAN	SwissProt_2014_08.fasta	35,669.10	100.00%	2	2	2	0.01%	10.40%
Uncategorized Sample	BioSample 1	(F001788)	Protein FAM49B OS=Homo sapiens GN=FAM49B PE=1 SV=1	FA49B_HUMAN	SwissProt_2014_08.fasta	36,748.70	100.00%	2	2	2	0.01%	8.02%
Uncategorized Sample	BioSample 1	(F001788)	Glyceraldehyde-3-phosphate dehydrogenase OS=Homo sapiens GN=GAPDH PE=1 SV=3	G3P_HUMAN	SwissProt_2014_08.fasta	36,053.40	99.90%	2	2	2	0.01%	11.30%
Uncategorized Sample	BioSample 1	(F001788)	Tubulin beta-4A chain OS=Homo sapiens GN=TUBB4A PE=1 SV=2	TBB4A_HUMAN	SwissProt_2014_08.fasta	49,585.50	100.00%	18	22	48	0.28%	46.20%
Uncategorized Sample	BioSample 1	(F001788)	Alpha-soluble NSF attachment protein OS=Homo sapiens GN=NAPA PE=1 SV=3	SNAA_HUMAN	SwissProt_2014_08.fasta	33,234.10	99.90%	2	2	2	0.01%	21.00%
Uncategorized Sample	BioSample 1	(F001788)	Guanine nucleotide-binding protein G(o) subunit alpha OS=Homo sapiens GN=GNAO1 PE=1 SV=4	GNAO_HUMAN	SwissProt_2014_08.fasta	40,052.00	100.00%	4	4	7	0.04%	13.60%
Uncategorized Sample	BioSample 1	(F001788)	Histone H1.3 OS=Homo sapiens GN=HIST1H1D PE=1 SV=2	H13_HUMAN	SwissProt_2014_08.fasta	22,351.30	100.00%	4	4	20	0.12%	15.40%
Uncategorized Sample	BioSample 1	(F001788)	Malate dehydrogenase, cytoplasmic OS=Homo sapiens GN=MDH1 PE=1 SV=4	MDHC_HUMAN	SwissProt_2014_08.fasta	36,426.90	100.00%	7	7	12	0.07%	28.40%
Uncategorized Sample	BioSample 1	(F001788)	Calcium/calmodulin-dependent protein kinase type II subunit delta OS=Homo sapiens GN=CAMK2D PE=1 SV=3	KCC2D_HUMAN	SwissProt_2014_08.fasta	56,370.70	100.00%	3	3	5	0.03%	15.20%
Uncategorized Sample	BioSample 1	(F001788)	Calcium/calmodulin-dependent protein kinase type II subunit beta OS=Homo sapiens GN=CAMK2B PE=1 SV=3	KCC2B_HUMAN	SwissProt_2014_08.fasta	72,678.70	100.00%	5	5	9	0.05%	17.60%
Uncategorized Sample	BioSample 1	(F001788)	Guanine nucleotide-binding protein G(I)/G(S)/G(T) subunit beta-1 OS=Homo sapiens GN=GNB1 PE=1 SV=3	GBB1_HUMAN	SwissProt_2014_08.fasta	37,377.50	100.00%	2	2	6	0.04%	6.18%
Uncategorized Sample	BioSample 1	(F001788)	V-type proton ATPase subunit D OS=Homo sapiens GN=ATP6V1D PE=1 SV=1	VATD_HUMAN	SwissProt_2014_08.fasta	28,264.30	99.80%	2	2	2	0.01%	11.30%
Uncategorized Sample	BioSample 1	(F001788)	Voltage-dependent anion-selective channel protein 2 OS=Homo sapiens GN=VDAC2 PE=1 SV=2	VDAC2_HUMAN	SwissProt_2014_08.fasta	31,567.30	100.00%	2	2	2	0.01%	11.20%
Uncategorized Sample	BioSample 1	(F001788)	Heterogeneous nuclear ribonucleoprotein A3 OS=Homo sapiens GN=HNRNPA3 PE=1 SV=2	ROA3_HUMAN	SwissProt_2014_08.fasta	39,595.10	99.80%	2	2	3	0.02%	7.14%
Uncategorized Sample	BioSample 1	(F001788)	Heterogeneous nuclear ribonucleoprotein A1 OS=Homo sapiens GN=HNRNPA1 PE=1 SV=5	ROA1_HUMAN	SwissProt_2014_08.fasta	38,747.00	100.00%	6	6	10	0.06%	23.70%
Uncategorized Sample	BioSample 1	(F001788)	Complement C4-A OS=Homo sapiens GN=C4A PE=1 SV=2	CO4A_HUMAN	SwissProt_2014_08.fasta	192,786.80	100.00%	4	4	4	0.02%	4.30%
Uncategorized Sample	BioSample 1	(F001788)	Tubulin beta-2A chain OS=Homo sapiens GN=TUBB2A PE=1 SV=1	TBB2A_HUMAN	SwissProt_2014_08.fasta	49,907.10	100.00%	5	5	9	0.05%	41.30%
Uncategorized Sample	BioSample 1	(F001788)	Hyaluronan and proteoglycan link protein 2 OS=Homo sapiens GN=HAPLN2 PE=1 SV=1	HPLN2_HUMAN	SwissProt_2014_08.fasta	37,775.60	100.00%	3	3	6	0.04%	7.94%
Uncategorized Sample	BioSample 1	(F001788)	Syntaxin-1B OS=Homo sapiens GN=STX1B PE=1 SV=1	STX1B_HUMAN	SwissProt_2014_08.fasta	33,245.10	100.00%	6	7	12	0.07%	23.60%
Uncategorized Sample	BioSample 1	(F001788)	Tubulin beta-4B chain OS=Homo sapiens GN=TUBB4B PE=1 SV=1	TBB4B_HUMAN	SwissProt_2014_08.fasta	49,830.70	100.00%	6	6	17	0.10%	41.30%
Uncategorized Sample	BioSample 1	(F001788)	Ferritin light chain OS=Homo sapiens GN=FTL PE=1 SV=2	FRIL_HUMAN	SwissProt_2014_08.fasta	20,020.60	99.90%	2	2	3	0.02%	17.10%
Uncategorized Sample	BioSample 1	(F001788)	2',3'-cyclic-nucleotide 3'-phosphodiesterase OS=Homo sapiens GN=CNP PE=1 SV=2	CN37_HUMAN	SwissProt_2014_08.fasta	47,580.60	100.00%	7	7	8	0.05%	20.70%
Uncategorized Sample	BioSample 1	(F001788)	Annexin A5 OS=Homo sapiens GN=ANXA5 PE=1 SV=2	ANXA5_HUMAN	SwissProt_2014_08.fasta	35,938.60	100.00%	4	4	4	0.02%	15.00%
Uncategorized Sample	BioSample 1	(F001788)	Heterogeneous nuclear ribonucleoproteins A2/B1 OS=Homo sapiens GN=HNRNPA2B1 PE=1 SV=2	ROA2_HUMAN	SwissProt_2014_08.fasta	37,430.30	100.00%	13	14	57	0.33%	49.90%
Uncategorized Sample	BioSample 1	(F001788)	Heterogeneous nuclear ribonucleoprotein A0 OS=Homo sapiens GN=HNRNPA0 PE=1 SV=1	ROA0_HUMAN	SwissProt_2014_08.fasta	30,840.50	99.80%	2	2	2	0.01%	11.80%
Uncategorized Sample	BioSample 1	(F001788)	Glial fibrillary acidic protein OS=Homo sapiens GN=GFAP PE=1 SV=1	GFAP_HUMAN	SwissProt_2014_08.fasta	49,881.40	100.00%	5	5	11	0.06%	12.00%
Uncategorized Sample	BioSample 1	(F001788)	L-lactate dehydrogenase A chain OS=Homo sapiens GN=LDHA PE=1 SV=2	LDHA_HUMAN	SwissProt_2014_08.fasta	36,689.20	100.00%	7	7	9	0.05%	26.80%

Uncategorized Sample	BioSample 1	(F001788)	Syntaxin-1A OS=Homo sapiens GN=STX1A PE=1 SV=1	STX1A_HUMAN	SwissProt_2014_08.fasta	33,024.30	100.00%	4	4	5	0.03%	20.10%
Uncategorized Sample	BioSample 1	(F001788)	Heterogeneous nuclear ribonucleoprotein H3 OS=Homo sapiens GN=HNRNPH3 PE=1 SV=2	HNRH3_HUMAN	SwissProt_2014_08.fasta	36,927.60	100.00%	3	3	4	0.02%	14.50%
Uncategorized Sample	BioSample 1	(F001788)	Malate dehydrogenase, mitochondrial OS=Homo sapiens GN=MDH2 PE=1 SV=3	MDHM_HUMAN	SwissProt_2014_08.fasta	35,503.70	100.00%	7	7	10	0.06%	30.50%
Uncategorized Sample	BioSample 1	(F001788)	Neurofilament light polypeptide OS=Homo sapiens GN=NEFL PE=1 SV=3	NFL_HUMAN	SwissProt_2014_08.fasta	61,517.80	100.00%	6	6	7	0.04%	12.30%
Uncategorized Sample	BioSample 1	(F001788)	Tubulin alpha-4A chain OS=Homo sapiens GN=TUBA4A PE=1 SV=1	TBA4A_HUMAN	SwissProt_2014_08.fasta	49,924.60	99.80%	2	2	2	0.01%	34.80%
Uncategorized Sample	BioSample 1	(F001788)	Pyruvate dehydrogenase E1 component subunit beta, mitochondrial OS=Homo sapiens GN=PDHB PE=1 SV=3	ODPB_HUMAN	SwissProt_2014_08.fasta	39,233.40	100.00%	2	2	3	0.02%	8.91%
Uncategorized Sample	BioSample 1	(F001788)	Charged multivesicular body protein 4b OS=Homo sapiens GN=CHMP4B PE=1 SV=1	CHM4B_HUMAN	SwissProt_2014_08.fasta	24,951.20	100.00%	5	5	13	0.08%	34.80%
Uncategorized Sample	BioSample 1	(F001788)	Versican core protein OS=Homo sapiens GN=VCAN PE=1 SV=3	CSPG2_HUMAN	SwissProt_2014_08.fasta	372,812.60	100.00%	2	2	2	0.01%	0.91%
Uncategorized Sample	BioSample 1	(F001788)	Phytanoyl-CoA hydroxylase-interacting protein OS=Homo sapiens GN=PHYHIP PE=1 SV=1	PHYIP_HUMAN	SwissProt_2014_08.fasta	37,573.20	99.80%	2	2	3	0.02%	7.27%
Uncategorized Sample	BioSample 1	(F001788)	Sideroflexin-3 OS=Homo sapiens GN=SFXN3 PE=1 SV=2	SFXN3_HUMAN	SwissProt_2014_08.fasta	35,979.10	100.00%	3	3	3	0.02%	13.20%
Uncategorized Sample	BioSample 1	(F001788)	Tubulin beta chain OS=Homo sapiens GN=TUBB PE=1 SV=2	TBB5_HUMAN	SwissProt_2014_08.fasta	49,670.60	100.00%	3	3	3	0.02%	41.20%
Uncategorized Sample	BioSample 1	(F001788)	Prohibitin-2 OS=Homo sapiens GN=PHB2 PE=1 SV=2	PHB2_HUMAN	SwissProt_2014_08.fasta	33,297.60	100.00%	4	4	4	0.02%	16.10%
Uncategorized Sample	BioSample 1	(F001788)	Creatine kinase B-type OS=Homo sapiens GN=CKB PE=1 SV=1	KCRB_HUMAN	SwissProt_2014_08.fasta	42,645.10	100.00%	6	7	16	0.09%	20.50%
Uncategorized Sample	BioSample 1	(F001788)	Actin, cytoplasmic 1 OS=Homo sapiens GN=ACTB PE=1 SV=1	ACTB_HUMAN,ACTG_HUMAN	SwissProt_2014_08.fasta	41,737.80	100.00%	3	3	5	0.03%	9.60%
Uncategorized Sample	BioSample 1	(F001788)	Tubulin alpha-1A chain OS=Homo sapiens GN=TUBA1A PE=1 SV=1	TBA1A_HUMAN	SwissProt_2014_08.fasta	50,135.70	100.00%	12	16	23	0.14%	41.00%
Uncategorized Sample	BioSample 1	(F001788)	Clusterin OS=Homo sapiens GN=CLU PE=1 SV=1	CLUS_HUMAN	SwissProt_2014_08.fasta	52,495.00	100.00%	5	5	11	0.06%	16.30%
Uncategorized Sample	BioSample 1	(F001788)	L-lactate dehydrogenase B chain OS=Homo sapiens GN=LDHB PE=1 SV=2	LDHB_HUMAN	SwissProt_2014_08.fasta	36,638.60	100.00%	7	9	27	0.16%	25.40%
Uncategorized Sample	BioSample 1	(F001788)	Proteasome subunit alpha type-2 OS=Homo sapiens GN=PSMA2 PE=1 SV=2	PSA2_HUMAN	SwissProt_2014_08.fasta	25,898.90	99.80%	2	2	2	0.01%	17.10%
Uncategorized Sample	BioSample 1	(F001788)	Carbonyl reductase [NADPH] 1 OS=Homo sapiens GN=CBR1 PE=1 SV=3	CBR1_HUMAN	SwissProt_2014_08.fasta	30,374.80	100.00%	8	9	13	0.08%	37.90%
Uncategorized Sample	BioSample 1	(F001788)	ATP synthase subunit gamma, mitochondrial OS=Homo sapiens GN=ATP5C1 PE=1 SV=1	ATPG_HUMAN	SwissProt_2014_08.fasta	32,998.00	100.00%	2	2	2	0.01%	8.39%
Uncategorized Sample	BioSample 1	(F001788)	Microtubule-associated protein RP/EB family member 2 OS=Homo sapiens GN=MAPRE2 PE=1 SV=1	MARE2_HUMAN	SwissProt_2014_08.fasta	37,031.60	100.00%	2	2	2	0.01%	11.30%
Uncategorized Sample	BioSample 1	(F001788)	Acyl-coenzyme A thioesterase 8 OS=Homo sapiens GN=ACOT8 PE=1 SV=1	ACOT8_HUMAN	SwissProt_2014_08.fasta	35,913.40	100.00%	2	2	2	0.01%	10.00%
Uncategorized Sample	BioSample 1	(F001788)	Beta-soluble NSF attachment protein OS=Homo sapiens GN=NAPB PE=1 SV=2	SNAB_HUMAN	SwissProt_2014_08.fasta	33,558.20	100.00%	6	6	7	0.04%	29.50%
Uncategorized Sample	BioSample 1	(F001788)	NAD-dependent protein deacetylase sirtuin-2 OS=Homo sapiens GN=SIRT2 PE=1 SV=2	SIR2_HUMAN	SwissProt_2014_08.fasta	43,183.70	100.00%	4	4	5	0.03%	15.40%
Uncategorized Sample	BioSample 1	(F001788)	Serine/threonine-protein phosphatase 2A catalytic subunit alpha isoform OS=Homo sapiens GN=PPP2CA PE=1 S	PP2AA_HUMAN,PP2AB_HUMAN	SwissProt_2014_08.fasta	35,594.50	99.80%	2	2	2	0.01%	8.41%
Uncategorized Sample	BioSample 1	(F001788)	Ubiquitin-60S ribosomal protein L40 OS=Homo sapiens GN=UBA52 PE=1 SV=2	RL40_HUMAN,RS27A_HUMAN,U	SwissProt_2014_08.fasta	14,728.90	100.00%	2	2	3	0.02%	22.70%
Uncategorized Sample	BioSample 1	(F001788)	Neurofilament medium polypeptide OS=Homo sapiens GN=NEFM PE=1 SV=3	NFM_HUMAN	SwissProt_2014_08.fasta	102,471.30	100.00%	3	3	3	0.02%	5.57%
Uncategorized Sample	BioSample 1	(F001788)	Apolipoprotein E OS=Homo sapiens GN=APOE PE=1 SV=1	APOE_HUMAN	SwissProt_2014_08.fasta	36,153.50	100.00%	10	10	18	0.11%	39.70%
Uncategorized Sample	BioSample 1	(F001788)	Calcium/calmodulin-dependent protein kinase type II subunit alpha OS=Homo sapiens GN=CAMK2A PE=1 SV=2	KCC2A_HUMAN	SwissProt_2014_08.fasta	54,088.70	100.00%	10	10	20	0.12%	24.30%
Uncategorized Sample	BioSample 1	(F001788)	Keratin, type II cytoskeletal 1 OS=Homo sapiens GN=KRT1 PE=1 SV=6	K2C1_HUMAN	SwissProt_2014_08.fasta	66,040.30	100.00%	5	5	5	0.03%	9.63%
Uncategorized Sample	BioSample 1	(F001788)	Voltage-dependent anion-selective channel protein 1 OS=Homo sapiens GN=VDAC1 PE=1 SV=2	VDAC1_HUMAN	SwissProt_2014_08.fasta	30,773.90	100.00%	11	11	41	0.24%	48.80%
Uncategorized Sample	BioSample 1	(F001789)	TAR DNA-binding protein 43 OS=Homo sapiens GN=TARDBP PE=1 SV=1	TADBP_HUMAN	SwissProt_2014_08.fasta	44,739.70	100.00%	7	8	11	0.06%	21.00%
Uncategorized Sample	BioSample 1	(F001789)	Isocitrate dehydrogenase [NADP], mitochondrial OS=Homo sapiens GN=IDH2 PE=1 SV=2	IDHP_HUMAN	SwissProt_2014_08.fasta	50,910.40	100.00%	4	4	7	0.04%	12.20%
Uncategorized Sample	BioSample 1	(F001789)	Actin, alpha cardiac muscle 1 OS=Homo sapiens GN=ACTC1 PE=1 SV=1	ACTC_HUMAN,ACTS_HUMAN	SwissProt_2014_08.fasta	42,020.10	100.00%	2	2	2	0.01%	30.80%
Uncategorized Sample	BioSample 1	(F001789)	Adenosylhomocysteinase OS=Homo sapiens GN=AHCY PE=1 SV=4	SAHH_HUMAN	SwissProt_2014_08.fasta	47,717.10	100.00%	4	4	4	0.02%	9.49%
Uncategorized Sample	BioSample 1	(F001789)	Glutamine synthetase OS=Homo sapiens GN=GLUL PE=1 SV=4	GLNA_HUMAN	SwissProt_2014_08.fasta	42,064.90	100.00%	2	2	2	0.01%	7.51%
Uncategorized Sample	BioSample 1	(F001789)	Tubulin beta-4A chain OS=Homo sapiens GN=TUBB4A PE=1 SV=2	TBB4A_HUMAN	SwissProt_2014_08.fasta	49,585.50	100.00%	10	10	13	0.07%	32.40%
Uncategorized Sample	BioSample 1	(F001789)	Neurofilament heavy polypeptide OS=Homo sapiens GN=NEFH PE=1 SV=4	NFH_HUMAN	SwissProt_2014_08.fasta	112,480.10	100.00%	3	3	3	0.02%	4.09%
Uncategorized Sample	BioSample 1	(F001789)	Neuromodulin OS=Homo sapiens GN=GAP43 PE=1 SV=1	NEUM_HUMAN	SwissProt_2014_08.fasta	24,802.40	100.00%	3	3	4	0.02%	30.70%
Uncategorized Sample	BioSample 1	(F001789)	Calcium/calmodulin-dependent protein kinase type II subunit beta OS=Homo sapiens GN=CAMK2B PE=1 SV=3	KCC2B_HUMAN	SwissProt_2014_08.fasta	72,678.70	100.00%	2	2	3	0.02%	8.86%
Uncategorized Sample	BioSample 1	(F001789)	cAMP-dependent protein kinase catalytic subunit beta OS=Homo sapiens GN=PRKACB PE=1 SV=2	KAPCB_HUMAN	SwissProt_2014_08.fasta	40,624.40	100.00%	3	3	3	0.02%	10.80%
Uncategorized Sample	BioSample 1	(F001789)	Heterogeneous nuclear ribonucleoprotein D0 OS=Homo sapiens GN=HNRNPD PE=1 SV=1	HNRPD_HUMAN	SwissProt_2014_08.fasta	38,434.50	100.00%	4	4	8	0.05%	16.60%
Uncategorized Sample	BioSample 1	(F001789)	Phosphoglycerate kinase 1 OS=Homo sapiens GN=PGK1 PE=1 SV=3	PGK1_HUMAN	SwissProt_2014_08.fasta	44,615.30	100.00%	3	4	6	0.03%	9.11%
Uncategorized Sample	BioSample 1	(F001789)	Complement C4-A OS=Homo sapiens GN=C4A PE=1 SV=2	CO4A_HUMAN	SwissProt_2014_08.fasta	192,786.80	100.00%	3	3	3	0.02%	2.81%
Uncategorized Sample	BioSample 1	(F001789)	Creatine kinase U-type, mitochondrial OS=Homo sapiens GN=CKMT1A PE=1 SV=1	KCRU_HUMAN	SwissProt_2014_08.fasta	47,038.00	100.00%	6	6	10	0.06%	19.70%
Uncategorized Sample	BioSample 1	(F001789)	Fructose-bisphosphate aldolase C OS=Homo sapiens GN=ALDOC PE=1 SV=2	ALDOC_HUMAN	SwissProt_2014_08.fasta	39,456.20	100.00%	3	3	4	0.02%	11.30%
Uncategorized Sample	BioSample 1	(F001789)	Fructose-bisphosphate aldolase A OS=Homo sapiens GN=ALDOA PE=1 SV=2	ALDOA_HUMAN	SwissProt_2014_08.fasta	39,420.60	99.80%	2	2	2	0.01%	8.79%
Uncategorized Sample	BioSample 1	(F001789)	Alpha-centractin OS=Homo sapiens GN=ACTR1A PE=1 SV=1	ACTZ_HUMAN	SwissProt_2014_08.fasta	42,615.20	99.80%	2	2	2	0.01%	9.84%
Uncategorized Sample	BioSample 1	(F001789)	Erlin-2 OS=Homo sapiens GN=ERLIN2 PE=1 SV=1	ERLN2_HUMAN	SwissProt_2014_08.fasta	37,840.40	100.00%	4	4	5	0.03%	15.00%
Uncategorized Sample	BioSample 1	(F001789)	Keratin, type I cytoskeletal 9 OS=Homo sapiens GN=KRT9 PE=1 SV=3	K1C9_HUMAN	SwissProt_2014_08.fasta	62,065.90	99.80%	2	2	2	0.01%	5.78%
Uncategorized Sample	BioSample 1	(F001789)	Actin, cytoplasmic 1 OS=Homo sapiens GN=ACTB PE=1 SV=1	ACTB_HUMAN,ACTG_HUMAN	SwissProt_2014_08.fasta	41,737.80	100.00%	15	20	127	0.73%	48.80%
Uncategorized Sample	BioSample 1	(F001789)	Flotillin-2 OS=Homo sapiens GN=FLOT2 PE=1 SV=2	FLOT2_HUMAN	SwissProt_2014_08.fasta	47,063.90	100.00%	3	3	3	0.02%	8.64%
Uncategorized Sample	BioSample 1	(F001789)	Tubulin beta-4B chain OS=Homo sapiens GN=TUBB4B PE=1 SV=1	TBB4B_HUMAN	SwissProt_2014_08.fasta	49,830.70	100.00%	4	4	5	0.03%	32.40%
Uncategorized Sample	BioSample 1	(F001789)	Ferritin light chain OS=Homo sapiens GN=FTL PE=1 SV=2	FRIL_HUMAN	SwissProt_2014_08.fasta	20,020.60	100.00%	3	3	3	0.02%	17.70%
Uncategorized Sample	BioSample 1	(F001789)	Endophilin-A1 OS=Homo sapiens GN=SH3GL2 PE=1 SV=1	SH3G2_HUMAN	SwissProt_2014_08.fasta	39,963.20	100.00%	2	2	2	0.01%	7.95%
Uncategorized Sample	BioSample 1	(F001789)	2',3'-cyclic-nucleotide 3'-phosphodiesterase OS=Homo sapiens GN=CNP PE=1 SV=2	CN37_HUMAN	SwissProt_2014_08.fasta	47,580.60	100.00%	23	27	87	0.50%	48.70%
Uncategorized Sample	BioSample 1	(F001789)	Heterogeneous nuclear ribonucleoproteins A2/B1 OS=Homo sapiens GN=HNRNPA2B1 PE=1 SV=2	ROA2_HUMAN	SwissProt_2014_08.fasta	37,430.30	100.00%	3	3	3	0.02%	9.63%
Uncategorized Sample	BioSample 1	(F001789)	Glial fibrillary acidic protein OS=Homo sapiens GN=GFAP PE=1 SV=1	GFAP_HUMAN	SwissProt_2014_08.fasta	49,881.40	100.00%	29	33	258	1.49%	63.90%

Uncategorized Sample	BioSample 1	(F001789)	RNA-binding motif protein, X chromosome OS=Homo sapiens GN=RBMX PE=1 SV=3	RBMX_HUMAN	SwissProt_2014_08.fasta	42,333.70	100.00%	2	2	2	0.01%	6.91%
Uncategorized Sample	BioSample 1	(F001789)	Microtubule-associated protein tau OS=Homo sapiens GN=MAPT PE=1 SV=5	TAU_HUMAN	SwissProt_2014_08.fasta	78,927.70	99.80%	2	2	3	0.02%	3.83%
Uncategorized Sample	BioSample 1	(F001789)	ATP synthase subunit alpha, mitochondrial OS=Homo sapiens GN=ATP5A1 PE=1 SV=1	ATPA_HUMAN	SwissProt_2014_08.fasta	59,752.10	100.00%	3	3	3	0.02%	7.05%
Uncategorized Sample	BioSample 1	(F001789)	Keratin, type II cytoskeletal 2 epidermal OS=Homo sapiens GN=KRT2 PE=1 SV=2	K22E_HUMAN	SwissProt_2014_08.fasta	65,433.90	99.80%	2	2	2	0.01%	7.67%
Uncategorized Sample	BioSample 1	(F001789)	Flotillin-1 OS=Homo sapiens GN=FLOT1 PE=1 SV=3	FLOT1_HUMAN	SwissProt_2014_08.fasta	47,354.80	100.00%	2	2	2	0.01%	5.39%
Uncategorized Sample	BioSample 1	(F001789)	Neurofilament light polypeptide OS=Homo sapiens GN=NEFL PE=1 SV=3	NFL_HUMAN	SwissProt_2014_08.fasta	61,517.80	100.00%	19	19	34	0.20%	50.30%
Uncategorized Sample	BioSample 1	(F001789)	Heterogeneous nuclear ribonucleoprotein M OS=Homo sapiens GN=HNRNPM PE=1 SV=3	HNRPM_HUMAN	SwissProt_2014_08.fasta	77,517.30	100.00%	2	2	2	0.01%	2.47%
Uncategorized Sample	BioSample 1	(F001789)	Band 4.1-like protein 3 OS=Homo sapiens GN=EPB41L3 PE=1 SV=2	E41L3_HUMAN	SwissProt_2014_08.fasta	120,679.80	100.00%	3	3	3	0.02%	3.96%
Uncategorized Sample	BioSample 1	(F001789)	Versican core protein OS=Homo sapiens GN=VCAN PE=1 SV=3	CSPG2_HUMAN	SwissProt_2014_08.fasta	372,812.60	100.00%	3	3	4	0.02%	1.21%
Uncategorized Sample	BioSample 1	(F001789)	Heterogeneous nuclear ribonucleoprotein K OS=Homo sapiens GN=HNRNPK PE=1 SV=1	HNRPK_HUMAN	SwissProt_2014_08.fasta	50,978.50	99.80%	2	2	2	0.01%	3.89%
Uncategorized Sample	BioSample 1	(F001789)	Alpha-internexin OS=Homo sapiens GN=INA PE=1 SV=2	AINX_HUMAN	SwissProt_2014_08.fasta	55,392.30	100.00%	11	11	16	0.09%	26.50%
Uncategorized Sample	BioSample 1	(F001789)	Creatine kinase B-type OS=Homo sapiens GN=CKB PE=1 SV=1	KCRB_HUMAN	SwissProt_2014_08.fasta	42,645.10	100.00%	14	19	80	0.46%	50.90%
Uncategorized Sample	BioSample 1	(F001789)	DnaJ homolog subfamily B member 2 OS=Homo sapiens GN=DNAJB2 PE=1 SV=3	DNJB2_HUMAN	SwissProt_2014_08.fasta	35,580.70	100.00%	4	4	4	0.02%	15.40%
Uncategorized Sample	BioSample 1	(F001789)	Heterogeneous nuclear ribonucleoprotein H OS=Homo sapiens GN=HNRNPH1 PE=1 SV=4	HNRH1_HUMAN,HNRH2_HUMAN	SwissProt_2014_08.fasta	49,229.80	99.80%	2	2	2	0.01%	7.35%
Uncategorized Sample	BioSample 1	(F001789)	Brain acid soluble protein 1 OS=Homo sapiens GN=BASP1 PE=1 SV=2	BASP1_HUMAN	SwissProt_2014_08.fasta	22,693.30	99.80%	2	2	2	0.01%	22.90%
Uncategorized Sample	BioSample 1	(F001789)	Tubulin alpha-1A chain OS=Homo sapiens GN=TUBA1A PE=1 SV=1	TBA1A_HUMAN	SwissProt_2014_08.fasta	50,135.70	100.00%	5	5	6	0.03%	14.90%
Uncategorized Sample	BioSample 1	(F001789)	Arf-GAP with GTPase, ANK repeat and PH domain-containing protein 3 OS=Homo sapiens GN=AGAP3 PE=1 SV	AGAP3_HUMAN	SwissProt_2014_08.fasta	95,045.00	99.90%	2	2	2	0.01%	3.20%
Uncategorized Sample	BioSample 1	(F001789)	Succinyl-CoA ligase [ADP-forming] subunit beta, mitochondrial OS=Homo sapiens GN=SUCLA2 PE=1 SV=3	SUCB1_HUMAN	SwissProt_2014_08.fasta	50,318.10	100.00%	2	2	2	0.01%	4.32%
Uncategorized Sample	BioSample 1	(F001789)	Septin-2 OS=Homo sapiens GN=SEPT2 PE=1 SV=1	SEPT2_HUMAN	SwissProt_2014_08.fasta	41,488.20	100.00%	5	5	5	0.03%	21.60%
Uncategorized Sample	BioSample 1	(F001789)	Keratin, type II cytoskeletal 1 OS=Homo sapiens GN=KRT1 PE=1 SV=6	K2C1_HUMAN	SwissProt_2014_08.fasta	66,040.30	100.00%	3	3	3	0.02%	7.61%
Uncategorized Sample	BioSample 1	(F001789)	Calcium/calmodulin-dependent protein kinase type II subunit alpha OS=Homo sapiens GN=CAMK2A PE=1 SV=2	KCC2A_HUMAN	SwissProt_2014_08.fasta	54,088.70	100.00%	9	10	18	0.10%	23.60%
Uncategorized Sample	BioSample 1	(F001789)	Dihydropyrimidinase-related protein 2 OS=Homo sapiens GN=DPYSL2 PE=1 SV=1	DPYL2_HUMAN	SwissProt_2014_08.fasta	62,294.00	99.80%	2	2	3	0.02%	3.67%
Uncategorized Sample	BioSample 1	(F001789)	Myelin proteolipid protein OS=Homo sapiens GN=PLP1 PE=1 SV=2	MYPR_HUMAN	SwissProt_2014_08.fasta	30,077.70	99.80%	2	2	2	0.01%	8.66%
Uncategorized Sample	BioSample 1	(F001789)	Vimentin OS=Homo sapiens GN=VIM PE=1 SV=4	VIME_HUMAN	SwissProt_2014_08.fasta	53,652.70	100.00%	3	3	3	0.02%	8.15%
Uncategorized Sample	BioSample 1	(F001789)	Neurofilament medium polypeptide OS=Homo sapiens GN=NEFM PE=1 SV=3	NFM_HUMAN	SwissProt_2014_08.fasta	102,471.30	100.00%	12	12	17	0.10%	16.20%
Uncategorized Sample	BioSample 1	(F001789)	Ferritin heavy chain OS=Homo sapiens GN=FTH1 PE=1 SV=2	FRIH_HUMAN	SwissProt_2014_08.fasta	21,226.20	99.80%	2	2	3	0.02%	10.90%
Uncategorized Sample	BioSample 1	(F001789)	Elongation factor Tu, mitochondrial OS=Homo sapiens GN=TUFM PE=1 SV=2	EFTU_HUMAN	SwissProt_2014_08.fasta	49,542.40	100.00%	10	12	21	0.12%	25.40%
Uncategorized Sample	BioSample 1	(F001790)	TAR DNA-binding protein 43 OS=Homo sapiens GN=TARDBP PE=1 SV=1	TADBP_HUMAN	SwissProt_2014_08.fasta	44,739.70	100.00%	5	5	11	0.06%	15.90%
Uncategorized Sample	BioSample 1	(F001790)	Alpha-internexin OS=Homo sapiens GN=INA PE=1 SV=2	AINX_HUMAN	SwissProt_2014_08.fasta	55,392.30	100.00%	14	14	18	0.10%	37.90%
Uncategorized Sample	BioSample 1	(F001790)	Tubulin beta-4A chain OS=Homo sapiens GN=TUBB4A PE=1 SV=2	TBB4A_HUMAN	SwissProt_2014_08.fasta	49,585.50	100.00%	10	10	15	0.09%	32.40%
Uncategorized Sample	BioSample 1	(F001790)	Neuromodulin OS=Homo sapiens GN=GAP43 PE=1 SV=1	NEUM_HUMAN	SwissProt_2014_08.fasta	24,802.40	100.00%	6	6	21	0.12%	47.50%
Uncategorized Sample	BioSample 1	(F001790)	Sodium/potassium-transporting ATPase subunit beta-1 OS=Homo sapiens GN=ATP1B1 PE=1 SV=1	AT1B1_HUMAN	SwissProt_2014_08.fasta	35,062.90	99.80%	2	2	3	0.02%	7.26%
Uncategorized Sample	BioSample 1	(F001790)	Elongation factor 1-alpha 1 OS=Homo sapiens GN=EEF1A1 PE=1 SV=1	EF1A1_HUMAN,EF1A3_HUMAN	SwissProt_2014_08.fasta	50,141.20	100.00%	3	3	4	0.02%	9.96%
Uncategorized Sample	BioSample 1	(F001790)	Complement C4-A OS=Homo sapiens GN=C4A PE=1 SV=2	CO4A_HUMAN	SwissProt_2014_08.fasta	192,786.80	99.90%	2	2	2	0.01%	1.38%
Uncategorized Sample	BioSample 1	(F001790)	Neurofilament light polypeptide OS=Homo sapiens GN=NEFL PE=1 SV=3	NFL_HUMAN	SwissProt_2014_08.fasta	61,517.80	100.00%	12	12	52	0.30%	28.70%
Uncategorized Sample	BioSample 1	(F001790)	Tubulin beta-2A chain OS=Homo sapiens GN=TUBB2A PE=1 SV=1	TBB2A_HUMAN	SwissProt_2014_08.fasta	49,907.10	99.80%	2	2	3	0.02%	21.30%
Uncategorized Sample	BioSample 1	(F001790)	Eukaryotic initiation factor 4A-II OS=Homo sapiens GN=EIF4A2 PE=1 SV=2	IF4A2_HUMAN	SwissProt_2014_08.fasta	46,403.60	100.00%	4	4	4	0.02%	13.30%
Uncategorized Sample	BioSample 1	(F001790)	Peroxisomal multifunctional enzyme type 2 OS=Homo sapiens GN=HSD17B4 PE=1 SV=3	DHB4_HUMAN	SwissProt_2014_08.fasta	79,688.50	99.80%	2	2	2	0.01%	4.48%
Uncategorized Sample	BioSample 1	(F001790)	Flotillin-2 OS=Homo sapiens GN=FLOT2 PE=1 SV=2	FLOT2_HUMAN	SwissProt_2014_08.fasta	47,063.90	100.00%	19	21	104	0.60%	43.70%
Uncategorized Sample	BioSample 1	(F001790)	Non-specific lipid-transfer protein OS=Homo sapiens GN=SCP2 PE=1 SV=2	NLTP_HUMAN	SwissProt_2014_08.fasta	58,994.60	100.00%	4	4	5	0.03%	7.50%
Uncategorized Sample	BioSample 1	(F001790)	2',3'-cyclic-nucleotide 3'-phosphodiesterase OS=Homo sapiens GN=CNP PE=1 SV=2	CN37_HUMAN	SwissProt_2014_08.fasta	47,580.60	100.00%	17	18	45	0.26%	35.60%
Uncategorized Sample	BioSample 1	(F001790)	Glial fibrillary acidic protein OS=Homo sapiens GN=GFAP PE=1 SV=1	GFAP_HUMAN	SwissProt_2014_08.fasta	49,881.40	100.00%	25	28	217	1.24%	50.20%
Uncategorized Sample	BioSample 1	(F001790)	Flotillin-1 OS=Homo sapiens GN=FLOT1 PE=1 SV=3	FLOT1_HUMAN	SwissProt_2014_08.fasta	47,354.80	100.00%	24	30	180	1.03%	61.60%
Uncategorized Sample	BioSample 1	(F001790)	Platelet-activating factor acetylhydrolase IB subunit alpha OS=Homo sapiens GN=PAFAH1B1 PE=1 SV=2	LIS1_HUMAN	SwissProt_2014_08.fasta	46,637.40	99.90%	2	2	2	0.01%	5.37%
Uncategorized Sample	BioSample 1	(F001790)	Tubulin alpha-4A chain OS=Homo sapiens GN=TUBA4A PE=1 SV=1	TBA4A_HUMAN	SwissProt_2014_08.fasta	49,924.60	99.80%	2	2	2	0.01%	30.60%
Uncategorized Sample	BioSample 1	(F001790)	Versican core protein OS=Homo sapiens GN=VCAN PE=1 SV=3	CSPG2_HUMAN	SwissProt_2014_08.fasta	372,812.60	100.00%	3	3	4	0.02%	1.09%
Uncategorized Sample	BioSample 1	(F001790)	Synaptic vesicle membrane protein VAT-1 homolog OS=Homo sapiens GN=VAT1 PE=1 SV=2	VAT1_HUMAN	SwissProt_2014_08.fasta	41,919.80	100.00%	2	2	3	0.02%	7.63%
Uncategorized Sample	BioSample 1	(F001790)	Creatine kinase B-type OS=Homo sapiens GN=CKB PE=1 SV=1	KCRB_HUMAN	SwissProt_2014_08.fasta	42,645.10	100.00%	3	3	4	0.02%	12.60%
Uncategorized Sample	BioSample 1	(F001790)	Actin, cytoplasmic 1 OS=Homo sapiens GN=ACTB PE=1 SV=1	ACTB_HUMAN,ACTG_HUMAN	SwissProt_2014_08.fasta	41,737.80	100.00%	6	6	25	0.14%	17.60%
Uncategorized Sample	BioSample 1	(F001790)	Tubulin alpha-1A chain OS=Homo sapiens GN=TUBA1A PE=1 SV=1	TBA1A_HUMAN	SwissProt_2014_08.fasta	50,135.70	100.00%	10	11	30	0.17%	32.40%
Uncategorized Sample	BioSample 1	(F001790)	PH and SEC7 domain-containing protein 3 OS=Homo sapiens GN=PSD3 PE=1 SV=2	PSD3_HUMAN	SwissProt_2014_08.fasta	116,035.90	100.00%	4	4	4	0.02%	6.11%
Uncategorized Sample	BioSample 1	(F001790)	Neurofilament medium polypeptide OS=Homo sapiens GN=NEFM PE=1 SV=3	NFM_HUMAN	SwissProt_2014_08.fasta	102,471.30	100.00%	14	14	31	0.18%	16.90%
Uncategorized Sample	BioSample 1	(F001790)	Vimentin OS=Homo sapiens GN=VIM PE=1 SV=4	VIME_HUMAN	SwissProt_2014_08.fasta	53,652.70	99.80%	2	2	2	0.01%	10.10%
Uncategorized Sample	BioSample 1	(F001790)	Elongation factor Tu, mitochondrial OS=Homo sapiens GN=TUFM PE=1 SV=2	EFTU_HUMAN	SwissProt_2014_08.fasta	49,542.40	100.00%	4	4	5	0.03%	10.40%
Uncategorized Sample	BioSample 1	(F001790)	Calcium/calmodulin-dependent protein kinase type II subunit alpha OS=Homo sapiens GN=CAMK2A PE=1 SV=2	KCC2A_HUMAN	SwissProt_2014_08.fasta	54,088.70	100.00%	14	15	153	0.88%	32.00%
Uncategorized Sample	BioSample 1	(F001791)	Pyruvate kinase PKM OS=Homo sapiens GN=PKM PE=1 SV=4	KPYM_HUMAN	SwissProt_2014_08.fasta	57,937.50	100.00%	13	14	32	0.18%	32.40%
Uncategorized Sample	BioSample 1	(F001791)	Succinyl-CoA:3-ketoacid coenzyme A transferase 1, mitochondrial OS=Homo sapiens GN=OXCT1 PE=1 SV=1	SCOT1_HUMAN	SwissProt_2014_08.fasta	56,158.80	99.80%	2	2	3	0.02%	7.50%
Uncategorized Sample	BioSample 1	(F001791)	TAR DNA-binding protein 43 OS=Homo sapiens GN=TARDBP PE=1 SV=1	TADBP_HUMAN	SwissProt_2014_08.fasta	44,739.70	100.00%	2	2	5	0.03%	7.25%

Uncategorized Sample	BioSample 1	(F001791)	Alpha-internexin OS=Homo sapiens GN=INA PE=1 SV=2	AINX_HUMAN	SwissProt_2014_08.fasta	55,392.30	100.00%	23	29	306	1.69%	53.30%
Uncategorized Sample	BioSample 1	(F001791)	Rab GDP dissociation inhibitor alpha OS=Homo sapiens GN=GDI1 PE=1 SV=2	GDIA_HUMAN	SwissProt_2014_08.fasta	50,584.10	100.00%	5	5	6	0.03%	21.50%
Uncategorized Sample	BioSample 1	(F001791)	Tubulin beta-4A chain OS=Homo sapiens GN=TUBB4A PE=1 SV=2	TBB4A_HUMAN	SwissProt_2014_08.fasta	49,585.50	100.00%	14	14	29	0.16%	44.60%
Uncategorized Sample	BioSample 1	(F001791)	Calcium/calmodulin-dependent protein kinase type II subunit delta OS=Homo sapiens GN=CAMK2D PE=1 SV=3	KCC2D_HUMAN	SwissProt_2014_08.fasta	56,370.70	100.00%	8	9	113	0.63%	28.10%
Uncategorized Sample	BioSample 1	(F001791)	Calcium/calmodulin-dependent protein kinase type II subunit beta OS=Homo sapiens GN=CAMK2B PE=1 SV=3	KCC2B_HUMAN	SwissProt_2014_08.fasta	72,678.70	100.00%	7	9	69	0.38%	23.90%
Uncategorized Sample	BioSample 1	(F001791)	Ubiquitin-60S ribosomal protein L40 OS=Homo sapiens GN=UBA52 PE=1 SV=2	RL40_HUMAN,RS27A_HUMAN,U	SwissProt_2014_08.fasta	14,728.90	100.00%	2	2	3	0.02%	19.50%
Uncategorized Sample	BioSample 1	(F001791)	Neurofilament light polypeptide OS=Homo sapiens GN=NEFL PE=1 SV=3	NFL_HUMAN	SwissProt_2014_08.fasta	61,517.80	100.00%	16	16	53	0.29%	42.70%
Uncategorized Sample	BioSample 1	(F001791)	Tubulin beta-2A chain OS=Homo sapiens GN=TUBB2A PE=1 SV=1	TBB2A_HUMAN	SwissProt_2014_08.fasta	49,907.10	100.00%	5	5	7	0.04%	39.80%
Uncategorized Sample	BioSample 1	(F001791)	Vimentin OS=Homo sapiens GN=VIM PE=1 SV=4	VIME_HUMAN	SwissProt_2014_08.fasta	53,652.70	100.00%	16	18	41	0.23%	35.80%
Uncategorized Sample	BioSample 1	(F001791)	Tubulin beta-4B chain OS=Homo sapiens GN=TUBB4B PE=1 SV=1	TBB4B_HUMAN	SwissProt_2014_08.fasta	49,830.70	100.00%	2	2	2	0.01%	37.10%
Uncategorized Sample	BioSample 1	(F001791)	Neurofilament medium polypeptide OS=Homo sapiens GN=NEFM PE=1 SV=3	NFM_HUMAN	SwissProt_2014_08.fasta	102,471.30	100.00%	14	15	31	0.17%	20.40%
Uncategorized Sample	BioSample 1	(F001791)	Calcium/calmodulin-dependent protein kinase type II subunit gamma OS=Homo sapiens GN=CAMK2G PE=1 SV=	KCC2G_HUMAN	SwissProt_2014_08.fasta	62,610.00	100.00%	4	5	17	0.09%	21.00%
Uncategorized Sample	BioSample 1	(F001791)	Glial fibrillary acidic protein OS=Homo sapiens GN=GFAP PE=1 SV=1	GFAP_HUMAN	SwissProt_2014_08.fasta	49,881.40	100.00%	9	9	19	0.11%	25.00%
Uncategorized Sample	BioSample 1	(F001791)	Microtubule-associated protein tau OS=Homo sapiens GN=MAPT PE=1 SV=5	TAU_HUMAN	SwissProt_2014_08.fasta	78,927.70	100.00%	7	9	24	0.13%	12.10%
Uncategorized Sample	BioSample 1	(F001791)	Dihydropyrimidinase-related protein 1 OS=Homo sapiens GN=CRMP1 PE=1 SV=1	DPYL1_HUMAN	SwissProt_2014_08.fasta	62,184.70	100.00%	4	4	8	0.04%	9.44%
Uncategorized Sample	BioSample 1	(F001791)	Dihydropyrimidinase-related protein 2 OS=Homo sapiens GN=DPYSL2 PE=1 SV=1	DPYL2_HUMAN	SwissProt_2014_08.fasta	62,294.00	100.00%	14	16	40	0.22%	42.80%
Uncategorized Sample	BioSample 1	(F001791)	Fibrinogen beta chain OS=Homo sapiens GN=FGB PE=1 SV=2	FIBB_HUMAN	SwissProt_2014_08.fasta	55,928.60	100.00%	3	3	3	0.02%	9.78%
Uncategorized Sample	BioSample 1	(F001791)	Vesicle-fusing ATPase OS=Homo sapiens GN=NSF PE=1 SV=3	NSF_HUMAN	SwissProt_2014_08.fasta	82,597.40	100.00%	3	3	3	0.02%	4.44%
Uncategorized Sample	BioSample 1	(F001791)	Versican core protein OS=Homo sapiens GN=VCAN PE=1 SV=3	CSPG2_HUMAN	SwissProt_2014_08.fasta	372,812.60	100.00%	5	7	36	0.20%	1.65%
Uncategorized Sample	BioSample 1	(F001791)	Copine-6 OS=Homo sapiens GN=CPNE6 PE=1 SV=3	CPNE6_HUMAN	SwissProt_2014_08.fasta	61,991.20	99.80%	2	2	2	0.01%	3.59%
Uncategorized Sample	BioSample 1	(F001791)	Propionyl-CoA carboxylase beta chain, mitochondrial OS=Homo sapiens GN=PCCB PE=1 SV=3	PCCB_HUMAN	SwissProt_2014_08.fasta	58,216.00	100.00%	4	4	4	0.02%	10.90%
Uncategorized Sample	BioSample 1	(F001791)	Dihydropyrimidinase-related protein 3 OS=Homo sapiens GN=DPYSL3 PE=1 SV=1	DPYL3_HUMAN	SwissProt_2014_08.fasta	61,964.10	100.00%	3	3	3	0.02%	18.90%
Uncategorized Sample	BioSample 1	(F001791)	Methylcrotonoyl-CoA carboxylase beta chain, mitochondrial OS=Homo sapiens GN=MCCC2 PE=1 SV=1	MCCB_HUMAN	SwissProt_2014_08.fasta	61,334.70	100.00%	6	7	17	0.09%	16.30%
Uncategorized Sample	BioSample 1	(F001791)	Cytosol aminopeptidase OS=Homo sapiens GN=LAP3 PE=1 SV=3	AMPL_HUMAN	SwissProt_2014_08.fasta	56,167.80	100.00%	2	2	2	0.01%	4.43%
Uncategorized Sample	BioSample 1	(F001791)	Endonuclease domain-containing 1 protein OS=Homo sapiens GN=ENDOD1 PE=1 SV=2	ENDD1_HUMAN	SwissProt_2014_08.fasta	55,017.60	100.00%	7	7	14	0.08%	21.80%
Uncategorized Sample	BioSample 1	(F001791)	Glucose-6-phosphate isomerase OS=Homo sapiens GN=GPI PE=1 SV=4	G6PI_HUMAN	SwissProt_2014_08.fasta	63,148.50	100.00%	3	3	6	0.03%	8.42%
Uncategorized Sample	BioSample 1	(F001791)	Protein kinase C gamma type OS=Homo sapiens GN=PRKCG PE=1 SV=3	KPCG_HUMAN	SwissProt_2014_08.fasta	78,448.70	100.00%	3	3	3	0.02%	6.74%
Uncategorized Sample	BioSample 1	(F001791)	Tubulin alpha-1A chain OS=Homo sapiens GN=TUBA1A PE=1 SV=1	TBA1A_HUMAN	SwissProt_2014_08.fasta	50,135.70	100.00%	3	3	3	0.02%	9.98%
Uncategorized Sample	BioSample 1	(F001791)	Keratin, type II cytoskeletal 2 epidermal OS=Homo sapiens GN=KRT2 PE=1 SV=2	K22E_HUMAN	SwissProt_2014_08.fasta	65,433.90	99.80%	2	2	2	0.01%	5.63%
Uncategorized Sample	BioSample 1	(F001791)	Keratin, type II cytoskeletal 1 OS=Homo sapiens GN=KRT1 PE=1 SV=6	K2C1_HUMAN	SwissProt_2014_08.fasta	66,040.30	100.00%	5	5	6	0.03%	8.70%
Uncategorized Sample	BioSample 1	(F001791)	Synaptotagmin-1 OS=Homo sapiens GN=SYT1 PE=1 SV=1	SYT1_HUMAN	SwissProt_2014_08.fasta	47,574.10	100.00%	2	2	2	0.01%	5.69%
Uncategorized Sample	BioSample 1	(F001791)	Protein RUFY3 OS=Homo sapiens GN=RUFY3 PE=1 SV=1	RUFY3_HUMAN	SwissProt_2014_08.fasta	52,967.10	99.80%	2	2	2	0.01%	4.69%
Uncategorized Sample	BioSample 1	(F001791)	Calcium/calmodulin-dependent protein kinase type II subunit alpha OS=Homo sapiens GN=CAMK2A PE=1 SV=2	KCC2A_HUMAN	SwissProt_2014_08.fasta	54,088.70	100.00%	9	11	87	0.48%	22.40%
Uncategorized Sample	BioSample 1	(F001792)	Alpha-internexin OS=Homo sapiens GN=INA PE=1 SV=2	AINX_HUMAN	SwissProt_2014_08.fasta	55,392.30	100.00%	5	5	7	0.04%	12.80%
Uncategorized Sample	BioSample 1	(F001792)	Calcium/calmodulin-dependent protein kinase type II subunit beta OS=Homo sapiens GN=CAMK2B PE=1 SV=3	KCC2B_HUMAN	SwissProt_2014_08.fasta	72,678.70	100.00%	2	2	2	0.01%	8.41%
Uncategorized Sample	BioSample 1	(F001792)	Ubiquitin-60S ribosomal protein L40 OS=Homo sapiens GN=UBA52 PE=1 SV=2	RL40_HUMAN,RS27A_HUMAN,U	SwissProt_2014_08.fasta	17,965.60	99.90%	2	2	3	0.02%	22.70%
Uncategorized Sample	BioSample 1	(F001792)	Neural cell adhesion molecule 1 OS=Homo sapiens GN=NCAM1 PE=1 SV=3	NCAM1_HUMAN	SwissProt_2014_08.fasta	94,574.30	100.00%	2	2	2	0.01%	4.43%
Uncategorized Sample	BioSample 1	(F001792)	Versican core protein OS=Homo sapiens GN=VCAN PE=1 SV=3	CSPG2_HUMAN	SwissProt_2014_08.fasta	372,812.60	100.00%	3	3	5	0.03%	1.21%
Uncategorized Sample	BioSample 1	(F001792)	Matrin-3 OS=Homo sapiens GN=MATR3 PE=1 SV=2	MATR3_HUMAN	SwissProt_2014_08.fasta	94,626.70	100.00%	4	4	5	0.03%	8.85%
Uncategorized Sample	BioSample 1	(F001792)	Heat shock protein HSP 90-alpha OS=Homo sapiens GN=HSP90AA1 PE=1 SV=5	HS90A_HUMAN	SwissProt_2014_08.fasta	84,663.20	100.00%	4	4	7	0.04%	6.83%
Uncategorized Sample	BioSample 1	(F001792)	Major vault protein OS=Homo sapiens GN=MVP PE=1 SV=4	MVP_HUMAN	SwissProt_2014_08.fasta	99,326.00	100.00%	15	16	21	0.13%	30.20%
Uncategorized Sample	BioSample 1	(F001792)	Neurofilament light polypeptide OS=Homo sapiens GN=NEFL PE=1 SV=3	NFL_HUMAN	SwissProt_2014_08.fasta	61,517.80	99.90%	2	2	3	0.02%	6.45%
Uncategorized Sample	BioSample 1	(F001792)	Peroxisomal multifunctional enzyme type 2 OS=Homo sapiens GN=HSD17B4 PE=1 SV=3	DHB4_HUMAN	SwissProt_2014_08.fasta	79,688.50	100.00%	3	3	3	0.02%	7.47%
Uncategorized Sample	BioSample 1	(F001792)	Neurofilament medium polypeptide OS=Homo sapiens GN=NEFM PE=1 SV=3	NFM_HUMAN	SwissProt_2014_08.fasta	102,471.30	100.00%	10	10	10	0.06%	18.60%
Uncategorized Sample	BioSample 1	(F001792)	Disks large homolog 4 OS=Homo sapiens GN=DLG4 PE=1 SV=3	DLG4_HUMAN	SwissProt_2014_08.fasta	80,497.30	100.00%	6	6	7	0.04%	16.70%
Uncategorized Sample	BioSample 1	(F001792)	Glial fibrillary acidic protein OS=Homo sapiens GN=GFAP PE=1 SV=1	GFAP_HUMAN	SwissProt_2014_08.fasta	49,881.40	100.00%	3	3	3	0.02%	7.18%
Uncategorized Sample	BioSample 1	(F001792)	Complement C4-A OS=Homo sapiens GN=C4A PE=1 SV=2	CO4A_HUMAN	SwissProt_2014_08.fasta	192,786.80	99.80%	2	2	2	0.01%	2.18%
Uncategorized Sample	BioSample 1	(F001792)	Calcium/calmodulin-dependent protein kinase type II subunit alpha OS=Homo sapiens GN=CAMK2A PE=1 SV=2	KCC2A_HUMAN	SwissProt_2014_08.fasta	54,088.70	100.00%	3	3	4	0.02%	8.79%
Uncategorized Sample	BioSample 1	(F001792)	Vesicle-fusing ATPase OS=Homo sapiens GN=NSF PE=1 SV=3	NSF_HUMAN	SwissProt_2014_08.fasta	82,597.40	100.00%	2	2	2	0.01%	3.76%
Uncategorized Sample	BioSample 1	(F001792)	Heat shock 70 kDa protein 12A OS=Homo sapiens GN=HSPA12A PE=1 SV=2	HS12A_HUMAN	SwissProt_2014_08.fasta	74,980.30	100.00%	2	2	2	0.01%	3.56%
Uncategorized Sample	BioSample 1	(F001792)	Dynamin-1 OS=Homo sapiens GN=DNM1 PE=1 SV=2	DYN1_HUMAN	SwissProt_2014_08.fasta	97,410.80	100.00%	3	3	3	0.02%	5.32%
Uncategorized Sample	BioSample 1	(F001792)	Keratin, type II cytoskeletal 1 OS=Homo sapiens GN=KRT1 PE=1 SV=6	K2C1_HUMAN	SwissProt_2014_08.fasta	66,040.30	99.80%	2	2	3	0.02%	3.73%
Uncategorized Sample	BioSample 1	(F001792)	Heterogeneous nuclear ribonucleoprotein U OS=Homo sapiens GN=HNRNPU PE=1 SV=6	HNRPU_HUMAN	SwissProt_2014_08.fasta	90,585.20	100.00%	4	4	4	0.02%	9.58%
Uncategorized Sample	BioSample 1	(F001793)	Cytoplasmic dynein 1 heavy chain 1 OS=Homo sapiens GN=DYNC1H1 PE=1 SV=5	DYHC1_HUMAN	SwissProt_2014_08.fasta	532,405.60	100.00%	4	4	4	0.03%	0.93%
Uncategorized Sample	BioSample 1	(F001793)	TAR DNA-binding protein 43 OS=Homo sapiens GN=TARDBP PE=1 SV=1	TADBP_HUMAN	SwissProt_2014_08.fasta	44,739.70	99.70%	2	2	3	0.02%	7.25%
Uncategorized Sample	BioSample 1	(F001793)	Glial fibrillary acidic protein OS=Homo sapiens GN=GFAP PE=1 SV=1	GFAP_HUMAN	SwissProt_2014_08.fasta	49,881.40	100.00%	7	7	9	0.06%	19.20%
Uncategorized Sample	BioSample 1	(F001793)	Microtubule-associated protein 2 OS=Homo sapiens GN=MAP2 PE=1 SV=4	MTAP2_HUMAN	SwissProt_2014_08.fasta	199,527.90	100.00%	2	2	3	0.02%	1.37%
Uncategorized Sample	BioSample 1	(F001793)	Tubulin beta-4A chain OS=Homo sapiens GN=TUBB4A PE=1 SV=2	TBB4A_HUMAN	SwissProt_2014_08.fasta	49,585.50	100.00%	2	2	3	0.02%	4.95%

Uncategorized Sample	BioSample 1	(F001793)	Neurofilament heavy polypeptide OS=Homo sapiens GN=NEFH PE=1 SV=4	NFH_HUMAN	SwissProt_2014_08.fasta	112,480.10	100.00%	3	3	5	0.03%	4.09%
Uncategorized Sample	BioSample 1	(F001793)	Ubiquitin-60S ribosomal protein L40 OS=Homo sapiens GN=UBA52 PE=1 SV=2	RL40_HUMAN,RS27A_HUMAN,U	SwissProt_2014_08.fasta	14,728.90	100.00%	2	2	22	0.14%	22.70%
Uncategorized Sample	BioSample 1	(F001793)	Neural cell adhesion molecule 1 OS=Homo sapiens GN=NCAM1 PE=1 SV=3	NCAM1_HUMAN	SwissProt_2014_08.fasta	94,574.30	100.00%	5	5	5	0.03%	9.67%
Uncategorized Sample	BioSample 1	(F001793)	IQ motif and SEC7 domain-containing protein 2 OS=Homo sapiens GN=IQSEC2 PE=1 SV=1	IQEC2_HUMAN	SwissProt_2014_08.fasta	161,738.20	100.00%	5	5	6	0.04%	4.80%
Uncategorized Sample	BioSample 1	(F001793)	Microtubule-associated protein 1B OS=Homo sapiens GN=MAP1B PE=1 SV=2	MAP1B_HUMAN	SwissProt_2014_08.fasta	270,634.40	100.00%	2	2	2	0.01%	1.18%
Uncategorized Sample	BioSample 1	(F001793)	Contactin-1 OS=Homo sapiens GN=CNTN1 PE=1 SV=1	CNTN1_HUMAN	SwissProt_2014_08.fasta	113,322.80	100.00%	3	3	3	0.02%	3.24%
Uncategorized Sample	BioSample 1	(F001793)	Plectin OS=Homo sapiens GN=PLEC PE=1 SV=3	PLEC_HUMAN	SwissProt_2014_08.fasta	531,783.90	100.00%	11	11	12	0.08%	2.88%
Uncategorized Sample	BioSample 1	(F001793)	Sodium/potassium-transporting ATPase subunit alpha-1 OS=Homo sapiens GN=ATP1A1 PE=1 SV=1	AT1A1_HUMAN	SwissProt_2014_08.fasta	112,899.50	100.00%	5	5	5	0.03%	6.35%
Uncategorized Sample	BioSample 1	(F001793)	Complement C4-A OS=Homo sapiens GN=C4A PE=1 SV=2	CO4A_HUMAN	SwissProt_2014_08.fasta	192,786.80	100.00%	5	5	7	0.05%	4.99%
Uncategorized Sample	BioSample 1	(F001793)	SH3 and multiple ankyrin repeat domains protein 3 OS=Homo sapiens GN=SHANK3 PE=1 SV=3	SHAN3_HUMAN	SwissProt_2014_08.fasta	184,671.50	99.80%	2	2	2	0.01%	2.08%
Uncategorized Sample	BioSample 1	(F001793)	Spectrin beta chain, non-erythrocytic 1 OS=Homo sapiens GN=SPTBN1 PE=1 SV=2	SPTB2_HUMAN	SwissProt_2014_08.fasta	274,613.40	100.00%	16	16	16	0.10%	8.88%
Uncategorized Sample	BioSample 1	(F001793)	Collagen alpha-3(VI) chain OS=Homo sapiens GN=COL6A3 PE=1 SV=5	CO6A3_HUMAN	SwissProt_2014_08.fasta	343,667.40	100.00%	3	3	3	0.02%	1.29%
Uncategorized Sample	BioSample 1	(F001793)	Collagen alpha-1(VI) chain OS=Homo sapiens GN=COL6A1 PE=1 SV=3	CO6A1_HUMAN	SwissProt_2014_08.fasta	108,531.00	100.00%	3	3	3	0.02%	3.60%
Uncategorized Sample	BioSample 1	(F001793)	Neurofilament medium polypeptide OS=Homo sapiens GN=NEFM PE=1 SV=3	NFM_HUMAN	SwissProt_2014_08.fasta	102,471.30	100.00%	30	34	117	0.76%	40.50%
Uncategorized Sample	BioSample 1	(F001793)	Tenascin-R OS=Homo sapiens GN=TNR PE=1 SV=3	TENR_HUMAN	SwissProt_2014_08.fasta	149,560.40	100.00%	2	2	3	0.02%	2.36%
Uncategorized Sample	BioSample 1	(F001793)	Ferritin light chain OS=Homo sapiens GN=FTL PE=1 SV=2	FRIL_HUMAN	SwissProt_2014_08.fasta	20,020.60	100.00%	6	9	41	0.27%	32.60%
Uncategorized Sample	BioSample 1	(F001793)	Ferritin heavy chain OS=Homo sapiens GN=FTH1 PE=1 SV=2	FRIH_HUMAN	SwissProt_2014_08.fasta	21,226.20	100.00%	12	14	97	0.63%	46.40%
Uncategorized Sample	BioSample 1	(F001793)	2',3'-cyclic-nucleotide 3'-phosphodiesterase OS=Homo sapiens GN=CNP PE=1 SV=2	CN37_HUMAN	SwissProt_2014_08.fasta	47,580.60	100.00%	4	4	4	0.03%	13.50%
Uncategorized Sample	BioSample 1	(F001793)	Microtubule-associated protein tau OS=Homo sapiens GN=MAPT PE=1 SV=5	TAU_HUMAN	SwissProt_2014_08.fasta	78,927.70	100.00%	3	3	4	0.03%	5.01%
Uncategorized Sample	BioSample 1	(F001793)	Collagen alpha-2(VI) chain OS=Homo sapiens GN=COL6A2 PE=1 SV=4	CO6A2_HUMAN	SwissProt_2014_08.fasta	108,580.80	100.00%	3	3	3	0.02%	3.34%
Uncategorized Sample	BioSample 1	(F001793)	Versican core protein OS=Homo sapiens GN=VCAN PE=1 SV=3	CSPG2_HUMAN	SwissProt_2014_08.fasta	372,812.60	100.00%	5	5	6	0.04%	2.18%
Uncategorized Sample	BioSample 1	(F001793)	Laminin subunit beta-2 OS=Homo sapiens GN=LAMB2 PE=1 SV=2	LAMB2_HUMAN	SwissProt_2014_08.fasta	195,975.90	100.00%	4	4	5	0.03%	2.61%
Uncategorized Sample	BioSample 1	(F001793)	Actin, cytoplasmic 1 OS=Homo sapiens GN=ACTB PE=1 SV=1	ACTB_HUMAN,ACTG_HUMAN	SwissProt_2014_08.fasta	41,737.80	100.00%	2	2	2	0.01%	7.47%
Uncategorized Sample	BioSample 1	(F001793)	Prolow-density lipoprotein receptor-related protein 1 OS=Homo sapiens GN=LRP1 PE=1 SV=2	LRP1_HUMAN	SwissProt_2014_08.fasta	504,591.80	100.00%	3	3	3	0.02%	0.84%
Uncategorized Sample	BioSample 1	(F001793)	Fibronectin OS=Homo sapiens GN=FN1 PE=1 SV=4	FINC_HUMAN	SwissProt_2014_08.fasta	262,616.90	100.00%	5	5	6	0.04%	3.10%
Uncategorized Sample	BioSample 1	(F001793)	Keratin, type II cytoskeletal 1 OS=Homo sapiens GN=KRT1 PE=1 SV=6	K2C1_HUMAN	SwissProt_2014_08.fasta	66,040.30	100.00%	3	3	4	0.03%	5.59%
Uncategorized Sample	BioSample 1	(F001793)	Neurofilament light polypeptide OS=Homo sapiens GN=NEFL PE=1 SV=3	NFL_HUMAN	SwissProt_2014_08.fasta	61,517.80	100.00%	2	2	2	0.01%	8.47%
Uncategorized Sample	BioSample 1	(F001793)	Calcium/calmodulin-dependent protein kinase type II subunit alpha OS=Homo sapiens GN=CAMK2A PE=1 SV=2	KCC2A_HUMAN	SwissProt_2014_08.fasta	54,088.70	100.00%	5	5	6	0.04%	13.40%
